# Application of Euler Neural Networks with Soft Computing Paradigm to Solve Nonlinear Problems Arising in Heat Transfer

**DOI:** 10.3390/e23081053

**Published:** 2021-08-16

**Authors:** Naveed Ahmad Khan, Osamah Ibrahim Khalaf, Carlos Andrés Tavera Romero, Muhammad Sulaiman, Maharani A. Bakar

**Affiliations:** 1 Department of Mathematics, Abdul Wali Khan University Mardan, Mardan 23200, KP, Pakistan; ahmednaveed854477@gmail.com; 2 Al-Nahrain Nanorenewable Energy Research Center Baghdad, Al-Nahrain University, Baghdad 10001, Iraq; usama.ibrahem@coie-nahrain.edu.iq; 3 COMBA R&D Laboratory, Faculty of Engineering, Universidad Santiago de Cali, Cali 76001, Colombia; carlos.tavera00@usc.edu.co; 4 Faculty of Ocean Engineering Technology and Informatics, Universiti Malaysia Terengganu, Kuala Nerus 21300, Terengganu, Malaysia; maharani@umt.edu.my

**Keywords:** heat transfer problems, nonlinear differential equations, variable specific heat coefficient, lumped system, Euler neural networks, hybrid soft computing, generalized normal distribution optimization, interior point algorithm

## Abstract

In this study, a novel application of neurocomputing technique is presented for solving nonlinear heat transfer and natural convection porous fin problems arising in almost all areas of engineering and technology, especially in mechanical engineering. The mathematical models of the problems are exploited by the intelligent strength of Euler polynomials based Euler neural networks (ENN’s), optimized with a generalized normal distribution optimization (GNDO) algorithm and Interior point algorithm (IPA). In this scheme, ENN’s based differential equation models are constructed in an unsupervised manner, in which the neurons are trained by GNDO as an effective global search technique and IPA, which enhances the local search convergence. Moreover, a temperature distribution of heat transfer and natural convection porous fin are investigated by using an ENN-GNDO-IPA algorithm under the influence of variations in specific heat, thermal conductivity, internal heat generation, and heat transfer rate, respectively. A large number of executions are performed on the proposed technique for different cases to determine the reliability and effectiveness through various performance indicators including Nash–Sutcliffe efficiency (NSE), error in Nash–Sutcliffe efficiency (ENSE), mean absolute error (MAE), and Thiel’s inequality coefficient (TIC). Extensive graphical and statistical analysis shows the dominance of the proposed algorithm with state-of-the-art algorithms and numerical solver RK-4.

## 1. Introduction

Most of the problems in engineering sciences, especially heat transfer problems, are inherently nonlinear. Except for a limited number of these problems, most of them cannot be solved analytically by using traditional techniques. Linear and nonlinear differential equations were generally solved by integral transformation methods such as the Fourier or Laplace transform. These techniques are used to convert differential equations into a corresponding algebraic system of equations. Nonetheless, applying integral transformation methods was challenging at times [1]. In the 19th century, researchers such as Bellman [2], Cole [3], and O’Malley used the perturbation approach for solving nonlinear problems. In perturbation methods, the choice of small parameters and their exertion in differential equations was one of the challenging tasks for the research community. Nayfeh [4] and Van Dyke [5] improved the method by working on the loss of small parameters during physical verification. Later on, various powerful techniques have been developed to eliminate the small parameters such as the tanh method introduced by Wazwaz [6], artificial parameter method (APM) [7,8], homotopy analysis method (HAM) [9,10], homotopy perturbation method (HPM) [11], modified homotopy perturbation method (MHPM) [12], and iteration perturbation method (IPM) [13]. The application of these methods in the field of fluid dynamics, mechanical engineering, and heat radiation was studied by Rajabi [14], Abbasbandy, and Nadim [15,16]. Nonlinear problems arising in the heat transfer problem has been solved by Yaghoobi and Torabi [1] using the differential transformation method (DTM). The variational iteration method (VIM) was implemented by [17] to study the analytical solution for nonlinear problems. Recently, Kumbinarasaiaha [18,19] uses the Hermite wavelet method (HWM) to investigate the convecting-radiating and cooling of a lumped system with variable specific heat. Several Lagrangian based and ISPH methods are also used to study the heat transfer of various problems arising in fluid dynamics such as double-diffusive natural convection [20], a nanofluid-filled cavity including rotating solid structures [21], trapezoidal cooling microchannel [22], solid particles in an inner cross shape [23], circular enclosure partially saturated with a porous medium [24], nanofluid in a cavity with a partially layered porous medium [25,26], sloshing porous cavity filled with a nanofluid [27,28], and magneto-convective flow of a ferrofluid in a closed space [29,30].

Heat transfer in fins, also known as extended surfaces, has been a subject of interest, which has led to extensive research on the use of porous fins. Kiwan and Al-Nimr [31,32] were the first to investigate the use of porous materials to enhance heat transfer. Kiwan studies the performance of fins in a natural convection environment while Al-Nimr developed a numerical method to analyze the thermal analysis of fins [33]. Analytical models and optimization of porous fins were studied by Kundu and Bhanja [34]. In the last decade, the number of researchers numerically investigate the heat transfer of a rectangular porous fin [35], constructal T-shape porous fin [36], pin fins [37,38], cylindrical porous fins [39], and porous radial fins [40] under different environments. In 2013, Gorla [41,42] studies the influence of variations in thermal conductivity on natural convection and radiation in porous fins. To solve the heat transfer problems in porous fins, Saedodin and Sadeghi [39] applied the Runge–Kutta method for thermal analysis in fins. Kundu [37] investigates optimum design analysis by applying the Adomian decomposition method (ADM). To study heat transfer in longitudinal fins, Darvishi [40] and Moradi [43] adopted the homotopy analysis method (HAM). Several other techniques including the homotopy perturbation method (HPM) [44], spectral collocation method (SCM) [45,46], least square method (LSM) [47,48], variational iterative method (VIM) [49], and differential transformation method (DTM) [50] are used to study the temperature distribution and heat transfer of different fin problems. All of these methods are based on deterministic approaches and have their own merits, applicability, and drawbacks.

In recent times, intelligence-based nature-inspired meta heuristic algorithms have gained the attention of researchers. Some recent applications of soft computing techniques include the saturation process of water and oil through a porous medium during secondary oil recovery [51], heat transfer prediction of supercritical water [52], physics-informed neural networks [53], temperature profiles in longitudinal fin designs [54], wire coating dynamics [55,56], data-driven modeling for boiling heat [57], prediction of turbulent heat transfer [58], the corneal model for eye surgery [59], fuzzy systems [60], infrared, boiling heat transfer investigations [61], neuro-fuzzy modeling is used to predict the summer precipitation in targeted metrological sites [62], and prediction of heat transfer rates for shell-and-tube heat exchangers [63], beam-column designs [64], and nonlinear dusty plasma systems are analyzed with the help of NAR-RBFs neural networks [65]. The plant prorogation algorithm (PPA) and improved PPA are developed to solve a number of constrained and unconstrained engineering optimization problems [66,67]. The above-mentioned algorithms and their applications motivate the authors to build an efficient, reliable, and stable algorithm for analytical solutions of nonlinear heat transfer problems. Salient features of the given study are summarized as

- A novel soft computing paradigm is developed to analyze the heat transfer problems arising in mechanical engineering. Euler polynomials based Euler neural networks (ENN’s) are constructed to define an unsupervised differential equation model for different problems. Neurons in ENN are optimized by the hybridization of generalized normal distribution (GNDO) algorithm and interior-point algorithm (IPA). The proposed methodology is called the ENN-GNDO-IPA.
- The efficiency and correctness of the design scheme are ascertained by comparing its results with state-of-the-art algorithms and a numerical solver based on RK-4 for each case of different scenarios and problems.
- To validate the consistency of accuracy and convergence, the proposed algorithm is simulated to obtain a statistical results in terms of Nash–Sutcliffe efficiency (NSE), error in Nash–Sutcliffe efficiency (ENSE), mean absolute error (MAE), and Thiel’s inequality coefficient (TIC).
- Statistical inferences through minimum, mean, and standard deviations for fitness function and performance indicators further validate the worth of intelligent stochastic strategy in terms of complexity and accuracy.

## 2. Problems Formulation

### 2.1. Convecting-Radiating Cooling of a Lumped System with Variable Specific Heat

Consider the problem of combined convective–radiative cooling of a lumped system [18]. Let *V* and *A* be the volume and surface area of the system with specific heat density ρ and $T_{i}$ be the initial temperature. At t=0, the system is subjected to the convective environment with temperature $\left(T_{a}\right)$ and the coefficient of convective heat transfer is *h*. In addition, the system loses heat through radiation, and $T_{s}$ denotes the effective sink temperature. Specific heat is denoted by *C* and is defined as

(1) $$C=C_{a}\left[1+\bar{\zeta }\left(T-T_{a}\right)\right],$$

$C_{a}$ is a specific heat at temperature $T_{a}$ and $\bar{\zeta }$ is invariable (constant). Using the heat conduction equation, the cooling equation and its initial condition are derived as

(2) $$\rho VC\frac{dT}{dt}+hA\left(T-T_{a}\right)+EA\sigma \left(T^{4}-T_{s}^{4}\right)=0,\;T\left(0\right)=T_{i}.$$

To solve Equation (2), the following parameters are defined:

(3) $$y=\frac{T}{T_{i}},\;y_{a}=\frac{T_{a}}{T_{i}},\;y_{s}=\frac{T_{s}}{T_{i}},\;\varepsilon_{1}=\bar{\zeta }T_{i},\;\varepsilon_{2}=\frac{E\sigma T_{i}^{3}}{h},\;x=\frac{t}{\rho VC_{a}/hA}.$$

After modification of parameters, the heat transfer equation will result in the following:

(4) $$\left[1+\varepsilon_{1}\left(y-y_{a}\right)\right]\frac{dy}{dx}+\left(y-y_{a}\right)+\varepsilon_{2}\left(y^{4}-y_{s}^{4}\right)=0,$$

at

(5) x=0,y=1.

For simplicity, we assume the case when $y_{a}=y_{s}=0$; then, we have

(6) $$\left[1+\varepsilon_{1}y\right]\frac{dy}{dx}+y+\varepsilon_{2}y^{4}=0,$$

with initial condition

(7) y(0)=1.

### 2.2. Cooling of a Lumped System with Variable Specific Heat

Consider the cooling of a lumped system [18]. From Equation (1), specific heat is given as

(8) $$C=C_{a}\left[1+\bar{\zeta }\left(T-T_{a}\right)\right].$$

$C_{a}$ is a specific heat at temperature $T_{a}$ and $\bar{\zeta }$ is invariable (constant). Using the heat conduction equation, the cooling equation and its initial condition can be written as

(9) $$\rho CV\frac{dT}{dt}+hA\left(T-T_{a}\right)=0,$$

with

(10) $$T\left(0\right)=T_{i},$$

to solve Equation (9), the following parameters are defined as

(11) $$y=\frac{T-T_{a}}{T_{i}-T_{a}},\;x=\frac{t}{\rho CV/hA},\;\varepsilon =\bar{\zeta }\left(T_{i}-T\right).$$

Subsequently, the governing differential equation reduced to

(12) $$\frac{dy}{dx}+\varepsilon y\frac{dy}{dx}+y=0$$

with initial condition

(13) y(0)=1.

### 2.3. Natural Convection Porous Fin with Temperature-Dependent Thermal Conductivity and Internal Heat Generation

**Figure 1:**
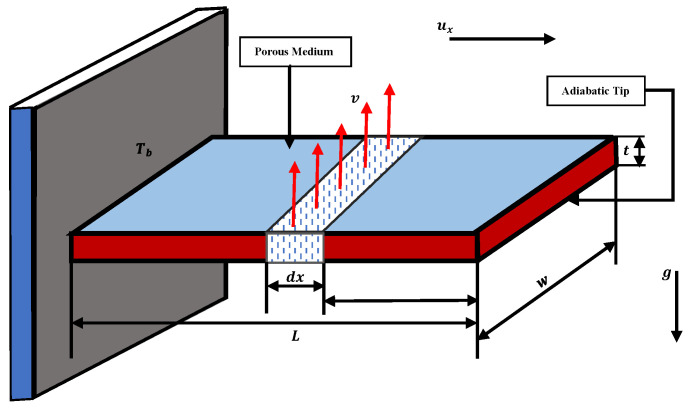
Schematic of the straight porous fin geometry with the internal heat generation.

Consider a straight porous fin with a length *L* and a thickness *t* that is exposed to a convective environment on both sides with temperature $T_{\infty }$, as shown in Figure 1. *x* represents the height of the fin having a base at the origin. To briefly analyze the problem, the following assumptions are considered:

(a) Porous medium of a straight fin is isotropic, homogenous, and saturated with fluid in a single phase.
(b) Physical parameters except the density of fluid and solid are considered to be constant.
(c) Radiative transfers, surface convection, and non-Darcian effects are small (negligible), and only natural convection is considered. In addition, the fin base is responsible for the transfer of heat through pores i.e., no convective heat is transmitted to the surrounding environment.
(d) Porous medium and fluid are in thermodynamic equilibrium.
(e) Variation of the temperature inside the fin is one-dimensional. Temperature changes with the length and remains static with time.
(f) Tip of the fin is adiabatic, and the base of the fin is isolated.

Using the above assumptions, thermal energy based on Darcy’s model can be expressed as

(14) $$q_{x}-\left(q_{x}+\frac{\delta q}{\delta x}dx\right)=\rho c_{p}v\left(x\right)w\left(T-T_{c}\right)dx+q_{\mathrm{int}.\;}\left(T\right)A_{c\tau }dx.$$

The velocity v(x) of buoyancy flow in the fin at any point *x* is obtained by using Darcy’s law

(15) $$v\left(x\right)=\frac{g\beta^{\prime }K}{v_{f}}\left(T-T_{\infty }\right),$$

(16) $$q_{x}-\left(q_{x}+\frac{\delta q}{\delta x}dx\right)=\frac{\rho c_{p}g\beta^{\prime }K}{v_{f}}w{\left(T-T_{\infty }\right)}^{2}dx+q_{\mathrm{int}.\;}\left(T\right)A_{cr}dx,$$

for dx→0, Equation (16) can be written as

(17) $$\frac{dq}{dx}+\frac{\rho c_{p}g\beta^{\prime }K}{v_{f}}w{\left(T-T_{\infty }\right)}^{2}+q_{int.}\left(T\right)A_{cr}=0,$$

from Fourier’s law of heat conduction

(18) $$q+k\left(T\right)A_{cr}\frac{dT}{dx}=0,$$

Using Equation (18) in Equation (17), we get

(19) $$\frac{d}{dx}\left(k\left(T\right)A_{cT}\frac{dT}{dx}\right)=\frac{\rho c_{p}g\beta^{\prime }Kw}{v_{f}}{\left(T-T_{\infty }\right)}^{2}+q_{in}\left(T\right)A_{cr},$$

Governing equation of fin is obtained by simplification of Equation (19), we have

(20) $$\frac{d}{dx}\left[k_{eff}\left(T\right)\frac{dT}{dx}\right]-\frac{\rho c_{p}g\beta^{\prime }K{\left(T-T_{\infty }\right)}^{2}}{tv_{f}}+q_{a}\left(T\right)=0,$$

subjected to boundary conditions given as

(21) $$\begin{array}{cc} x=L, & T=T_{b},\\ x=0, & \frac{dT}{dx}=0. \end{array}$$

Internal heat generation and temperature-dependent thermal conductivity are defined as

(22) $$q_{\mathrm{int}}\left(T\right)=q_{a}\left[1+\psi \left(T-T_{\infty }\right)\right],$$

(23) $$k_{eff}\left(T\right)=\phi k_{f}+\left(1-\phi \right)k_{s}=k_{eff,a}\left[1+\lambda \left(T-T_{\infty }\right)\right],$$

Using Equations (22) and (23) in Equation (14), we get

(24) $$\frac{d}{dx}\left[\left[1+\lambda \left(T-T_{\infty }\right)\right]\frac{dT}{dx}\right]-\frac{\rho c_{p}gK\beta^{\prime }{\left(T-T_{\infty }\right)}^{2}}{k_{eff,a}tv_{f}}+\frac{q_{a}}{k_{eff,a}}\left[1+\psi \left(T-T_{\infty }\right)\right]=0.$$

Introduce the following dimensionless parameters:

$$X=\frac{x}{L},\;y=\frac{T-T_{\infty }}{T_{b}-T_{\infty }},\;Ra=Gr\cdot \mathrm{Pr}=\left(\frac{\beta^{\prime }gT_{b}t^{3}}{v_{f}^{2}}\right)\left(\frac{\rho c_{p}v_{f}}{k_{eff,a}}\right),$$

$$Da=\frac{K}{t^{2}},\;Q=\frac{qv_{f}t}{\rho c_{p}\beta^{\prime }gK{\left(T_{b}-T_{\infty }\right)}^{2}},\;\gamma =\psi \left(T_{b}-T_{\infty }\right),$$

$$S_{h}=\left(\frac{\beta^{\prime }g\left(T_{b}-T_{\infty }\right)t^{3}}{v_{f}^{2}}\right)\left(\frac{\rho c_{p}v_{f}K}{k_{eff,a}t^{2}}\right)\frac{{\left(L/t\right)}^{2}}{k_{eff,a}}=\frac{RaDa{\left(L/t\right)}^{2}}{k_{eff,a}},\;\beta =\lambda \left(T_{b}-T_{\infty }\right).$$

The following dimensionless governing differential equation of the model is obtained:

(25) $$\frac{d}{dX}\left[\left(1+\beta y\right]\frac{dy}{dX}\right]-S_{h}y^{2}+S_{h}Q\left(1+\gamma y\right)=0.$$

From simplification of Equation (25), we get the differential equation of the form with boundary conditions given as

(26) $$\frac{d^{2}y}{dX^{2}}+\beta y\frac{d^{2}y}{dX^{2}}+\beta {\left(\frac{dy}{dX}\right)}^{2}-S_{h}y^{2}+S_{h}Q\gamma y+S_{h}Q=0,$$

(27) $$\begin{array}{cc} X=1, & y=1,\\ X=0, & \frac{dy}{dX}=0. \end{array}$$

### 2.4. Metallic Annular Fin with Temperature Dependent Thermal Conductivity

**Figure 2:**
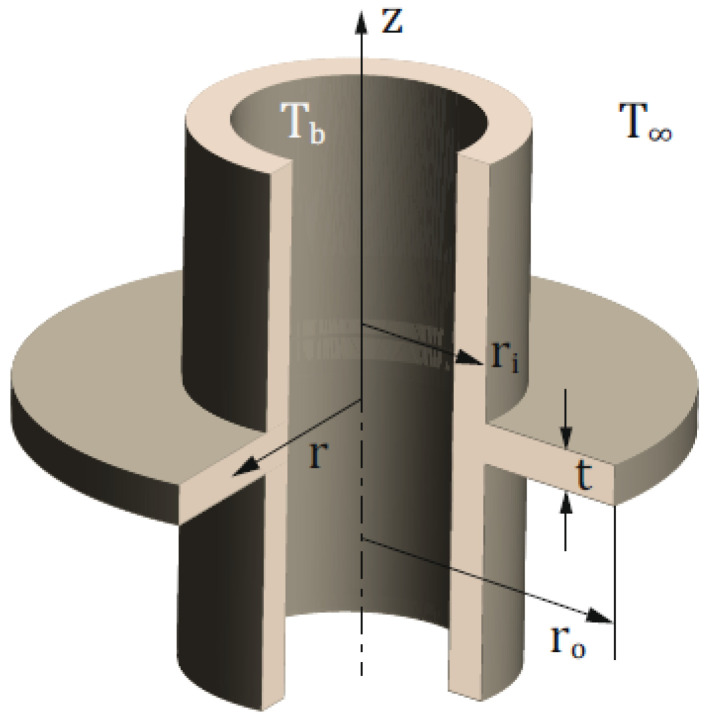
Geometry of metallic annular fin.

In this problem, we consider an optimal design of metallic annular fin as shown in Figure 2, having uniform thickness (t). The fin is attached to the heat transfer surface (base) of the heat exchanger. This exchanger is exposed to the surrounding with ambient temperature $T_{\infty }$ and temperature-dependent thermal conductivity. The phenomenon causes a mixed conductive-convective heat exchange with the air. To derive the governing equation for annular fin, we assume that the coefficient of heat transfer (h) is constant, and the exchanger is quite symmetric. Temperature distribution within fins can be mathematically presented as [68]

(28) $$t\frac{d}{\;dr}\left(k\left(T\right)r\frac{dT}{\;dr}\right)=2hr\left(T-T_{\infty }\right),$$

with

$$T\left(r_{i}\right)=T_{b},\;\frac{dT}{\;dr}\left(r_{o}\right)=0.$$

Furthermore, temperature dependent thermal conductivity of the fin is defined as

(29) $$k\left(T\right)=k_{\infty }\left[1+\kappa \left(T-T_{\infty }\right)\right],$$

where $k_{\infty }$ denotes the thermal conductivity at ambient temperature and κ is constant. In order to obtain the corresponding boundary value problem, we define the following dimensionless variables:

$$y=\frac{T-T_{\infty }}{T_{b}-T_{\infty }},\;Bi=\frac{hr_{i}}{k_{\infty }},\;\beta =\kappa \left(T_{b}-T_{\infty }\right),\;\xi =\frac{r-{}_{i}}{r_{i}},\;\lambda =\frac{r_{o}}{r_{i}},\;\delta =\frac{t}{r_{i}},$$

Now, Equation (28) along with boundary conditions will reduce to

(30) $$\frac{d^{2}y}{d\xi^{2}}+\beta {\left(\frac{dy}{d\xi }\right)}^{2}+\beta y\frac{d^{2}y}{d\xi^{2}}+\frac{\beta }{\left(1+\xi \right)}y\frac{dy}{d\xi }+\frac{1}{\left(1+\xi \right)}\frac{dy}{d\xi }-\frac{2Bi}{\delta }y=0,\;0<\xi <\lambda -1,$$

subjected to

(31) $$y\left(0\right)=1,\;\frac{dy}{d\xi }\left(\lambda -1\right)=0.$$

To solve this problem, we focus our attention on the interval [0,1]; therefore, we define a variable ξ=(λ−1)x. Now, Equation (30) can be written as

(32) $$\frac{d^{2}y}{dx^{2}}+\beta {\left(\frac{dy}{dx}\right)}^{2}+\beta y\frac{d^{2}y}{dx^{2}}+\frac{\beta (\lambda -1)}{\left(1+(\lambda -1)x\right)}y\frac{dy}{dx}+\frac{\left(\lambda -1\right)}{\left(1+(\lambda -1)x\right)}\frac{dy}{dx}-\frac{2Bi{\left(\lambda -1\right)}^{2}}{\delta }y=0,0<x<1,$$

(33) $$y\left(0\right)=1,\;\frac{dy}{dx}\left(1\right)=0.$$

## 3. Methodology

**Figure 3:**
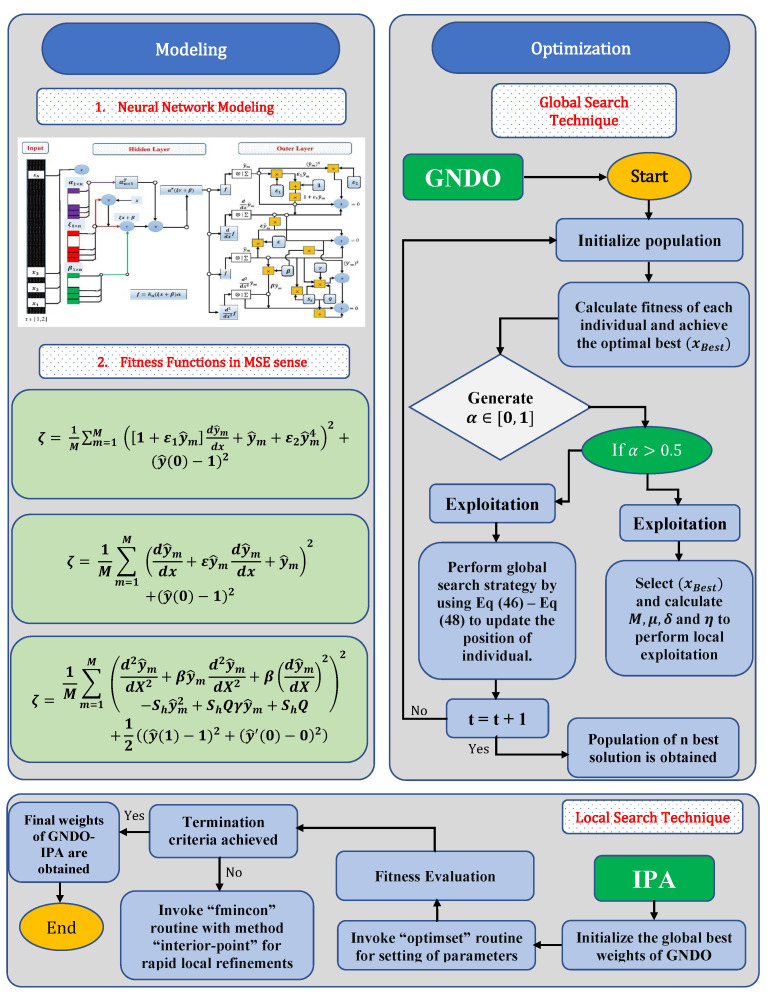
Graphical overview of the ENN-GNDO-IPA Algorithm.

The design methodology is comprised of two steps. Initially, feed forward artificial neural networks are used to construct a mean square error (MSE) based fitness function for a mathematical model of heat transfer and natural convection porous fin with temperature-dependent thermal conductivity and internal heat generation. In the second part, the learning procedure is provided for finding solutions of the model using memetic computing in which GNDO is used as a global search technique while IPA is used for the refinement of the local procedure. The designed algorithm ENN-GNDO-IPA is utilized as an optimization mechanism for unknown neurons in the ENN model. The flow chart of the procedure is shown in Figure 3.

### 3.1. Euler Polynomials and ENN Modeling

The classical Euler polynomials are denoted by $E_{n}$ and generally defined by the means of an exponential generating function given as

(34) $$\frac{2e^{xt}}{e^{t}+1}=\sum_{n=0}^{\infty }E_{n}\left(x\right)\frac{t^{n}}{n!},$$

and, explicitly, Euler polynomials are defined as

(35) $$E_{n}\left(x\right)=\frac{1}{n+1}\sum_{k=1}^{n+1}\left(2-2^{k+1}\right)\left(\begin{array}{c} n+1\\ k \end{array}\right)B_{k}x^{n+1-k},$$

where $B_{k}$ is Bernoulli number for each *k*. First, seven Euler polynomials are given as

$$E_{0}\left(x\right)=1,\;E_{1}\left(x\right)=x-\frac{1}{2},\;E_{2}\left(x\right)=x^{2}-x,\;E_{3}\left(x\right)=x^{3}-\frac{3}{2}x^{2}+\frac{1}{4},$$

$$E_{4}\left(x\right)=x^{4}-2x^{3}+x,\;E_{5}\left(x\right)=x^{5}-\frac{5}{2}x^{4}+\frac{5}{2}x^{2}-\frac{1}{2},\;E_{7}\left(x\right)=x^{6}-3x^{5}+5x^{3}-3x,$$

Some interesting properties and relations about Euler polynomials can be found in [69,70].

A trial or approximate solution $\hat{y}\left(x\right)$ along with first $\hat{y^{\prime }}\left(x\right)$ and second $\hat{y^{\prime \prime }}\left(x\right)$ derivatives are considered based on feed-forward artificial neural networks as

(36) $$\hat{y}\left(x\right)=\sum_{j=1}^{n}\alpha_{j}f\left(\xi_{j}x+\beta_{j}\right),$$

(37) $$\hat{y^{\prime }}\left(x\right)=\sum_{j=1}^{n}\alpha_{j}f^{\prime }\left(\xi_{j}x+\beta_{j}\right),$$

and

(38) $$\hat{y^{\prime \prime }}\left(x\right)=\sum_{j=1}^{n}\alpha_{j}f^{\prime \prime }\left(\xi_{j}x+\beta_{j}\right),$$

where α, ξ and β are unknown vectors of *W* as

(39) $$\begin{array}{cc} W & =[\alpha ,\xi ,\beta ],\\ \; & =\left[\alpha_{1},\alpha_{2},\ldots ,\alpha_{m},\xi_{1},\xi_{2},\ldots ,\xi_{m},\beta_{1},\beta_{2},\ldots ,\beta_{m}\right]. \end{array}$$

In order to find approximate solution for the mathematical models, Euler polynomials are used as an activation function i.e.,

$$f\left(x\right)=E_{0}\left(x\right)+E_{1}\left(x\right)+E_{2}\left(x\right)+\cdots +E_{7}\left(x\right).$$

Thus, the updated manifestation for the networks becomes

(40) $$\hat{y}\left(x\right)=\sum_{j=1}^{n}\alpha_{j}\sum_{n=0}^{6}E_{n}\left(\xi_{j}x+\beta_{j}\right),$$

(41) $$\hat{y^{\prime }}\left(x\right)=\sum_{j=1}^{n}\alpha_{j}\sum_{n=0}^{6}E_{n}^{\prime }\left(\xi_{j}x+\beta_{j}\right),$$

and

(42) $$\hat{y^{\prime \prime }}\left(x\right)=\sum_{j=1}^{n}\alpha_{j}\sum_{n=0}^{6}E_{n}^{\prime \prime }\left(\xi_{j}x+\beta_{j}\right),$$

The generic architecture for each problems of heat transfer can be formulated by using the appropriate network from Equations (40)–(42).

### 3.2. Objective Function

**Figure 4:**
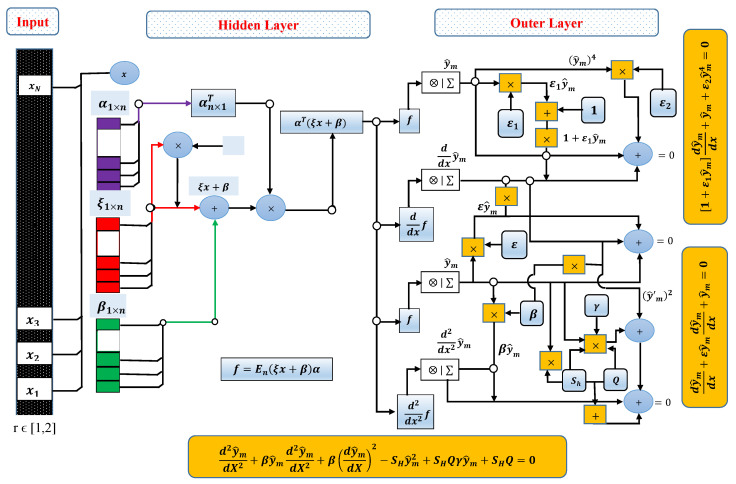
Euler polynomials based neural networks architecture for heat transfer and convective fin problems.

In order to find the neurons in ENN structure, objective function ζ is considered as a mean square errors which is formulated as

(43) $$\mathrm{Minimize}\;\zeta =\zeta_{1}+\zeta_{2},$$

where $\zeta_{1}$ is an error function for differential equations, while $\zeta_{2}$ represents the corresponding error function for boundary conditions. The elaborative form of Equations (6), (12), (26), and (32) is given as

(44) $$\mathrm{Minimize}\;\zeta =\frac{1}{M}\sum_{m=1}^{M}{\left(\left[1+\varepsilon_{1}{\hat{y}}_{m}\right]\frac{d{\hat{y}}_{m}}{dx}+{\hat{y}}_{m}+\varepsilon_{2}{\hat{y}}_{m}^{4}\right)}^{2}+{\left(\hat{y}\left(0\right)-1\right)}^{2},$$

(45) $$\mathrm{Minimize}\;\zeta =\frac{1}{M}\sum_{m=1}^{M}{\left(\frac{d{\hat{y}}_{m}}{dx}+\varepsilon {\hat{y}}_{m}\frac{d{\hat{y}}_{m}}{dx}+{\hat{y}}_{m}\right)}^{2}+{\left(\hat{y}\left(0\right)-1\right)}^{2},$$

(46) $$\begin{array}{c} \mathrm{Minimize}\;\zeta =\frac{1}{M}\sum_{m=1}^{M}{\left(\frac{d^{2}{\hat{y}}_{m}}{dX^{2}}+\beta {\hat{y}}_{m}\frac{d^{2}{\hat{y}}_{m}}{dX^{2}}+\beta {\left(\frac{d{\hat{y}}_{m}}{dX}\right)}^{2}-S_{h}{\hat{y}}_{m}^{2}+S_{h}Q\gamma {\hat{y}}_{m}+S_{h}Q\right)}^{2}\\ +\frac{1}{2}\left({\left(\hat{y}\left(1\right)-1\right)}^{2}+{\left(\hat{y^{\prime }}\left(0\right)-0\right)}^{2}\right), \end{array}$$

(47) $$\begin{array}{c} \mathrm{Minimize}\;\zeta =\frac{1}{M}\sum_{m=1}^{M}{\left(\frac{d^{2}{\hat{y}}_{m}}{dx^{2}}+\beta {\left(\frac{d{\hat{y}}_{m}}{dx}\right)}^{2}+\beta {\hat{y}}_{m}\frac{d^{2}{\hat{y}}_{m}}{dx^{2}}+\frac{\beta (\lambda -1)}{\left(1+(\lambda -1)x\right)}{\hat{y}}_{m}\frac{d{\hat{y}}_{m}}{dx}+\frac{\left(\lambda -1\right)}{\left(1+(\lambda -1)x\right)}\frac{d{\hat{y}}_{m}}{dx}-\frac{2Bi{\left(\lambda -1\right)}^{2}}{\delta }{\hat{y}}_{m}\right)}^{2}\\ +\frac{1}{2}\left({\left(\hat{y}\left(0\right)-1\right)}^{2}+{\left(\hat{y^{\prime }}\left(1\right)-0\right)}^{2}\right), \end{array}$$

where ${\hat{y}}_{m}=\hat{y}\left(x_{m}\right),x_{m}=mh,M=1/h$. The design of ENN architecture of the heat transfer and convection porous fin is shown through Figure 4.

### 3.3. Learning Procedure

In order to study the temperature distribution of heat transfer problems under the influence of specific heat, temperature-dependent thermal conductivity, and internal heat generation, corresponding optimization problems in terms of objective functions are formulated. Unknown neurons in ENN architecture are optimized by using a derivative-free technique called a generalized normal distribution optimization (GNDO) algorithm for global search and an interior-point algorithm (IPA) for local convergence of solutions. Figure 3 represents the working of GNDO and IPA. Details of both of the algorithms are given below.

#### 3.3.1. Generalized Normal Distribution Optimization Algorithm

The generalized normal distribution optimization (GNDO) algorithm is a novel meta-heuristic algorithm proposed by Yiying Zhang [71] inspired by the normal distribution model. In GNDO, the position of the individual is updated with the help of a normal distribution curve. The authors’ motivation for using GNDO algorithm is that it does not require any controlling parameters and any prior information about the problem. GNDO needs essential population size and specific terminal conditions before execution. The working procedure of the GNDO algorithm is divided into exploitation and exploration.

Exploitation:

During the process of exploitation, the algorithm searches to find better solutions around a search space that contains the current positions of all individuals. A model is developed for the relationship between normal distribution and individuals in populations which is given as

(48) $$a_{i}^{t}=\eta \times d_{i}+m_{i},\;i=1,2,3\ldots ,N,$$

where $a_{i}^{t}$ represents the trial vector of the ith individual, $\eta ,d_{i}$ and $m_{i}$ denote penalty factor, variance, and median, respectively, which are defined as

(49) $$\eta =\left\{\begin{array}{c} \sqrt{-\log\left(\lambda_{1}\right)}\times \cos\left(2\pi \lambda_{2}\right),\;\mathrm{if}\;a<=b.\\ \sqrt{-\log\left(\lambda_{1}\right)}\times \cos\left(2\pi \lambda_{2}+\pi \right),\;\mathrm{otherwise}\;, \end{array}\right.$$

(50) $$d_{i}=\sqrt{\frac{1}{3}\left[{\left(x_{Best}^{t}-\mu \right)}^{2}+{\left(x_{i}^{t}-\mu \right)}^{2}+{\left(M-\mu \right)}^{2}\right]},$$

(51) $$m_{i}=\frac{1}{3}\left(x_{i}^{t}+x_{Best}^{t}+M\right),$$

where $a,b,\lambda_{1}$ and $\lambda_{2}$ are random numbers between 0 and 1. M denotes generalized mean, which is given as

(52) $$M=\frac{\sum_{i=1}^{N}x_{i}^{t}}{N}.$$

The above discussed parameters are used to find that $x_{best}^{t}$ represents the current best position of the individual which is further modified by the process of exploration.

Exploitation:

Exploitation is a search of finding the global best solution in the entire population space. This process is based on three random variables which are expressed as

(53) $$a_{i}^{t}=x_{i}^{t}+\underset{\mathrm{Local}\;\mathrm{information}\;\mathrm{sharing}\;}{\underbrace{\beta \times \left(\left|\lambda_{3}\right|\times a_{1}\right)}}+\underset{\mathrm{Global}\;\mathrm{information}\;\mathrm{sharing}\;}{\underbrace{\left(1-\beta \right)\times \left(\left|\lambda_{4}\right|\times a_{2}\right)}}$$

where β is adjustment parameter, $\lambda_{3}$, $\lambda_{4}$ are random parameters subjecting to normal distribution are $a_{1},a_{2}$ are trail vectors, which are expressed as

(54) $$a_{1}=\left\{\begin{array}{c} x_{i}^{t}-x_{\mathrm{pl}}^{t},\;\mathrm{if}\;f\left(x_{i}^{t}\right)<f\left(x_{\mathrm{pl}}^{t}\right),\\ x_{p1}^{t}-x_{i}^{t},\;\mathrm{otherwise}\;, \end{array}\right.$$

(55) $$a_{2}=\left\{\begin{array}{c} x_{p2}^{t}-x_{p3}^{t},\;\mathrm{if}\;f\left(x_{p2}^{t}\right)<f\left(x_{p3}^{t}\right),\\ x_{p3}^{t}-x_{p2}^{t},\;\mathrm{otherwise}\;, \end{array}\right.$$

where p1, p2, and p3 are three random integers selected from 1 to N and p1≠p2≠p3≠i. Using the above procedure, GNDO gives the global best solution for the problem.

#### 3.3.2. Interior Point Algorithm

Interior point algorithm (IPA) is a local search technique used to fine-tune the unknown weights in ENN structure. IPA is a derivative-based technique that is derived from Lagrange multipliers [72]. It involves scaling function, maximum perturbations, and type of derivative. IPA is incorporated with the global best simulations of GNDO for optimization of heat transfer and convection porous fin problems in the hybridization process. Some recent applications of IP algorithm are numerical solutions for correction of array failure [73], simulation of viscoplastic fluid flows [74], and decentralized optimal power flow of multi-area interconnected power systems [75].

The procedural steps of the hybridized ENN-GNDO-IPA algorithm are presented graphically through Figure 3. The performance of the algorithm is dependent on parameter settings which are shown in Table 1. During the analysis of the proposed algorithm, it is observed that a slight change in these parameters results in premature convergence. Therefore, a lot of experience, care, and experimentations are needed for the selection of optimal parameters of the metaheuristic ENN-GNDO-IPA algorithm. A detailed explanation of the working procedure of the proposed technique is given in Algorithm 1.

**Algorithm** **1:** Pseudo code for hybridized ENN-GNDO-IPA algorithm. **Global Search Phase** **Generalized normal distribution optimizer: Start**
 **Input**:Population size N, The Upper and Lower bounds (u,l). Current number of iteration is t and maximum number of iterations is (Max_iter). Initial population is developed randomly by the entries of real number with number of dimensions equal to unknown parameters in ENN structure. Weights = **W** = [$\alpha_{j}$, $\xi_{j}$, $\beta_{j}$],   j=1,2,3…,n. **Population**:Generate population **P** of n candidates with the set of random weights drawn from a normal distribution as:P = [$C_{1}$, $C_{2}$, $C_{3}$, …, $C_{m}$]${}^{t}$,α = [$\alpha_{1}$, $\alpha_{2}$, $\alpha_{3}$, …, $\alpha_{n}$], ξ = [$\xi_{1}$, $\xi_{2}$, $\xi_{3}$, …, $\xi_{n}$] and β = [$\beta_{1}$, $\beta_{2}$, $\beta_{3}$, …, $\beta_{n}$]. **Output**: Choose the current best solution i.e., **$C_{GNGO_{Best}}$**. **Initializations of GNDO**: Initialize population **P**.
 **Fitness evaluation**:Evaluate the fitness value using Equations (6), (12), and (24) for each individual of population **C** in **P** and achieve the so far best solution $x_{Best}$.            The iteration is updated as t=t+1. **Main Loop**  **while**
(t≤(Max_iter))  **do**    **for** if  i=1:N       *p* is randomly generated between 0 and 1.       **if** p>0.5
       **Exploitation**Current best solution $x_{Best}$ is selected. **η**, ***d***, ***m*** and **M** are evaluated using Equations (42)–(45) to execute the process.       **else**
       **Exploration**The current best solution $x_{Best}$ is selected to perform exploration using Equations (46)–(48).       **end if**    **end for**         The iteration is updated as t=t+1.  **end while**
 **Termination**:Terminate the algorithm:Predefined number of iterations is achieved.Fitness $\epsilon \leq 10^{-20}$,TolFunc $\epsilon \leq 10^{-25}$ **Storage**: Store global best weights $C_{GNGO_{Best}}$ and corresponding fitness values. **Generalized normal distribution Optimization: End** **Local Search Phase** **Interior Point Algorithm: Start**
 **Inputs**:IPT is incorporated for fine tuning of parameter by taking the best weights of GNDO as the start point. **Output**: GNDO-IPA best weights i.e., $C_{GNGO-IPA}$ **Initialization**:   Start-Point as $C_{GNGO_{Best}}$ number of iterations, bound constraints. **Termination**:Adaption process ends if any of the following conditions are met:Fitness $\epsilon =10^{-20}$, total iterations ≤ 2000TolFun ≤$10^{-25}$, TolX ≤$10^{-25}$TolCon ≤$10^{-25}$, Max. Fun. Evaluations ≤ 200,000while (satisfied the required termination)**Fitness evaluation:** Calculate fitness of each weight vector **C**.**Fine-tuning:** Use ‘fmincon’ and ‘optimset’ routines of the MATLAB optimization toolbox for IPA. Update parameters of **C** for each generation of IPA and calculate fitness (ζ) of modified **C**. **Storage:** Accumulate weights vector $C_{GNDO-IPA}$, fitness value, iterations, and function evaluations. **Interior point Algorithm: End**
 **Data Generations**:Repeat 100 times the procedure steps to generate a massive data set of the optimization variables of ENN to solve heat transfer and convection porous fin problems.


## 4. Performance Indices

In this section, the performance of the design scheme for solving different problems of heat transfer is studied by incorporating performance indicators in terms of mean absolute error (MAE), Theil’s inequality coefficient (TIC), root mean square error (RMSE), Nash–Sutcliffe efficiency (NSE), and error in Nash–Sutcliffe efficiency (ENSE). Mathematical formulation of these indicators are given as [51].

(56) $$\mathrm{MAE}=\frac{1}{N}\sum_{m=1}^{N}\left|y_{m}\left(x\right)-{\hat{y}}_{m}x)\right|,$$

(57) $$\mathrm{TIC}=\frac{\sqrt{\frac{1}{N}\sum_{n=1}^{N}{\left(y_{m}\left(x\right)-{\hat{y}}_{m}\left(x\right)\right)}^{2}}}{\left(\sqrt{\frac{1}{N}\sum_{m=1}^{N}{\left(y_{m}\left(x\right)\right)}^{2}}+\sqrt{\frac{1}{N}\sum_{m=1}^{N}{\left({\hat{y}}_{m}\left(x\right)\right)}^{2}}\right)},$$

(58) $$\mathrm{RMSE}=\frac{1}{N}\sqrt{\sum_{m=1}^{N}{\left(y_{m}\left(x\right)-{\hat{y}}_{m}\left(x\right)\right)}^{2}},$$

(59) $$\;\mathrm{NSE}\;=\left\{1-\frac{\sum_{m=1}^{N}{\left(y_{m}\left(x\right)-{\hat{y}}_{m}\left(x\right)\right)}^{2}}{\sum_{m=1}^{N}{\left((y_{m}\left(x\right)-{\hat{y}}_{m}\left(x\right)\right)}^{2}},\;{\hat{y}}_{m}\left(x\right)=\frac{1}{N}\sum_{m=1}^{N}\hat{y}\left(x\right),\right.$$

(60) $$\;\mathrm{ENSE}\;=\left(1-\mathrm{NSE}\right).$$

where $y_{m}$ is analytical solution and ${\hat{y}}_{m}$ represents the approximate solution by the proposed algorithm; *N* also denotes the grid points.

## 5. Numerical Experimentation and Discussion

**Figure 5:**
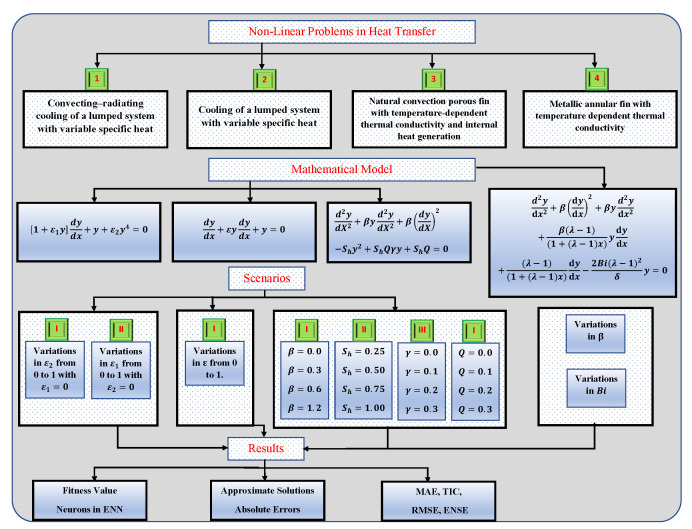
Graphical overview of problems along with different cases studied in this paper.

To evaluate the proposed method, problems with different scenarios are considered as shown in Figure 5.

Problems 1 to 4 are given as follows:

Problem 1: Convecting-radiating cooling of a lumped system with variable specific heat.

In this problem, nonlinear Equation (6) along with initial condition Equation (7) are considered to study the temperature distribution under the effect of variations in specific heat. An unsupervised fitness function for governing equation of the model is described below:

(61) $$\mathrm{Minimize}\;\zeta =\frac{1}{M}\sum_{m=1}^{M}{\left(\left[1+\varepsilon_{1}{\hat{y}}_{m}\right]\frac{d{\hat{y}}_{m}}{dx}+{\hat{y}}_{m}+\varepsilon_{2}{\hat{y}}_{m}^{4}\right)}^{2}+{\left(\hat{y}\left(0\right)-1\right)}^{2},$$

To briefly study the model, two scenarios are considered. In scenario-I, we assume that $\varepsilon_{1}=0$, and $\varepsilon_{2}$ varies from 0 to 1 with a step size of 0.1. In addition, in scenario-II, $\varepsilon_{2}$ is assumed to be 1, and $\varepsilon_{1}$ varies from 0 to 1 with step size 0.1.

**Figure 6:**
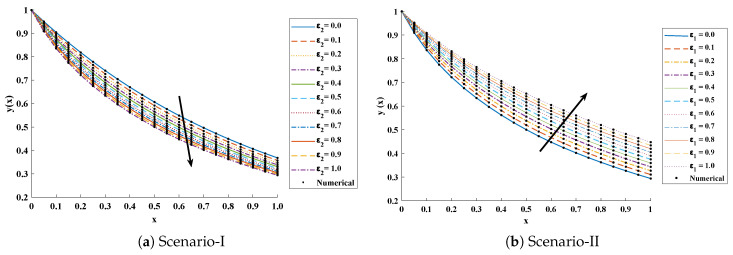
Comparison of approximate solutions obtained by proposed algorithm with a numerical solver (Ode45) for different cases of scenarios-I and II of problem 1.

**Figure 7:**
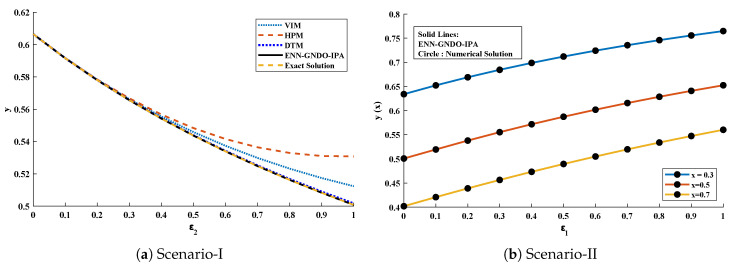
Comparison of approximate solutions obtained by the proposed algorithm with existing techniques in literature to study the influence of variations in specific heat $\varepsilon_{2}$ at x=0.5 and $\varepsilon_{1}$ at x=0.3,0.5 and 0.7.

**Figure 8:**
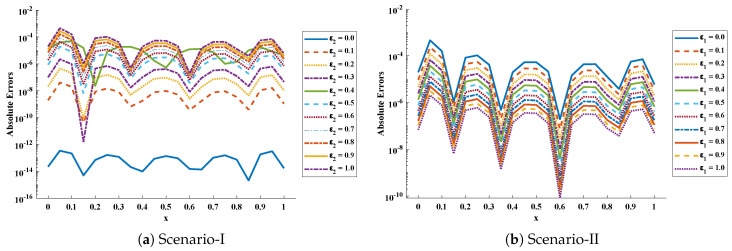
Graphical representation of absolute errors in solutions obtained by ENN-GNDO-IPA algorithm for scenario-I and II of problem 1.

**Figure 9:**
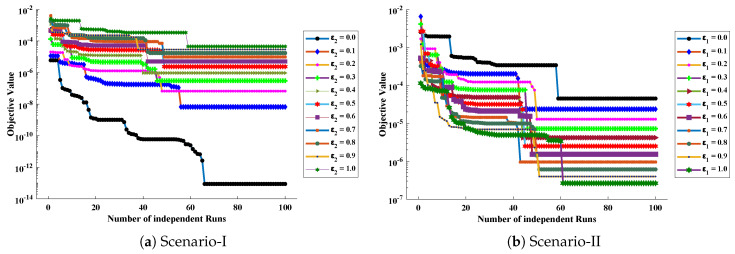
Convergence of objective value during 100 independent executions of the proposed technique for Scenario-I and Scenario-II of problem 1.

**Figure 10:**
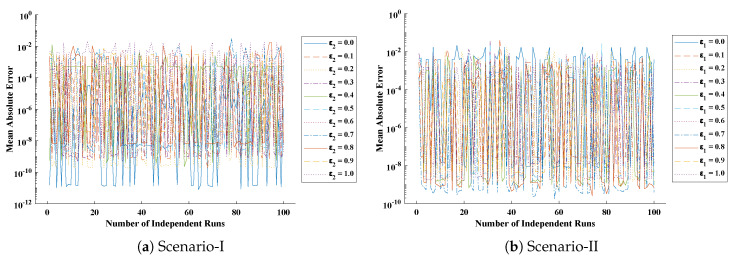
Convergence of Mean absolute errors (MAE) during 100 independent executions of proposed technique for Scenario-I and Scenario-II of problem 1.

**Figure 11:**
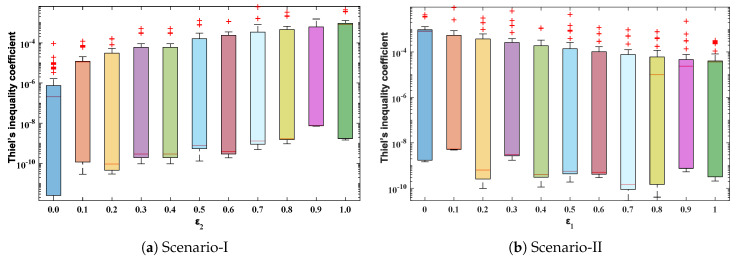
Boxplots for TIC of Scenario-I and Scenario-II of problem 1 to study the performance of the proposed algorithm.

**Figure 12:**
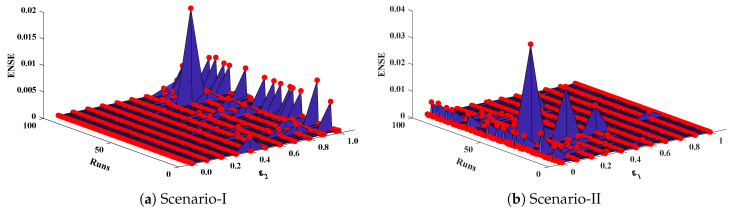
Graphical illustration of ENSE for problem 1.

**Figure 13:**
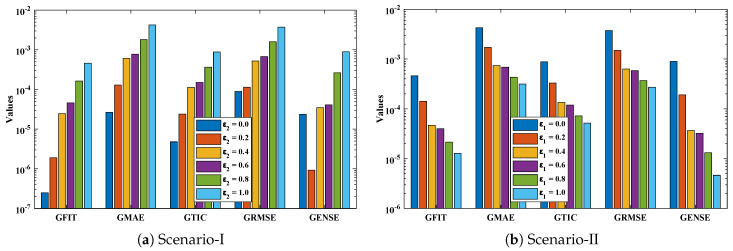
Analysis on global values of fitness function, MAE, TIC, RMSE, and ENSE.

**Figure 14:**
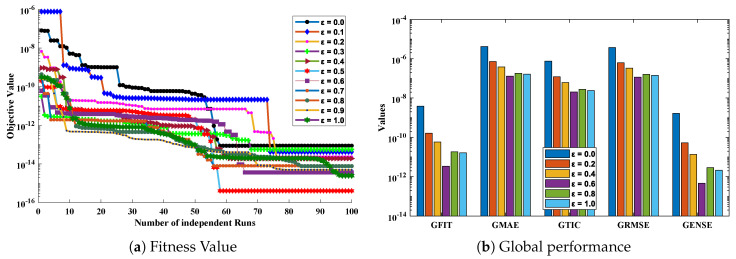
(**a**) Convergence of fitness function during the process of optimization by ENN-GNDO-IPA algorithm for variations in ε of cooling lumped system along with (**b**) mean values of fitness function, MAE, TIC, RMSE, and ENSE obtained during 100 runs.

The objective function Equation (61) for scenario I and scenario II has been optimized by executing the proposed algorithm for 100 independent runs. Approximate solutions for the influence of variations in specific heat $\varepsilon_{2}$ and $\varepsilon_{1}$ on temperature distributions are disclosed in Table 2 and Table 3, respectively. Figure 6 shows comparison of our solutions with numerical solver RK-4 (ode45). It can be seen that temperature distribution varies directly with an increase in $\varepsilon_{2}$, while it varies inversely with variations in $\varepsilon_{1}$. Furthermore, to validate the results, we have compared the solutions with VIM, HPM, DTM, and exact solutions as shown in Figure 7. The calculated values of absolute errors (AE) in Table 4 and Table 5 show the accuracy of solutions obtained by the ENN-GNDO-IPA algorithm. The values of AE for different scenarios lie around $10^{-4}$ to $10^{-14}$ and $10^{-4}$ to $10^{-9}$ as shown in Figure 8. Statistics on minimum and mean value of errors with step size 0.05 are dictated in Table 6 and Table 7, respectively. The convergence of objective function for both scenarios is shown in Figure 9. Graphics of performance indicators including MAE, TIC, and ENSE during 100 trails along with their global values are shown through Figure 10, Figure 11 and Figure 12, respectively. Furthermore, global performance indicators are depicted in Figure 13 and Figure 14, respectively.

The detailed evaluation of fitness function and performance measures is carried out by statistical performance in terms of minimum, mean, and standard deviation as shown in Table 8 and Table 9, and best so far design weights for solutions of different scenarios are given in Table 10. It can be seen that values of fitness function and performance measures lie around $10^{-5}$ to $10^{-14}$, $10^{-8}$ to $10^{-12}$, $10^{-9}$ to $10^{-12}$, $10^{-8}$ to $10^{-12}$ and 0 to $10^{-16}$, respectively.

Approximate series solution obtained by the proposed technique for Scenario-I ($\varepsilon_{2}=0.0,0.2,0.4,0.6,0.8$ and 1.0) of problem 1.

(62) $$y=\left\{\begin{array}{c} -0.32346+0.27153\left(\left(0.832097x+4.019612\right)-\frac{1}{2}\right)\\ 2.246418\left({\left(1.038388x+0.979799\right)}^{2}-\left(1.038388x+0.979799\right)\right)\\ -0.37954\left({\left(1.306827x+2.666845\right)}^{3}-\frac{3}{2}{\left(1.306827x+2.666845\right)}^{2}+\frac{1}{4}\right)\\ 3.004562\left({\left(0.323587x+1.392797\right)}^{4}-2{\left(0.323587x+1.392797\right)}^{3}+\left(0.323587x+1.392797\right)\right)\\ -0.57037\left({\left(-0.2145x-1.32254\right)}^{3}-\frac{5}{2}{\left(-0.2145x-1.32254\right)}^{4}+\frac{5}{2}{\left(-0.2145x-1.32254\right)}^{2}-\frac{1}{2}\right)\\ 0.979534\left({\left(-0.30895x-4.23323\right)}^{6}-3{\left(-0.30895x-4.23323\right)}^{5}+5{\left(-0.30895x-4.23323\right)}^{3}-3\left(-0.30895x-4.23323\right)\right) \end{array}\right.$$

(63) $$y=\left\{\begin{array}{c} -1.60825+3.022705\left(\left(-3.46811x-0.55204\right)-\frac{1}{2}\right)\\ 2.062048\left({\left(0.485282x+0.89244\right)}^{2}-\left(0.485282x+0.89244\right)\right)\\ 7.494845\left({\left(-0.94149x+0.140744\right)}^{3}-\frac{3}{2}{\left(-0.94149x+0.140744\right)}^{2}+\frac{1}{4}\right)\\ -7.35009\left({\left(-0.56594x-1.2552\right)}^{4}-2{\left(-0.56594x-1.2552\right)}^{3}+\left(-0.56594x-1.2552\right)\right)\\ -12.8649\left({\left(-0.3453x-1.13346\right)}^{3}-\frac{5}{2}{\left(-0.3453x-1.13346\right)}^{4}+\frac{5}{2}{\left(-0.3453x-1.13346\right)}^{2}-\frac{1}{2}\right)\\ 1.044753\left({\left(-0.69791x-19.8302\right)}^{6}-3{\left(-0.69791x-19.8302\right)}^{5}+5{\left(-0.69791x-19.8302\right)}^{3}-3\left(-0.69791x-19.8302\right)\right) \end{array}\right.$$

(64) $$y=\left\{\begin{array}{c} 0.538667+0.032045\left(\left(2.283121x-5.06232\right)-\frac{1}{2}\right)\\ 3.393367\left({\left(0.024783x-0.1751\right)}^{2}-\left(0.024783x-0.1751\right)\right)\\ -0.0906\left({\left(1.07681x-0.50635\right)}^{3}-\frac{3}{2}{\left(1.07681x-0.50635\right)}^{2}+\frac{1}{4}\right)\\ 0.384711\left({\left(0.238527x-0.99735\right)}^{4}-2{\left(0.238527x-0.99735\right)}^{3}+\left(0.238527x-0.99735\right)\right)\\ -0.00036\left({\left(3.964444x-2.24627\right)}^{3}-\frac{5}{2}{\left(3.964444x-2.24627\right)}^{4}+\frac{5}{2}{\left(3.964444x-2.24627\right)}^{2}-\frac{1}{2}\right)\\ -0.80607\left({\left(0.013279x+2.390564\right)}^{6}-3{\left(0.013279x+2.390564\right)}^{5}+5{\left(0.013279x+2.390564\right)}^{3}-3\left(0.013279x+2.390564\right)\right) \end{array}\right.$$

(65) $$y=\left\{\begin{array}{c} -16.6655-4.67217\left(\left(4.563917x-7.79808\right)-\frac{1}{2}\right)\\ -5.85542\left({\left(-1.71428x-3.87456\right)}^{2}-\left(-1.71428x-3.87456\right)\right)\\ 14.32105\left({\left(-1.27367x-0.31084\right)}^{3}-\frac{3}{2}{\left(-1.27367x-0.31084\right)}^{2}+\frac{1}{4}\right)\\ -13.4506\left({\left(-0.34559x-1.43197\right)}^{4}-2{\left(-0.34559x-1.43197\right)}^{3}+\left(-0.34559x-1.43197\right)\right)\\ 0.059118\left({\left(1.175466x+5.699086\right)}^{3}-\frac{5}{2}{\left(1.175466x+5.699086\right)}^{4}+\frac{5}{2}{\left(1.175466x+5.699086\right)}^{2}-\frac{1}{2}\right)\\ 1.125914\left({\left(-0.93365x+14.77236\right)}^{6}-3{\left(-0.93365x+14.77236\right)}^{5}+5{\left(-0.93365x+14.77236\right)}^{3}-3\left(-0.93365x+14.77236\right)\right) \end{array}\right.$$

(66) $$y=\left\{\begin{array}{c} 5.398497+2.033893\left(\left(-16.973x-19.9974\right)-\frac{1}{2}\right)\\ -6.77248\left({\left(-0.18372x+3.28685\right)}^{2}-\left(-0.18372x+3.28685\right)\right)\\ -14.8902\left({\left(-1.27367x-0.31084\right)}^{3}-\frac{3}{2}{\left(-1.27367x-0.31084\right)}^{2}+\frac{1}{4}\right)\\ 0.015304\left({\left(-0.58787x-18.9454\right)}^{4}-2{\left(-0.58787x-18.9454\right)}^{3}+\left(-0.58787x-18.9454\right)\right)\\ -4.45923\left({\left(0.806267x-0.36351\right)}^{3}-\frac{5}{2}{\left(0.806267x-0.36351\right)}^{4}+\frac{5}{2}{\left(0.806267x-0.36351\right)}^{2}-\frac{1}{2}\right)\\ 0.946263\left({\left(0.946263x-8.81925\right)}^{6}-3{\left(0.946263x-8.81925\right)}^{5}+5{\left(0.946263x-8.81925\right)}^{3}-3\left(0.946263x-8.81925\right)\right) \end{array}\right.$$

(67) $$y=\left\{\begin{array}{c} 4.975563-4.38429\left(\left(-4.51117x-0.44905\right)-\frac{1}{2}\right)\\ -2.11292\left({\left(4.103217x+2.82157\right)}^{2}-\left(4.103217x+2.82157\right)\right)\\ -4.94373\left({\left(2.502122x+0.985106\right)}^{3}-\frac{3}{2}{\left(2.502122x+0.985106\right)}^{2}+\frac{1}{4}\right)\\ 4.999435\left({\left(-0.68521x+1.465666\right)}^{4}-2{\left(-0.68521x+1.465666\right)}^{3}+\left(-0.68521x+1.465666\right)\right)\\ -3.08032\left({\left(1.507781x+1.104728\right)}^{3}-\frac{5}{2}{\left(1.507781x+1.104728\right)}^{4}+\frac{5}{2}{\left(1.507781x+1.104728\right)}^{2}-\frac{1}{2}\right)\\ 2.001408\left({\left(0.988829x+4.187431\right)}^{6}-3{\left(0.988829x+4.187431\right)}^{5}+5{\left(0.988829x+4.187431\right)}^{3}-3\left(0.988829x+4.187431\right)\right) \end{array}\right.$$

Approximate series solution obtained by the proposed technique for Scenario-II ($\varepsilon_{1}=0.2,0.4,0.6,0.8$ and 1.0) of problem 1.

(68) $$y=\left\{\begin{array}{c} -12.9405-2.92286\left(\left(-16.4296x-2.97641\right)-\frac{1}{2}\right)\\ -0.66817\left({\left(5.729773x+3.180745\right)}^{2}-\left(5.729773x+3.180745\right)\right)\\ -2.57307\left({\left(-0.58965x+1.647936\right)}^{3}-\frac{3}{2}{\left(-0.58965x+1.647936\right)}^{2}+\frac{1}{4}\right)\\ 0.512251\left({\left(1.497244x+0.544689\right)}^{4}-2{\left(1.497244x+0.544689\right)}^{3}+\left(1.497244x+0.544689\right)\right)\\ 21.14455\left({\left(-0.31939x+1.852215\right)}^{3}-\frac{5}{2}{\left(-0.31939x+1.852215\right)}^{4}+\frac{5}{2}{\left(-0.31939x+1.852215\right)}^{2}-\frac{1}{2}\right)\\ 1.111514\left({\left(-0.98518x+3.874896\right)}^{6}-3{\left(-0.98518x+3.874896\right)}^{5}+5{\left(-0.98518x+3.874896\right)}^{3}-3\left(-0.98518x+3.874896\right)\right) \end{array}\right.$$

(69) $$y=\left\{\begin{array}{c} -11.6996-0.03635\left(\left(-4.84933x-11.9036\right)-\frac{1}{2}\right)\\ -1.04845\left({\left(0.124293x+4.954715\right)}^{2}-\left(0.124293x+4.954715\right)\right)\\ -1.64457\left({\left(1.807524x+3.868266\right)}^{3}-\frac{3}{2}{\left(1.807524x+3.868266\right)}^{2}+\frac{1}{4}\right)\\ 0.795072\left({\left(1.193228x-0.83318\right)}^{4}-2{\left(1.193228x-0.83318\right)}^{3}+\left(1.193228x-0.83318\right)\right)\\ 2.7803\left({\left(0.418961x+2.748523\right)}^{3}-\frac{5}{2}{\left(0.418961x+2.748523\right)}^{4}+\frac{5}{2}{\left(0.418961x+2.748523\right)}^{2}-\frac{1}{2}\right)\\ 1.090368\left({\left(-0.90066x+11.85451\right)}^{6}-3{\left(-0.90066x+11.85451\right)}^{5}+5{\left(-0.90066x+11.85451\right)}^{3}-3\left(-0.90066x+11.85451\right)\right) \end{array}\right.$$

(70) $$y=\left\{\begin{array}{c} 18.3322-1.20137\left(\left(7.701688x+9.668631\right)-\frac{1}{2}\right)\\ 0.406851\left({\left(-14.6591x-14.557\right)}^{2}-\left(-14.6591x-14.557\right)\right)\\ 2.716671\left({\left(-2.44506x-3.20313\right)}^{3}-\frac{3}{2}{\left(-2.44506x-3.20313\right)}^{2}+\frac{1}{4}\right)\\ 6.86276\left({\left(1.259748x+2.392652\right)}^{4}-2{\left(1.259748x+2.392652\right)}^{3}+\left(1.259748x+2.392652\right)\right)\\ -16.8332\left({\left(0.589292x+1.975657\right)}^{3}-\frac{5}{2}{\left(0.589292x+1.975657\right)}^{4}+\frac{5}{2}{\left(0.589292x+1.975657\right)}^{2}-\frac{1}{2}\right)\\ 1.01E-05\left({\left(5.705381x-24.8603\right)}^{6}-3{\left(5.705381x-24.8603\right)}^{5}+5{\left(5.705381x-24.8603\right)}^{3}-3\left(5.705381x-24.8603\right)\right) \end{array}\right.$$

(71) $$y=\left\{\begin{array}{c} 5.24664+0.764505\left(\left(9.131144x+7.922359\right)-\frac{1}{2}\right)\\ 1.602824\left({\left(-1.62948x-2.97812\right)}^{2}-\left(-1.62948x-2.97812\right)\right)\\ 0.372859\left({\left(-2.86218x+0.049793\right)}^{3}-\frac{3}{2}{\left(-2.86218x+0.049793\right)}^{2}+\frac{1}{4}\right)\\ -4.95236\left({\left(0.491655x+2.428753\right)}^{4}-2{\left(0.491655x+2.428753\right)}^{3}+\left(0.491655x+2.428753\right)\right)\\ 4.313692\left({\left(0.450455x+2.120649\right)}^{3}-\frac{5}{2}{\left(0.450455x+2.120649\right)}^{4}+\frac{5}{2}{\left(0.450455x+2.120649\right)}^{2}-\frac{1}{2}\right)\\ 1.063243\left({\left(-0.76796x-6.85092\right)}^{6}-3{\left(-0.76796x-6.85092\right)}^{5}+5{\left(-0.76796x-6.85092\right)}^{3}-3\left(-0.76796x-6.85092\right)\right) \end{array}\right.$$

(72) $$y=\left\{\begin{array}{c} 21.83495-5.6752\left(\left(6.475667x+10.7567\right)-\frac{1}{2}\right)\\ 4.946748\left({\left(3.055212x+5.268929\right)}^{2}-\left(3.055212x+5.268929\right))\right)\\ -0.10106\left({\left(6.522438x+11.53309\right)}^{3}-\frac{3}{2}{\left(6.522438x+11.53309\right)}^{2}+\frac{1}{4}\right)\\ 2.802585\left({\left(-1.16325x-1.88799\right)}^{4}-2{\left(-1.16325x-1.88799\right)}^{3}+\left(-1.16325x-1.88799\right)\right)\\ 0.186006\left({\left(-1.17559x-1.80289\right)}^{3}-\frac{5}{2}{\left(-1.17559x-1.80289\right)}^{4}+\frac{5}{2}{\left(-1.17559x-1.80289\right)}^{2}-\frac{1}{2}\right)\\ 0.000464\left({\left(2.592857x-19.0434\right)}^{6}-3{\left(2.592857x-19.0434\right)}^{5}+5{\left(2.592857x-19.0434\right)}^{3}-3\left(2.592857x-19.0434\right)\right) \end{array}\right.$$

Problem 2: Cooling of a lumped system with variable specific heat.

In this problem, Equations (12) and (13) are considered to study the influence of variations in ε on the cooling of a lumped system. In order to investigate the model, an objective function is constructed

(73) $$\mathrm{Minimize}\;\zeta =\frac{1}{M}\sum_{m=1}^{M}{\left(\frac{d{\hat{y}}_{m}}{dx}+\varepsilon {\hat{y}}_{m}\frac{d{\hat{y}}_{m}}{dx}+{\hat{y}}_{m}\right)}^{2}+{\left(\hat{y}\left(0\right)-1\right)}^{2},$$

**Figure 15:**
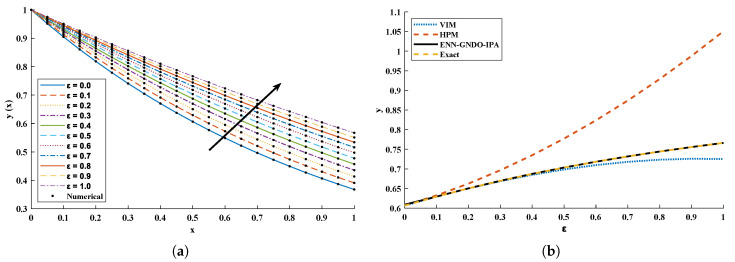
(**a**) Comparison of approximate solutions obtained by the proposed algorithm with a numerical solver (Ode45) for different cases; (**b**) comparison of a design scheme with a state-of-the-art algorithm, to study the influence of specific heat on temperature distribution at x=0.5.

In this problem, ε is varied from 0 to 1 with a step size of 0.1. The optimization of fitness function Equation (73) is carried out executing a proposed algorithm for 100 independent trails. The convergence plot of the fitness function is shown in Figure 14, which shows that the design scheme converges for all cases of the cooling lumped system. To access the level of accuracy, approximate solutions and absolute errors are calculated as shown in Table 11 and Table 12, respectively. Furthermore, to extend the correctness of the design scheme, Figure 15 is plotted that shows the comparison of results with state-of-the-art techniques including VIM, DTM, and exact solution along with numerical solver RK-4 (ode45). The magnitude of absolute errors for each case lies around $1.04\times 10^{-13}$ to $9.78\times 10^{-15}$, $3.01\times 10^{-14}$ to $8.92\times 10^{-15}$, $1.35\times 10^{-13}$ to $1.50\times 10^{-15}$, $1.25\times 10^{-13}$ to $6.98\times 10^{-15}$, $1.84\times 10^{-14}$ to $2.10\times 10^{-15}$, $1.42\times 10^{-15}$ to $3.07\times 10^{-17}$, $9.24\times 10^{-15}$ to $8.93\times 10^{-17}$, $1.84\times 10^{-14}$ to $5.45\times 10^{-16}$, $1.81\times 10^{-14}$ to $6.83\times 10^{-16}$, $1.05\times 10^{-14}$ to $9.59\times 10^{-16}$ and $2.00\times 10^{-15}$ to $6.37\times 10^{-16}$. From Table 13, it can be noticed that the proposed technique approaches the exact solution with mean errors that lie around $10^{-9}$ to $10^{-13}$.

**Figure 16:**
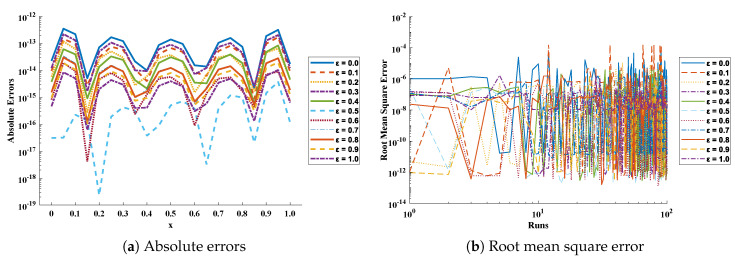
(**a**) Absolute errors obtained in our solutions for different cases of problem 2; (**b**) values of root mean square error obtained during 100 independent executions of ENN-GNDO-IPA for cooling of lumped system with variable specific heat.

**Figure 17:**
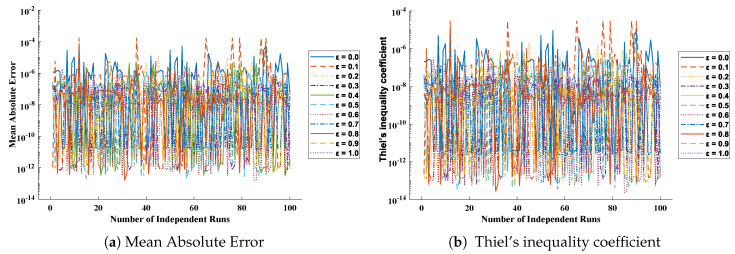
Graphical view of the behavior of performance measures including MAE and TIC for variation in specific heat in problem 2.

**Figure 18:**
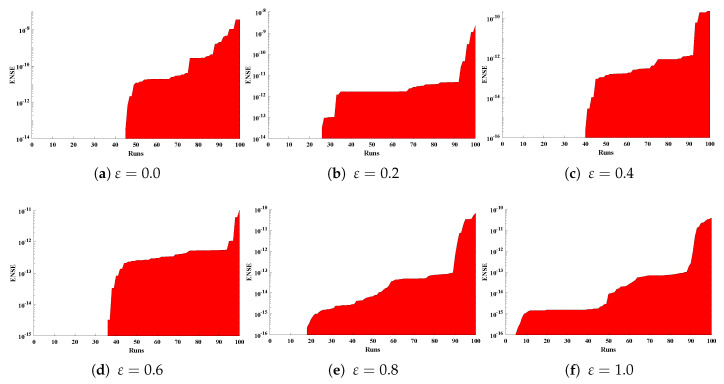
Convergence of ENSE for 100 independent executions of the proposed algorithm for different variations in specific heat (ε) in the case of problem 2.

**Figure 19:**
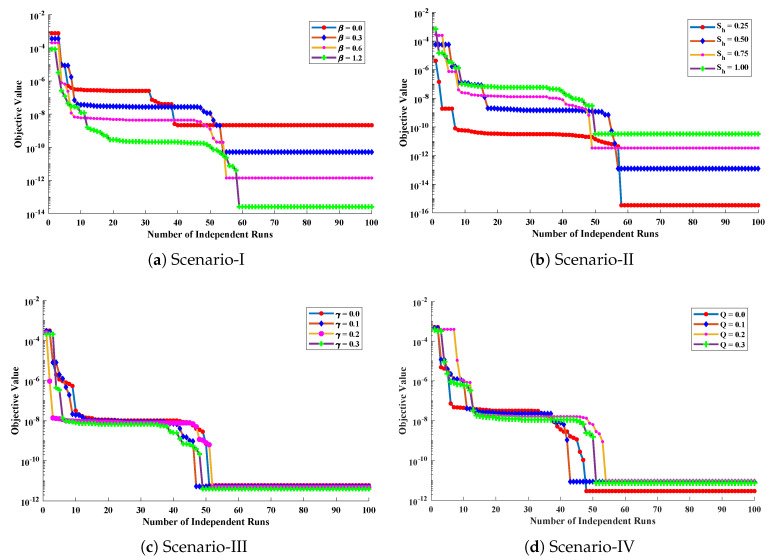
Convergence of fitness value during 100 independent executions of the proposed algorithm for studying the influence of different variations in temperature distribution of convective porous fin.

The values of performance indices measuring the objective value, mean absolute error, Theil’s inequality coefficient, root mean square error, and error in Nash–Sutcliffe efficiency are tabulated in Table 14, representing the minimum values, mean values, and standard deviations. Convergence of RMSE, MAE, TIC, and ENSE during 100 independent trials are plotted through Figure 16, Figure 17, Figure 18 and Figure 19. Unknown neurons in ENN structure are given in Table 15 that are used to obtain the approximate solution for a cooling lumped system with variable specific heat.

(74) $$y=\left\{\begin{array}{c} -4.39897-0.92207\left(\left(-3.09075x-2.9852\right)-\frac{1}{2}\right)\\ 0.105321\left({\left(4.692673x-1.48857\right)}^{2}-\left(4.692673x-1.48857\right))\right)\\ -4.04789\left({\left(-0.59271x-2.58833\right)}^{3}-\frac{3}{2}{\left(-0.59271x-2.58833\right)}^{2}+\frac{1}{4}\right)\\ -0.32131\left({\left(-0.82962x-4.85856\right)}^{4}-2{\left(-0.82962x-4.85856\right)}^{3}+\left(-0.82962x-4.85856\right)\right)\\ 0.729003\left({\left(0.349116x+3.543819\right)}^{3}-\frac{5}{2}{\left(0.349116x+3.543819\right)}^{4}+\frac{5}{2}{\left(0.349116x+3.543819\right)}^{2}-\frac{1}{2}\right)\\ 1.172034\left({\left(-0.29985x+0.866929\right)}^{6}-3{\left(-0.29985x+0.866929\right)}^{5}+5{\left(-0.29985x+0.866929\right)}^{3}-3\left(-0.29985x+0.866929\right)\right) \end{array}\right.$$

(75) $$y=\left\{\begin{array}{c} -1.49844+0.688986\left(\left(1.197062x-1.75214\right)-\frac{1}{2}\right)\\ 1.073087\left({\left(0.513938x-0.75682\right)}^{2}-\left(0.513938x-0.75682\right))\right)\\ 1.090927\left({\left(-0.26679x+3.20621\right)}^{3}-\frac{3}{2}{\left(-0.26679x+3.20621\right)}^{2}+\frac{1}{4}\right)\\ -0.08806\left({\left(0.300433x-3.30992\right)}^{4}-2{\left(0.300433x-3.30992\right)}^{3}+\left(0.300433x-3.30992\right)\right)\\ 0.762931\left({\left(0.312458x+0.398631\right)}^{3}-\frac{5}{2}{\left(0.312458x+0.398631\right)}^{4}+\frac{5}{2}{\left(0.312458x+0.398631\right)}^{2}-\frac{1}{2}\right)\\ -1.65E-08\left({\left(4.999639x+2.280151\right)}^{6}-3{\left(4.999639x+2.280151\right)}^{5}+5{\left(4.999639x+2.280151\right)}^{3}-3\left(4.999639x+2.280151\right)\right) \end{array}\right.$$

(76) $$y=\left\{\begin{array}{c} 1.089725+0.157584\left(\left(-4.99808x+0.252828\right)-\frac{1}{2}\right)\\ 0.722313\left({\left(-0.79927x-0.50548\right)}^{2}-\left(-0.79927x-0.50548\right))\right)\\ 0.021526\left({\left(0.646512x-0.56532\right)}^{3}-\frac{3}{2}{\left(0.646512x-0.56532\right)}^{2}+\frac{1}{4}\right)\\ 2.510246\left({\left(0.282213x+1.040526\right)}^{4}-2{\left(0.282213x+1.040526\right)}^{3}+\left(0.282213x+1.040526\right)\right)\\ -0.00603\left({\left(0.677488x+2.970961\right)}^{3}-\frac{5}{2}{\left(0.677488x+2.970961\right)}^{4}+\frac{5}{2}{\left(0.677488x+2.970961\right)}^{2}-\frac{1}{2}\right)\\ 0.224675\left({\left(-0.30731x+1.134809\right)}^{6}-3{\left(-0.30731x+1.134809\right)}^{5}+5{\left(-0.30731x+1.134809\right)}^{3}-3\left(-0.30731x+1.134809\right)\right) \end{array}\right.$$

(77) $$y=\left\{\begin{array}{c} -0.44799-0.13267\left(\left(0.788568x-1.22388\right)-\frac{1}{2}\right)\\ 0.279031\left({\left(0.176915x+0.648846\right)}^{2}-\left(0.176915x+0.648846\right))\right)\\ -0.31118\left({\left(0.412364x-0.63643\right)}^{3}-\frac{3}{2}{\left(0.412364x-0.63643\right)}^{2}+\frac{1}{4}\right)\\ -0.44781\left({\left(0.287221x-0.05257\right)}^{4}-2{\left(0.287221x-0.05257\right)}^{3}+\left(0.287221x-0.05257\right)\right)\\ 2.136315\left({\left(0.101306x+1.005993\right)}^{3}-\frac{5}{2}{\left(0.101306x+1.005993\right)}^{4}+\frac{5}{2}{\left(0.101306x+1.005993\right)}^{2}-\frac{1}{2}\right)\\ 0.000511\left({\left(0.816928x+3.968431\right)}^{6}-3{\left(0.816928x+3.968431\right)}^{5}+5{\left(0.816928x+3.968431\right)}^{3}-3\left(0.816928x+3.968431\right)\right) \end{array}\right.$$

(78) $$y=\left\{\begin{array}{c} 1.057026+0.367921\left(\left(-0.10381x-2.15276\right)-\frac{1}{2}\right)\\ 0.507991\left({\left(0.469834x-0.93386\right)}^{2}-\left(0.469834x-0.93386\right))\right)\\ -0.31011\left({\left(-0.47249x+0.878305\right)}^{3}-\frac{3}{2}{\left(-0.47249x+0.878305\right)}^{2}+\frac{1}{4}\right)\\ -0.06205\left({\left(-0.64363x+1.012157\right)}^{4}-2{\left(-0.64363x+1.012157\right)}^{3}+\left(-0.64363x+1.012157\right)\right)\\ 0.360674\left({\left(-0.29154x+1.395485\right)}^{3}-\frac{5}{2}{\left(-0.29154x+1.395485\right)}^{4}+\frac{5}{2}{\left(-0.29154x+1.395485\right)}^{2}-\frac{1}{2}\right)\\ 0.221282\left({\left(-0.2437x-0.83885\right)}^{6}-3{\left(-0.2437x-0.83885\right)}^{5}+5{\left(-0.2437x-0.83885\right)}^{3}-3\left(-0.2437x-0.83885\right)\right) \end{array}\right.$$

(79) $$y=\left\{\begin{array}{c} 0.435104+-0.31606\left(\left(0.563393x-0.54045\right)-\frac{1}{2}\right)\\ -1.18391\left({\left(-0.23838x+0.677937\right)}^{2}-\left(-0.23838x+0.677937\right))\right)\\ -0.05796\left({\left(-0.52657x+0.154324\right)}^{3}-\frac{3}{2}{\left(-0.52657x+0.154324\right)}^{2}+\frac{1}{4}\right)\\ 0.707663\left({\left(0.146812x-0.66243\right)}^{4}-2{\left(0.146812x-0.66243\right)}^{3}+\left(0.146812x-0.66243\right)\right)\\ -0.09903\left({\left(0.22877x+0.301598\right)}^{3}-\frac{5}{2}{\left(0.22877x+0.301598\right)}^{4}+\frac{5}{2}{\left(0.22877x+0.301598\right)}^{2}-\frac{1}{2}\right)\\ -0.20501\left({\left(-0.18528x-0.96816\right)}^{6}-3{\left(-0.18528x-0.96816\right)}^{5}+5{\left(-0.18528x-0.96816\right)}^{3}-3\left(-0.18528x-0.96816\right)\right) \end{array}\right.$$

Problem 3: Natural convection porous fin with temperature-dependent thermal conductivity and internal heat generation.

In this problem, a mathematical model of convection porous fin with temperature-dependent thermal conductivity and internal heat generation is considered as presented by Equations (26) and (27). The ENN-GNDO-IPA algorithm is applied to study the temperature distribution of convective porous fin under the influence of variation in β, $S_{h}$, γ, and *Q*. An unsupervised objective function in terms of mean square errors is given as

(80) $$\begin{array}{c} \mathrm{Minimize}\;\zeta =\frac{1}{M}\sum_{m=1}^{M}{\left(\frac{d^{2}{\hat{y}}_{m}}{dX^{2}}+\beta {\hat{y}}_{m}\frac{d^{2}{\hat{y}}_{m}}{dX^{2}}+\beta {\left(\frac{d{\hat{y}}_{m}}{dX}\right)}^{2}-S_{h}{\hat{y}}_{m}^{2}+S_{h}Q\gamma {\hat{y}}_{m}+S_{h}Q\right)}^{2}\\ +\frac{1}{2}\left({\left(\hat{y}\left(1\right)-1\right)}^{2}+{\left(\hat{y^{\prime }}\left(0\right)-0\right)}^{2}\right), \end{array}$$

Furthermore, to study the model extensively, we have considered four scenarios as follows.

In scenario-I, the effect of variation in β has been studied i.e., β=0.0,0.3,0.6 and 1.2 with Q=0.4, γ=0.2 and $S_{h}=1.0$. In scenario-II, $S_{h}$ has been varied i.e., $S_{h}=0.25,0.50,0.75$ and 1.00 with Q=γ=0 and β=0.4. In scenario-III, temperature distribution has been investigated with variation in γ i.e., γ=0.0,0.1,0.2 and 0.3 with β=0.5, Q=0.4 and $S_{h}=1.0$, and, in scenario-IV, the influence of variations in *Q* has been investigated i.e., Q=0.0,0.1,0.2 and 0.3 with β=0.5, γ=0.2, and $S_{h}=1.0$.

**Figure 20:**
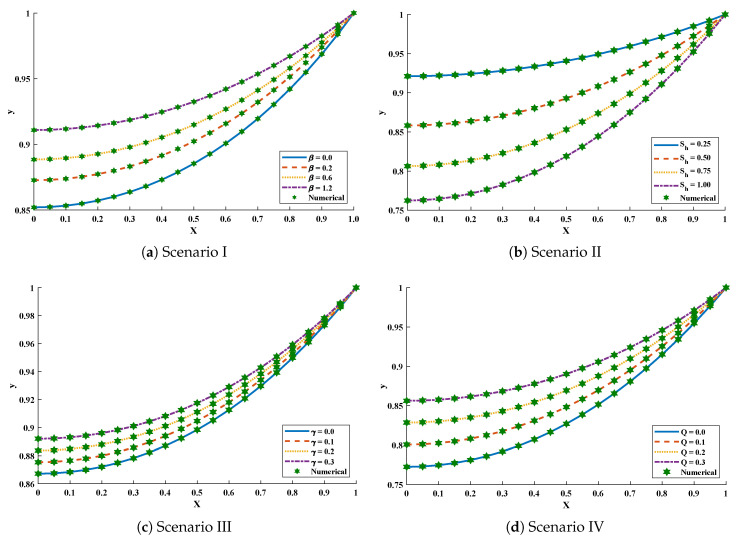
Comparison of approximate solutions by the proposed algorithm with numerical solver RK-R(ode45) for different scenarios of Problem 3.

**Figure 21:**
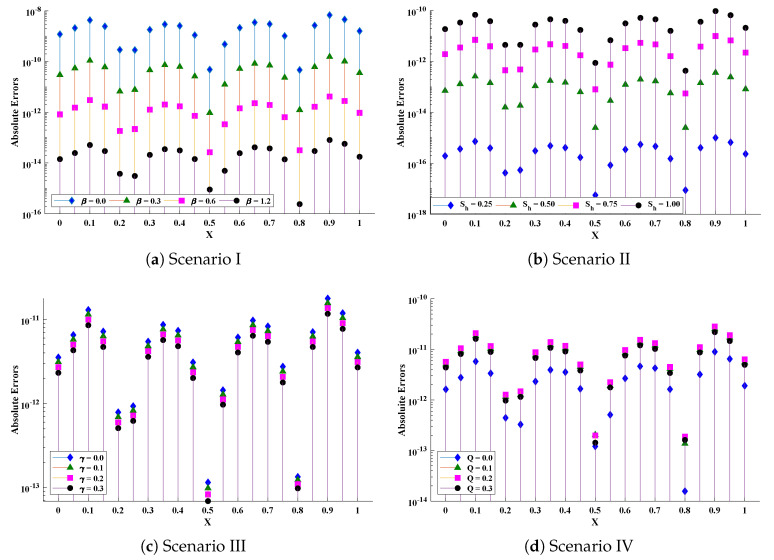
Absolute errors in approximate solutions of the proposed algorithm for different scenarios of problem 3.

Optimization of fitness equation Equation (80) for each scenario of problem 3 is conducted with the help of soft computing technique ENN-GNDO-IPA by executing it for 100 independent trials. The convergence of fitness value during the learning procedure is plotted in Figure 19. The approximate solutions for scenarios I, II, III, and IV by the proposed technique are dictated in Table 16. From Figure 20, it can be seen that nonlinear thermal conductivity parameters i.e., β, γ, and *Q* increases, the dimensionless temperature distribution in the fin decreases, while, with the increase in $S_{h}$, temperature distribution increases. Absolute errors in our solution are presented in Table 17 and graphically illustrated through Figure 21. It can be seen that absolute errors for each scenario lie around $1.59\times 10^{-9}$ to $3.76\times 10^{-15}$, $9.67\times 10^{-11}$ to $5.59\times 10^{-18}$, $1.15\times 10^{-11}$ to $6.90\times 10^{-14}$, and $1.19\times 10^{-11}$ to $1.57\times 10^{-14}$.

**Figure 22:**
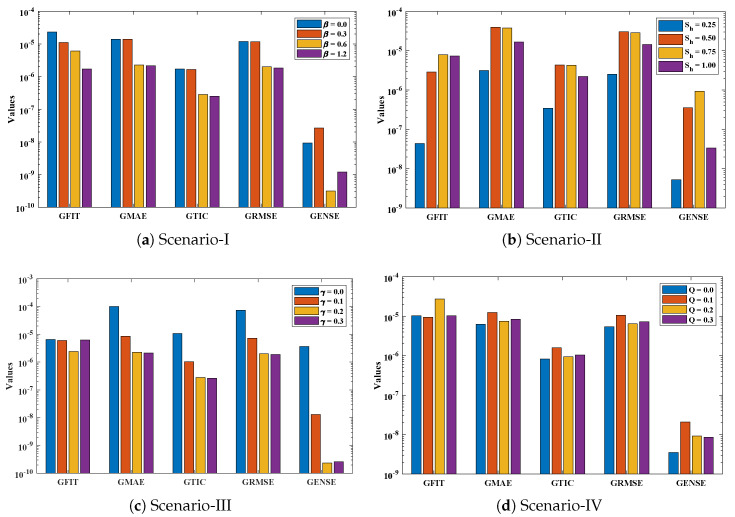
Comparative analysis on global parametric values of Fitness, MAE, TIC, RMSE, and ENSE.

**Figure 23:**
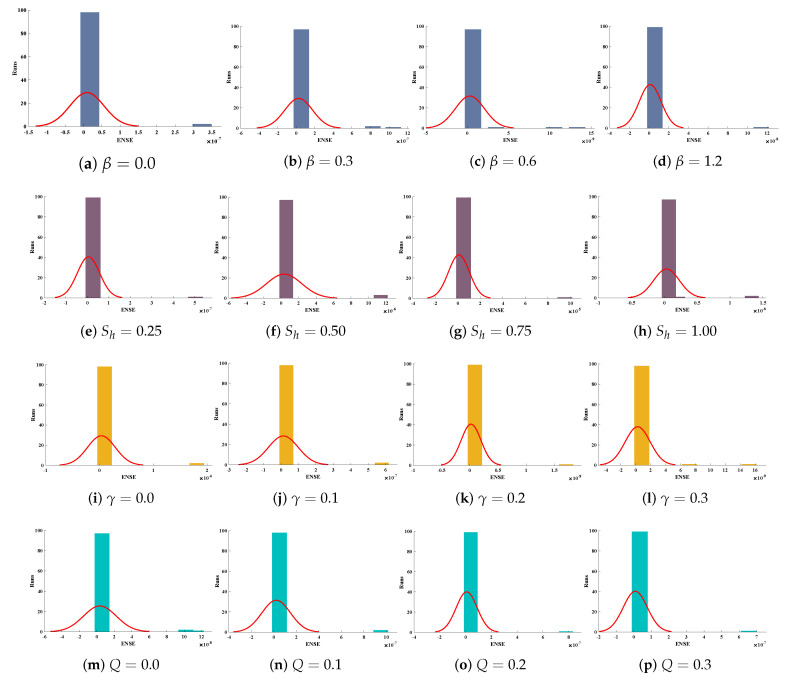
Normal probability curves for values of ENSE for scenario-I, II, III and IV of problem 3.

The accuracy of the proposed algorithm is measured by the results of fitness function and performance indicators. Table 18 and Table 19 show that the design scheme is convergent, and results are approaching zero. Bar graphs are plotted in Figure 22, which shows that mean values of fitness function, MAE, TIC, RMSE, and ENSE for different scenarios lie around $10^{-5}$ to $10^{-8}$, $10^{-5}$ to $10^{-6}$, $10^{-5}$ to $10^{-7}$, $10^{-5}$ to $10^{-6}$ and $10^{-8}$ to $10^{-10}$, respectively. Furthermore, normal probability curves given in Figure 23 show the robustness of technique. Unknown neurons used in the process of optimization for the best solution of each scenario are presented in Table 20, Table 21, Table 22 and Table 23.

Problem 4: Metallic annular fin with temperature dependent thermal conductivity.

In this problem, heat transfer and temperature distribution in metallic annular fin with temperature dependent thermal conductivity have been investigated by using the proposed algorithm. An unsupervised objective function for the problem is given as

(81) $$\begin{array}{c} \mathrm{Minimize}\;\zeta =\frac{1}{M}\sum_{m=1}^{M}{\left(\frac{d^{2}{\hat{y}}_{m}}{dx^{2}}+\beta {\left(\frac{d{\hat{y}}_{m}}{dx}\right)}^{2}+\beta {\hat{y}}_{m}\frac{d^{2}{\hat{y}}_{m}}{dx^{2}}+\frac{\beta (\lambda -1)}{\left(1+(\lambda -1)x\right)}{\hat{y}}_{m}\frac{d{\hat{y}}_{m}}{dx}+\frac{\left(\lambda -1\right)}{\left(1+(\lambda -1)x\right)}\frac{d{\hat{y}}_{m}}{dx}-\frac{2Bi{\left(\lambda -1\right)}^{2}}{\delta }{\hat{y}}_{m}\right)}^{2}\\ +\frac{1}{2}\left({\left(\hat{y}\left(0\right)-1\right)}^{2}+{\left(\hat{y^{\prime }}\left(1\right)-0\right)}^{2}\right), \end{array}$$

**Figure 24:**
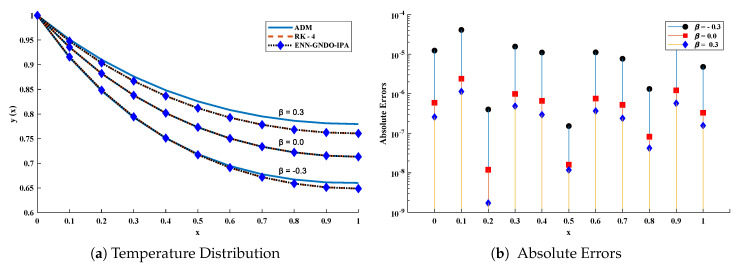
Temperature distribution and absolute errors in annular fin for different values of β with δ=1/3, λ=2 and Bi=0.1 in problem 4.

**Figure 25:**
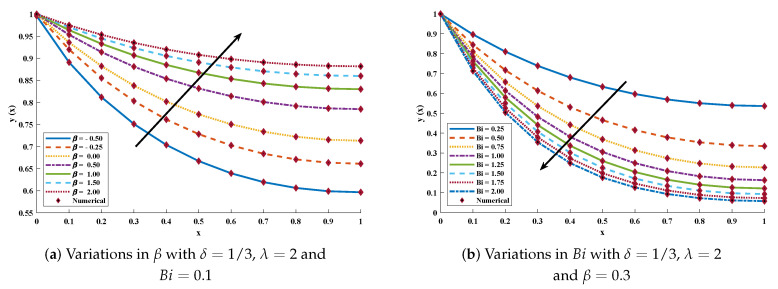
Temperature distribution for some values of β and Bi in problem 4.

Furthermore, to briefly study the model, different scenarios are considered depending on variations in several parameters such as β and Bi. Approximate solutions obtained by the ENN-GNDO-SQP algorithm for different values of β are dictated in Table 24 and graphically illustrated through Figure 24. Absolute errors for some values of β lie around $10^{-6}$ to $10^{-10}$. Influence of variations in β(−0.50,−0.25,0.00,0.50,1.00,1.50,2.00) and Bi(0.25,0.50,0.75,1.00,1.25,1.50,1.75,2.00) on temperature distribution are shown through Figure 25. It can be observed that temperature distribution of the annular fin increases with an increase in β, while it decreases with Bi.

**Figure 26:**
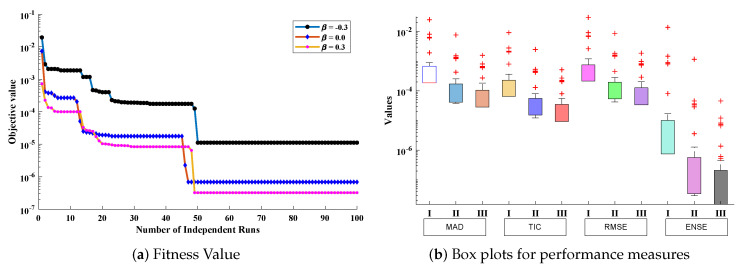
Convergence and boxplot analysis for fitness function and performance measures to validate the accuracy of design scheme.

**Figure 27:**
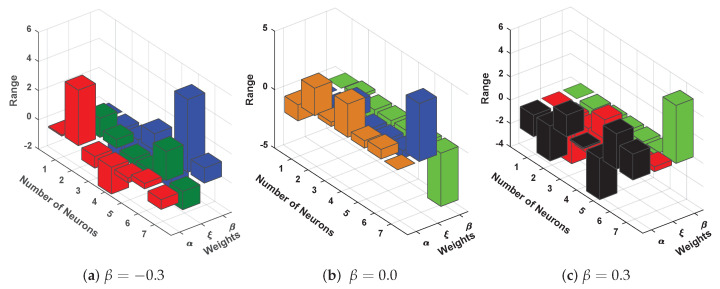
Unknown neurons in ENN structure obtained by the design algorithm for the best solution of different values of β in problem-4.

Furthermore, to validate the accuracy of the design scheme, 100 independent executions have been carried out. The results for the convergence of fitness function in each run are shown in Figure 26a. The results of fitness function for different values of β lie around $10^{-4}$ to $10^{-7}$. Boxplot analysis shows that mean values for MAD, TIC, RMSE, and ENSE lie around $10^{-3}$ to $10^{-5}$, $10^{-4}$ to $10^{-6}$, $10^{-3}$ to $10^{-5}$, and $10^{-6}$ to $10^{-9}$, respectively. Plots of optimized neurons for obtaining best solutions are shown in Figure 27. Results dictate that the design algorithm achieved accurate and overlapping results with minimum absolute errors in comparison with the techniques available in the latest literature.

## 6. Conclusions

In this paper, we have investigated different heat transfer problems arising in various engineering fields. The mathematical models of these problems are presented by highly nonlinear differential equations with initial and boundary conditions. We summarize our finding as follows:

- A novel computing framework is designed for solving nonlinear problems using neural networks model based on Euler polynomials. The model is further optimized by using the global optimization mechanism of the generalized normal distribution optimization (GNDO) algorithm and the brilliance of local search mechanism of the interior point algorithm. The proposed technique is called the ENN-GNDO-IPA algorithm.
- The accuracy of the proposed technique is demonstrated by the comparison of results with numerical solver RK-4 (ode45), and state-of-the-art algorithms including VIM, HPM, DTM, ADM, and exact solutions. Statistics illustrate that the proposed algorithm reaches accuracy with absolute errors having precision of up to 13–14 decimal places.
- The results dictate that the temperature distribution of problems 1, 2, 3, and 4 increases with increases in $\varepsilon_{2}$, ε, β, γ, *Q* and γ while possessing an inverse relation with $\varepsilon_{1}$, $S_{h}$ and Bi.
- The magnitude of fitness value calculated during 100 autonomous executions for each scenario of different problems shows the smoothness and convergence of the proposed technique, which further validates its worth.
- Extensive graphical and numerical analysis of performance indicators including MAE, TIC, RMSE, and ENSE and their global values further validate the correctness, stability, and reliability of the design scheme. In the future, the idea of Euler polynomials based artificial neural networks can be extended to solve partial and fractional differential equations representing various real world problems.

## Figures and Tables

**Table 1:** Parameter setting of GNDO and IPA.

Methods	Parameters	Setting	Parameters	Setting
GNDO	Population Creation	Random	Weights(Lower,Upper)	(−25, 25)
	Fitness bound	$10^{-20}$	Function tolerance	$10^{-25}$
	Decision variables	19	Search agents	60
	Selection	Stochastic Uniform	Constraint tolerance	$10^{-20}$
IPA	Start point	Best weights of GNDO	Hessian	BFGS
	X-tolerance	$10^{-20}$	Max. function evaluations	100,000
	Iterations	1000	Function tolerance	$10^{-25}$

**Table 2:** Approximate solutions for different cases of scenario-I of problem 1.

x	$\varepsilon_{2}=0$	$\varepsilon_{2}=0.1$	$\varepsilon_{2}=0.2$	$\varepsilon_{2}=0.3$	$\varepsilon_{2}=0.4$	$\varepsilon_{2}=0.5$	$\varepsilon_{2}=0.6$	$\varepsilon_{2}=0.7$	$\varepsilon_{2}=0.8$	$\varepsilon_{2}=0.9$	$\varepsilon_{2}=1.0$
0.0	1	0.999999	0.999999	0.999995	0.999846	0.999965	0.999925	0.999955	0.999992	0.999992	0.999999
0.1	0.904837	0.897138	0.889674	0.882425	0.875001	0.868496	0.861778	0.855200	0.848744	0.842393	0.836130
0.2	0.818730	0.806761	0.795445	0.784715	0.773870	0.764790	0.755496	0.746592	0.738041	0.729807	0.721862
0.3	0.740818	0.726716	0.713629	0.701435	0.689600	0.679331	0.669259	0.659750	0.650747	0.642201	0.634066
0.4	0.670320	0.655396	0.641720	0.629118	0.617445	0.606603	0.596475	0.586985	0.578061	0.569643	0.561678
0.5	0.606530	0.591591	0.578023	0.565619	0.554216	0.543682	0.533903	0.524793	0.516276	0.508287	0.500762
0.6	0.548811	0.534327	0.521243	0.509332	0.498437	0.488345	0.479008	0.470306	0.462159	0.454500	0.447273
0.7	0.496585	0.482847	0.470504	0.459322	0.448765	0.439748	0.431095	0.423064	0.415574	0.408562	0.401970
0.8	0.449328	0.436482	0.424992	0.414626	0.404715	0.396591	0.388668	0.381345	0.374545	0.368205	0.362272
0.9	0.406569	0.394660	0.384033	0.374463	0.365658	0.357845	0.350552	0.343815	0.337559	0.331726	0.326265
1.0	0.367879	0.356905	0.347120	0.338311	0.330514	0.323002	0.316271	0.310040	0.304241	0.298820	0.293729

**Table 3:** Approximate solutions for different cases of scenario-II of problem 1.

x	$\varepsilon_{1}=0$	$\varepsilon_{1}=0.1$	$\varepsilon_{1}=0.2$	$\varepsilon_{1}=0.3$	$\varepsilon_{1}=0.4$	$\varepsilon_{1}=0.5$	$\varepsilon_{1}=0.6$	$\varepsilon_{1}=0.7$	$\varepsilon_{1}=0.8$	$\varepsilon_{1}=0.9$	$\varepsilon_{1}=1.0$
0	0.999329	0.999644	0.999804	0.999888	0.999934	0.999960	0.999975	0.999984	0.999990	0.999993	0.999995
0.1	0.836130	0.848157	0.858531	0.867584	0.875562	0.882647	0.888982	0.894680	0.899831	0.904510	0.908779
0.2	0.721862	0.738346	0.753119	0.766427	0.778468	0.789405	0.799375	0.808493	0.816858	0.824556	0.831658
0.3	0.634066	0.652280	0.669058	0.684526	0.698797	0.711981	0.724177	0.735475	0.745958	0.755703	0.764777
0.4	0.561678	0.580739	0.598594	0.615299	0.630920	0.645522	0.659175	0.671946	0.683900	0.695099	0.705603
0.5	0.500762	0.519504	0.537925	0.555336	0.571774	0.587280	0.601899	0.615681	0.628673	0.640926	0.652486
0.6	0.447273	0.466637	0.485146	0.502807	0.519631	0.535635	0.550842	0.565280	0.578982	0.591983	0.604317
0.7	0.401970	0.420745	0.438907	0.456425	0.473275	0.489446	0.504935	0.519748	0.533897	0.547402	0.560285
0.8	0.362272	0.380230	0.397815	0.414958	0.431607	0.447723	0.463278	0.478259	0.492661	0.506486	0.519744
0.9	0.326265	0.343529	0.360563	0.377294	0.393662	0.409615	0.425114	0.440133	0.454654	0.468667	0.482170
1	0.293729	0.310334	0.326794	0.343051	0.359048	0.374733	0.390062	0.404999	0.419518	0.433600	0.447233

**Table 4:** Absolute errors in solutions obtained by proposed technique for variations in $\varepsilon_{2}$ with $\varepsilon_{1}=0$.

*x*	$\varepsilon_{2}=0$	$\varepsilon_{2}=0.1$	$\varepsilon_{2}=0.2$	$\varepsilon_{2}=0.3$	$\varepsilon_{2}=0.4$	$\varepsilon_{2}=0.5$	$\varepsilon_{2}=0.6$	$\varepsilon_{2}=0.7$	$\varepsilon_{2}=0.8$	$\varepsilon_{2}=0.9$	$\varepsilon_{2}=1.0$
0	2.23 × 10${}^{-14}$	1.98 × 10${}^{-9}$	2.12 × 10${}^{-8}$	1.01 × 10${}^{-7}$	1.33 × 10${}^{-5}$	8.68 × 10${}^{-7}$	1.94 × 10${}^{-6}$	3.89 × 10${}^{-6}$	7.14 × 10${}^{-6}$	1.22 × 10${}^{-5}$	1.98 × 10${}^{-5}$
0.1	2.16 × 10${}^{-13}$	1.99 × 10${}^{-8}$	2.08 × 10${}^{-7}$	9.71 × 10${}^{-7}$	4.94 × 10${}^{-5}$	7.89 × 10${}^{-6}$	1.72 × 10${}^{-5}$	3.35 × 10${}^{-5}$	5.99 × 10${}^{-5}$	1.00 × 10${}^{-4}$	1.58 × 10${}^{-4}$
0.2	7.06 × 10${}^{-14}$	8.55 × 10${}^{-9}$	9.36 × 10${}^{-8}$	4.51 × 10${}^{-7}$	2.33 × 10${}^{-8}$	3.84 × 10${}^{-6}$	8.53 × 10${}^{-6}$	1.69 × 10${}^{-5}$	3.07 × 10${}^{-5}$	5.20 × 10${}^{-5}$	8.32 × 10${}^{-5}$
0.3	1.20 × 10${}^{-13}$	8.24 × 10${}^{-9}$	7.89 × 10${}^{-8}$	3.44 × 10${}^{-7}$	1.86 × 10${}^{-5}$	2.51 × 10${}^{-6}$	5.24 × 10${}^{-6}$	9.79 × 10${}^{-6}$	1.69 × 10${}^{-5}$	2.72 × 10${}^{-5}$	4.16 × 10${}^{-5}$
0.4	9.80 × 10${}^{-15}$	1.77 × 10${}^{-9}$	2.03 × 10${}^{-8}$	1.01 × 10${}^{-7}$	1.01 × 10${}^{-5}$	8.84 × 10${}^{-7}$	1.98 × 10${}^{-6}$	3.96 × 10${}^{-6}$	7.23 × 10${}^{-6}$	1.23 × 10${}^{-5}$	1.98 × 10${}^{-5}$
0.5	1.38 × 10${}^{-13}$	9.71 × 10${}^{-9}$	9.39 × 10${}^{-8}$	4.13 × 10${}^{-7}$	5.32 × 10${}^{-7}$	8.83 × 10${}^{-7}$	4.55 × 10${}^{-7}$	2.58 × 10${}^{-7}$	1.34 × 10${}^{-6}$	3.45 × 10${}^{-6}$	3.29 × 10${}^{-6}$
0.6	1.52 × 10${}^{-14}$	3.64 × 10${}^{-10}$	2.53 × 10${}^{-9}$	8.40 × 10${}^{-9}$	1.23 × 10${}^{-5}$	3.78 × 10${}^{-8}$	6.24 × 10${}^{-8}$	9.28 × 10${}^{-8}$	1.27 × 10${}^{-7}$	1.61 × 10${}^{-7}$	1.93 × 10${}^{-7}$
0.7	1.04 × 10${}^{-13}$	7.53 × 10${}^{-9}$	7.34 × 10${}^{-8}$	3.25 × 10${}^{-7}$	7.52 × 10${}^{-6}$	2.44 × 10${}^{-6}$	5.16 × 10${}^{-6}$	9.78 × 10${}^{-6}$	1.71 × 10${}^{-5}$	2.79 × 10${}^{-5}$	4.33 × 10${}^{-5}$
0.8	7.39 × 10${}^{-14}$	3.41 × 10${}^{-9}$	3.01 × 10${}^{-8}$	1.24 × 10${}^{-7}$	1.43 × 10${}^{-6}$	8.42 × 10${}^{-7}$	1.71 × 10${}^{-6}$	3.12 × 10${}^{-6}$	5.28 × 10${}^{-6}$	8.38 × 10${}^{-6}$	1.27 × 10${}^{-5}$
0.9	1.84 × 10${}^{-13}$	1.11 × 10${}^{-8}$	1.04 × 10${}^{-7}$	4.53 × 10${}^{-7}$	1.76 × 10${}^{-5}$	3.30 × 10${}^{-6}$	6.90 × 10${}^{-6}$	1.30 × 10${}^{-5}$	2.24 × 10${}^{-5}$	3.64 × 10${}^{-5}$	5.62 × 10${}^{-5}$
1	1.71 × 10${}^{-14}$	1.10 × 10${}^{-9}$	1.05 × 10${}^{-8}$	4.60 × 10${}^{-8}$	4.81 × 10${}^{-6}$	3.41 × 10${}^{-7}$	7.16 × 10${}^{-7}$	1.35 × 10${}^{-6}$	2.36 × 10${}^{-6}$	3.84 × 10${}^{-6}$	5.95 × 10${}^{-6}$

**Table 5:** Absolute errors in solutions obtained by proposed technique for variations in $\varepsilon_{1}$ with $\varepsilon_{2}=1$.

*x*	$\varepsilon_{1}=0$	$\varepsilon_{1}=0.1$	$\varepsilon_{1}=0.2$	$\varepsilon_{1}=0.3$	$\varepsilon_{1}=0.4$	$\varepsilon_{1}=0.5$	$\varepsilon_{1}=0.6$	$\varepsilon_{1}=0.7$	$\varepsilon_{1}=0.8$	$\varepsilon_{1}=0.9$	$\varepsilon_{1}=1.0$
0.0	1.98 × 10${}^{-5}$	9.39 × 10${}^{-6}$	4.69 × 10${}^{-6}$	2.45 × 10${}^{-6}$	1.34 × 10${}^{-6}$	7.59 × 10${}^{-7}$	4.44 × 10${}^{-7}$	2.68 × 10${}^{-7}$	1.66 × 10${}^{-7}$	1.05 × 10${}^{-7}$	6.80 × 10${}^{-8}$
0.1	1.58 × 10${}^{-4}$	8.08 × 10${}^{-5}$	4.29 × 10${}^{-5}$	2.35 × 10${}^{-5}$	1.33 × 10${}^{-5}$	7.80 × 10${}^{-6}$	4.68 × 10${}^{-6}$	2.88 × 10${}^{-6}$	1.81 × 10${}^{-6}$	1.16 × 10${}^{-6}$	7.60 × 10${}^{-7}$
0.2	8.32 × 10${}^{-5}$	4.40 × 10${}^{-5}$	2.40 × 10${}^{-5}$	1.35 × 10${}^{-5}$	7.81 × 10${}^{-6}$	4.63 × 10${}^{-6}$	2.81 × 10${}^{-6}$	1.74 × 10${}^{-6}$	1.10 × 10${}^{-6}$	7.10 × 10${}^{-7}$	4.65 × 10${}^{-7}$
0.3	4.16 × 10${}^{-5}$	2.17 × 10${}^{-5}$	1.17 × 10${}^{-5}$	6.57 × 10${}^{-6}$	3.79 × 10${}^{-6}$	2.25 × 10${}^{-6}$	1.38 × 10${}^{-6}$	8.61 × 10${}^{-7}$	5.51 × 10${}^{-7}$	3.59 × 10${}^{-7}$	2.39 × 10${}^{-7}$
0.4	1.98 × 10${}^{-5}$	1.09 × 10${}^{-5}$	6.18 × 10${}^{-6}$	3.59 × 10${}^{-6}$	2.13 × 10${}^{-6}$	1.29 × 10${}^{-6}$	7.99 × 10${}^{-7}$	5.04 × 10${}^{-7}$	3.23 × 10${}^{-7}$	2.10 × 10${}^{-7}$	1.39 × 10${}^{-7}$
0.5	3.29 × 10${}^{-6}$	2.83 × 10${}^{-5}$	1.57 × 10${}^{-5}$	8.93 × 10${}^{-6}$	5.24 × 10${}^{-6}$	3.16 × 10${}^{-6}$	1.95 × 10${}^{-6}$	1.23 × 10${}^{-6}$	7.90 × 10${}^{-7}$	5.18 × 10${}^{-7}$	3.46 × 10${}^{-7}$
0.6	1.93 × 10${}^{-7}$	7.24 × 10${}^{-8}$	2.74 × 10${}^{-8}$	1.05 × 10${}^{-8}$	4.14 × 10${}^{-9}$	1.70 × 10${}^{-9}$	7.45 × 10${}^{-10}$	3.58 × 10${}^{-10}$	1.94 × 10${}^{-10}$	1.19 × 10${}^{-10}$	8.23 × 10${}^{-11}$
0.7	4.33 × 10${}^{-5}$	2.37 × 10${}^{-5}$	1.34 × 10${}^{-5}$	7.75 × 10${}^{-6}$	4.62 × 10${}^{-6}$	2.82 × 10${}^{-6}$	1.76 × 10${}^{-6}$	1.12 × 10${}^{-6}$	7.26 × 10${}^{-7}$	4.79 × 10${}^{-7}$	3.21 × 10${}^{-7}$
0.8	1.27 × 10${}^{-5}$	6.70 × 10${}^{-6}$	3.66 × 10${}^{-6}$	2.07 × 10${}^{-6}$	1.20 × 10${}^{-6}$	7.20 × 10${}^{-7}$	4.43 × 10${}^{-7}$	2.79 × 10${}^{-7}$	1.79 × 10${}^{-7}$	1.18 × 10${}^{-7}$	7.88 × 10${}^{-8}$
0.9	5.62 × 10${}^{-5}$	3.08 × 10${}^{-5}$	1.74 × 10${}^{-5}$	1.02 × 10${}^{-5}$	6.07 × 10${}^{-6}$	3.72 × 10${}^{-6}$	2.33 × 10${}^{-6}$	1.49 × 10${}^{-6}$	9.69 × 10${}^{-7}$	6.42 × 10${}^{-7}$	4.32 × 10${}^{-7}$
1.0	5.95 × 10${}^{-6}$	3.30 × 10${}^{-6}$	1.89 × 10${}^{-6}$	1.11 × 10${}^{-6}$	6.71 × 10${}^{-7}$	4.15 × 10${}^{-7}$	2.62 × 10${}^{-7}$	1.68 × 10${}^{-7}$	1.10 × 10${}^{-7}$	7.33 × 10${}^{-8}$	4.96 × 10${}^{-8}$

**Table 6:** Performance analysis on absolute errors in terms of minimum and mean values obtained by the proposed algorithm during 100 independent executions for each case of scenario-I of problem 1.

	$\varepsilon_{2}=0.0$		$\varepsilon_{2}=0.2$		$\varepsilon_{2}=0.4$		$\varepsilon_{2}=0.6$		$\varepsilon_{2}=0.8$		$\varepsilon_{2}=1.0$	
	Min	Mean	Min	Mean	Min	Mean	Min	Mean	Min	Mean	Min	Mean
0.00	2.23 × 10${}^{-14}$	7.01 × 10${}^{-7}$	2.12 × 10${}^{-8}$	3.38 × 10${}^{-6}$	3.32 × 10${}^{-7}$	7.52 × 10${}^{-5}$	1.94 × 10${}^{-6}$	8.62 × 10${}^{-5}$	7.14 × 10${}^{-6}$	3.63 × 10${}^{-4}$	1.98 × 10${}^{-5}$	9.94 × 10${}^{-4}$
0.05	3.46 × 10${}^{-13}$	8.35 × 10${}^{-8}$	4.54 × 10${}^{-7}$	2.49 × 10${}^{-6}$	7.45 × 10${}^{-6}$	2.48 × 10${}^{-5}$	4.44 × 10${}^{-5}$	1.10 × 10${}^{-4}$	1.65 × 10${}^{-4}$	3.41 × 10${}^{-4}$	4.60 × 10${}^{-4}$	9.98 × 10${}^{-4}$
0.10	2.16 × 10${}^{-13}$	2.43 × 10${}^{-8}$	2.08 × 10${}^{-7}$	5.38 × 10${}^{-6}$	3.10 × 10${}^{-6}$	4.11 × 10${}^{-5}$	1.72 × 10${}^{-5}$	1.54 × 10${}^{-4}$	5.99 × 10${}^{-5}$	4.94 × 10${}^{-4}$	1.58 × 10${}^{-4}$	1.46 × 10${}^{-3}$
0.15	5.05 × 10${}^{-15}$	1.64 × 10${}^{-7}$	8.44 × 10${}^{-11}$	3.99 × 10${}^{-6}$	6.51 × 10${}^{-10}$	3.82 × 10${}^{-5}$	2.86 × 10${}^{-8}$	8.33 × 10${}^{-5}$	1.07 × 10${}^{-7}$	3.02 × 10${}^{-4}$	1.07 × 10${}^{-6}$	8.17 × 10${}^{-4}$
0.20	7.06 × 10${}^{-14}$	3.10 × 10${}^{-7}$	3.63 × 10${}^{-11}$	1.82 × 10${}^{-6}$	2.32 × 10${}^{-8}$	3.09 × 10${}^{-5}$	1.76 × 10${}^{-9}$	3.39 × 10${}^{-5}$	6.25 × 10${}^{-10}$	1.47 × 10${}^{-4}$	1.60 × 10${}^{-8}$	3.27 × 10${}^{-4}$
0.25	1.67 × 10${}^{-13}$	3.78 × 10${}^{-7}$	1.52 × 10${}^{-7}$	8.51 × 10${}^{-7}$	2.20 × 10${}^{-6}$	2.61 × 10${}^{-5}$	1.19 × 10${}^{-5}$	2.64 × 10${}^{-5}$	4.04 × 10${}^{-5}$	9.50 × 10${}^{-5}$	1.05 × 10${}^{-4}$	2.14 × 10${}^{-4}$
0.30	1.20 × 10${}^{-13}$	3.55 × 10${}^{-7}$	2.01 × 10${}^{-8}$	9.66 × 10${}^{-7}$	8.84 × 10${}^{-10}$	2.21 × 10${}^{-5}$	1.22 × 10${}^{-6}$	3.43 × 10${}^{-5}$	1.03 × 10${}^{-5}$	9.36 × 10${}^{-5}$	4.32 × 10${}^{-6}$	2.71 × 10${}^{-4}$
0.35	2.11 × 10${}^{-14}$	2.67 × 10${}^{-7}$	4.96 × 10${}^{-9}$	1.39 × 10${}^{-6}$	4.26 × 10${}^{-8}$	1.73 × 10${}^{-5}$	1.44 × 10${}^{-7}$	3.85 × 10${}^{-5}$	3.06 × 10${}^{-7}$	1.03 × 10${}^{-4}$	4.85 × 10${}^{-7}$	3.23 × 10${}^{-4}$
0.40	9.80 × 10${}^{-15}$	1.54 × 10${}^{-7}$	2.03 × 10${}^{-8}$	1.63 × 10${}^{-6}$	3.36 × 10${}^{-7}$	1.25 × 10${}^{-5}$	1.98 × 10${}^{-6}$	3.56 × 10${}^{-5}$	1.37 × 10${}^{-6}$	1.08 × 10${}^{-4}$	1.98 × 10${}^{-5}$	3.19 × 10${}^{-4}$
0.45	8.60 × 10${}^{-14}$	5.95 × 10${}^{-8}$	3.97 × 10${}^{-9}$	1.59 × 10${}^{-6}$	1.13 × 10${}^{-6}$	9.22 × 10${}^{-6}$	5.66 × 10${}^{-6}$	3.00 × 10${}^{-5}$	2.23 × 10${}^{-6}$	1.08 × 10${}^{-4}$	2.36 × 10${}^{-5}$	2.82 × 10${}^{-4}$
0.50	2.37 × 10${}^{-16}$	1.34 × 10${}^{-8}$	2.67 × 10${}^{-9}$	1.42 × 10${}^{-6}$	7.15 × 10${}^{-10}$	8.63 × 10${}^{-6}$	3.02 × 10${}^{-8}$	2.62 × 10${}^{-5}$	7.25 × 10${}^{-7}$	1.02 × 10${}^{-4}$	8.05 × 10${}^{-8}$	2.43 × 10${}^{-4}$
0.55	9.43 × 10${}^{-14}$	2.88 × 10${}^{-8}$	4.74 × 10${}^{-8}$	1.24 × 10${}^{-6}$	5.85 × 10${}^{-7}$	1.04 × 10${}^{-5}$	2.83 × 10${}^{-6}$	2.52 × 10${}^{-5}$	8.85 × 10${}^{-6}$	9.14 × 10${}^{-5}$	2.14 × 10${}^{-5}$	2.17 × 10${}^{-4}$
0.60	1.52 × 10${}^{-14}$	9.89 × 10${}^{-8}$	2.53 × 10${}^{-9}$	1.06 × 10${}^{-6}$	1.98 × 10${}^{-8}$	1.30 × 10${}^{-5}$	6.24 × 10${}^{-8}$	2.49 × 10${}^{-5}$	1.27 × 10${}^{-7}$	7.64 × 10${}^{-5}$	1.93 × 10${}^{-7}$	1.94 × 10${}^{-4}$
0.65	1.37 × 10${}^{-14}$	1.99 × 10${}^{-7}$	1.85 × 10${}^{-8}$	8.56 × 10${}^{-7}$	2.81 × 10${}^{-7}$	1.47 × 10${}^{-5}$	1.57 × 10${}^{-6}$	2.25 × 10${}^{-5}$	8.31 × 10${}^{-7}$	5.96 × 10${}^{-5}$	4.77 × 10${}^{-6}$	1.64 × 10${}^{-4}$
0.70	1.04 × 10${}^{-13}$	2.90 × 10${}^{-7}$	7.34 × 10${}^{-8}$	6.75 × 10${}^{-7}$	2.07 × 10${}^{-7}$	1.46 × 10${}^{-5}$	5.16 × 10${}^{-6}$	1.77 × 10${}^{-5}$	1.37 × 10${}^{-6}$	4.64 × 10${}^{-5}$	4.32 × 10${}^{-5}$	1.32 × 10${}^{-4}$
0.75	1.57 × 10${}^{-13}$	3.33 × 10${}^{-7}$	2.34 × 10${}^{-9}$	7.27 × 10${}^{-7}$	6.02 × 10${}^{-7}$	1.35 × 10${}^{-5}$	3.36 × 10${}^{-6}$	1.50 × 10${}^{-5}$	1.23 × 10${}^{-6}$	4.78 × 10${}^{-5}$	1.46 × 10${}^{-5}$	1.32 × 10${}^{-4}$
0.80	1.71 × 10${}^{-15}$	2.96 × 10${}^{-7}$	5.19 × 10${}^{-9}$	1.21 × 10${}^{-6}$	1.20 × 10${}^{-7}$	1.32 × 10${}^{-5}$	1.71 × 10${}^{-6}$	2.14 × 10${}^{-5}$	2.97 × 10${}^{-6}$	7.53 × 10${}^{-5}$	1.27 × 10${}^{-5}$	2.01 × 10${}^{-4}$
0.85	2.12 × 10${}^{-15}$	1.80 × 10${}^{-7}$	4.43 × 10${}^{-9}$	1.94 × 10${}^{-6}$	7.05 × 10${}^{-8}$	1.42 × 10${}^{-5}$	4.04 × 10${}^{-7}$	3.71 × 10${}^{-5}$	4.00 × 10${}^{-8}$	1.21 × 10${}^{-4}$	3.69 × 10${}^{-6}$	3.16 × 10${}^{-4}$
0.90	1.84 × 10${}^{-13}$	4.23 × 10${}^{-8}$	1.04 × 10${}^{-7}$	2.05 × 10${}^{-6}$	1.36 × 10${}^{-6}$	1.32 × 10${}^{-5}$	6.90 × 10${}^{-6}$	4.46 × 10${}^{-5}$	7.01 × 10${}^{-6}$	1.33 × 10${}^{-4}$	5.55 × 10${}^{-5}$	3.40 × 10${}^{-4}$
0.95	3.14 × 10${}^{-13}$	3.68 × 10${}^{-8}$	1.47 × 10${}^{-7}$	7.45 × 10${}^{-7}$	1.85 × 10${}^{-6}$	7.28 × 10${}^{-6}$	9.10 × 10${}^{-6}$	1.98 × 10${}^{-5}$	1.29 × 10${}^{-5}$	5.46 × 10${}^{-5}$	2.87 × 10${}^{-5}$	1.33 × 10${}^{-4}$
0.10	1.71 × 10${}^{-14}$	4.69 × 10${}^{-7}$	1.05 × 10${}^{-8}$	1.26 × 10${}^{-6}$	1.40 × 10${}^{-7}$	1.99 × 10${}^{-5}$	7.16 × 10${}^{-7}$	2.31 × 10${}^{-5}$	1.46 × 10${}^{-7}$	7.38 × 10${}^{-5}$	5.77 × 10${}^{-6}$	2.05 × 10${}^{-4}$

**Table 7:** Performance analysis on absolute errors in terms of minimum and mean values obtained by the proposed algorithm during 100 independent executions for each case of scenario-II of problem 1.

	$\varepsilon_{1}=0.0$		$\varepsilon_{1}=0.2$		$\varepsilon_{1}=0.4$		$\varepsilon_{1}=0.6$		$\varepsilon_{1}=0.8$		$\varepsilon_{1}=1.0$	
	Min	Mean	Min	Mean	Min	Mean	Min	Mean	Min	Mean	Min	Mean
0.00	1.98 × 10${}^{-5}$	9.94 × 10${}^{-4}$	4.69 × 10${}^{-6}$	2.77 × 10${}^{-4}$	1.34 × 10${}^{-6}$	7.77 × 10${}^{-5}$	4.44 × 10${}^{-7}$	7.92 × 10${}^{-5}$	1.66 × 10${}^{-7}$	4.19 × 10${}^{-5}$	6.80 × 10${}^{-8}$	2.24 × 10${}^{-5}$
0.05	4.60 × 10${}^{-4}$	9.98 × 10${}^{-4}$	1.23 × 10${}^{-4}$	3.29 × 10${}^{-4}$	3.78 × 10${}^{-5}$	1.15 × 10${}^{-4}$	1.30 × 10${}^{-5}$	5.97 × 10${}^{-5}$	4.96 × 10${}^{-6}$	2.81 × 10${}^{-5}$	2.02 × 10${}^{-6}$	1.65 × 10${}^{-5}$
0.10	1.58 × 10${}^{-4}$	1.46 × 10${}^{-3}$	4.29 × 10${}^{-5}$	4.67 × 10${}^{-4}$	1.33 × 10${}^{-5}$	1.57 × 10${}^{-4}$	4.68 × 10${}^{-6}$	1.18 × 10${}^{-4}$	1.81 × 10${}^{-6}$	6.08 × 10${}^{-5}$	7.60 × 10${}^{-7}$	3.69 × 10${}^{-5}$
0.15	1.07 × 10${}^{-6}$	8.17 × 10${}^{-4}$	3.68 × 10${}^{-7}$	2.55 × 10${}^{-4}$	1.28 × 10${}^{-7}$	8.16 × 10${}^{-5}$	4.58 × 10${}^{-8}$	8.47 × 10${}^{-5}$	1.70 × 10${}^{-8}$	4.63 × 10${}^{-5}$	1.31 × 10${}^{-9}$	2.76 × 10${}^{-5}$
0.20	1.60 × 10${}^{-8}$	3.27 × 10${}^{-4}$	2.94 × 10${}^{-9}$	1.03 × 10${}^{-4}$	2.12 × 10${}^{-7}$	3.15 × 10${}^{-5}$	2.11 × 10${}^{-9}$	3.87 × 10${}^{-5}$	3.89 × 10${}^{-8}$	2.22 × 10${}^{-5}$	2.60 × 10${}^{-10}$	1.21 × 10${}^{-5}$
0.25	1.05 × 10${}^{-4}$	2.14 × 10${}^{-4}$	3.01 × 10${}^{-5}$	7.65 × 10${}^{-5}$	9.80 × 10${}^{-6}$	2.61 × 10${}^{-5}$	3.55 × 10${}^{-6}$	1.79 × 10${}^{-5}$	1.41 × 10${}^{-6}$	9.78 × 10${}^{-6}$	5.31 × 10${}^{-7}$	4.53 × 10${}^{-6}$
0.30	4.32 × 10${}^{-6}$	2.71 × 10${}^{-4}$	1.17 × 10${}^{-5}$	9.85 × 10${}^{-5}$	3.79 × 10${}^{-6}$	3.61 × 10${}^{-5}$	1.38 × 10${}^{-6}$	1.87 × 10${}^{-5}$	5.51 × 10${}^{-7}$	9.08 × 10${}^{-6}$	2.09 × 10${}^{-7}$	5.04 × 10${}^{-6}$
0.35	4.85 × 10${}^{-7}$	3.23 × 10${}^{-4}$	9.08 × 10${}^{-8}$	1.12 × 10${}^{-4}$	2.17 × 10${}^{-8}$	4.08 × 10${}^{-5}$	6.86 × 10${}^{-9}$	2.69 × 10${}^{-5}$	2.79 × 10${}^{-9}$	1.35 × 10${}^{-5}$	8.07 × 10${}^{-10}$	8.83 × 10${}^{-6}$
0.40	1.98 × 10${}^{-5}$	3.19 × 10${}^{-4}$	6.18 × 10${}^{-6}$	1.05 × 10${}^{-4}$	2.13 × 10${}^{-6}$	3.73 × 10${}^{-5}$	7.99 × 10${}^{-7}$	3.27 × 10${}^{-5}$	5.59 × 10${}^{-9}$	1.75 × 10${}^{-5}$	1.13 × 10${}^{-7}$	1.19 × 10${}^{-5}$
0.45	2.36 × 10${}^{-5}$	2.82 × 10${}^{-4}$	1.11 × 10${}^{-6}$	8.90 × 10${}^{-5}$	3.30 × 10${}^{-7}$	3.10 × 10${}^{-5}$	1.63 × 10${}^{-7}$	3.35 × 10${}^{-5}$	5.67 × 10${}^{-8}$	1.90 × 10${}^{-5}$	5.13 × 10${}^{-8}$	1.25 × 10${}^{-5}$
0.50	8.05 × 10${}^{-8}$	2.43 × 10${}^{-4}$	5.76 × 10${}^{-7}$	7.81 × 10${}^{-5}$	7.37 × 10${}^{-7}$	2.70 × 10${}^{-5}$	4.87 × 10${}^{-7}$	3.01 × 10${}^{-5}$	1.48 × 10${}^{-7}$	1.77 × 10${}^{-5}$	4.63 × 10${}^{-8}$	1.10 × 10${}^{-5}$
0.55	2.14 × 10${}^{-5}$	2.17 × 10${}^{-4}$	6.14 × 10${}^{-6}$	7.46 × 10${}^{-5}$	2.01 × 10${}^{-6}$	2.60 × 10${}^{-5}$	7.36 × 10${}^{-7}$	2.46 × 10${}^{-5}$	2.98 × 10${}^{-7}$	1.44 × 10${}^{-5}$	8.86 × 10${}^{-8}$	8.12 × 10${}^{-6}$
0.60	1.93 × 10${}^{-7}$	1.94 × 10${}^{-4}$	2.74 × 10${}^{-8}$	7.19 × 10${}^{-5}$	4.14 × 10${}^{-9}$	2.53 × 10${}^{-5}$	7.45 × 10${}^{-10}$	1.86 × 10${}^{-5}$	1.93 × 10${}^{-10}$	1.01 × 10${}^{-5}$	8.23 × 10${}^{-11}$	5.19 × 10${}^{-6}$
0.65	4.77 × 10${}^{-6}$	1.64 × 10${}^{-4}$	5.78 × 10${}^{-7}$	6.24 × 10${}^{-5}$	5.10 × 10${}^{-8}$	2.23 × 10${}^{-5}$	5.58 × 10${}^{-10}$	1.33 × 10${}^{-5}$	1.29 × 10${}^{-9}$	6.56 × 10${}^{-6}$	1.34 × 10${}^{-10}$	3.36 × 10${}^{-6}$
0.70	4.32 × 10${}^{-5}$	1.32 × 10${}^{-4}$	1.34 × 10${}^{-5}$	4.77 × 10${}^{-5}$	4.62 × 10${}^{-6}$	1.75 × 10${}^{-5}$	1.76 × 10${}^{-6}$	1.13 × 10${}^{-5}$	2.63 × 10${}^{-8}$	5.54 × 10${}^{-6}$	2.81 × 10${}^{-7}$	3.66 × 10${}^{-6}$
0.75	1.46 × 10${}^{-5}$	1.32 × 10${}^{-4}$	3.85 × 10${}^{-7}$	4.25 × 10${}^{-5}$	1.87 × 10${}^{-7}$	1.64 × 10${}^{-5}$	7.64 × 10${}^{-8}$	1.53 × 10${}^{-5}$	3.30 × 10${}^{-8}$	8.69 × 10${}^{-6}$	2.14 × 10${}^{-8}$	6.55 × 10${}^{-6}$
0.80	1.27 × 10${}^{-5}$	2.01 × 10${}^{-4}$	3.66 × 10${}^{-6}$	6.46 × 10${}^{-5}$	1.20 × 10${}^{-6}$	2.48 × 10${}^{-5}$	4.43 × 10${}^{-7}$	2.59 × 10${}^{-5}$	1.79 × 10${}^{-7}$	1.55 × 10${}^{-5}$	4.61 × 10${}^{-8}$	1.10 × 10${}^{-5}$
0.85	3.69 × 10${}^{-6}$	3.16 × 10${}^{-4}$	1.31 × 10${}^{-6}$	1.09 × 10${}^{-4}$	4.86 × 10${}^{-7}$	4.02 × 10${}^{-5}$	1.95 × 10${}^{-7}$	3.70 × 10${}^{-5}$	8.35 × 10${}^{-8}$	2.16 × 10${}^{-5}$	9.94 × 10${}^{-9}$	1.38 × 10${}^{-5}$
0.90	5.55 × 10${}^{-5}$	3.40 × 10${}^{-4}$	1.74 × 10${}^{-5}$	1.23 × 10${}^{-4}$	6.07 × 10${}^{-6}$	4.42 × 10${}^{-5}$	2.33 × 10${}^{-6}$	3.42 × 10${}^{-5}$	3.37 × 10${}^{-7}$	1.87 × 10${}^{-5}$	3.21 × 10${}^{-7}$	1.08 × 10${}^{-5}$
0.95	2.87 × 10${}^{-5}$	1.33 × 10${}^{-4}$	1.30 × 10${}^{-5}$	5.01 × 10${}^{-5}$	7.45 × 10${}^{-6}$	1.75 × 10${}^{-5}$	2.82 × 10${}^{-6}$	1.06 × 10${}^{-5}$	7.64 × 10${}^{-9}$	5.06 × 10${}^{-6}$	4.62 × 10${}^{-7}$	2.44 × 10${}^{-6}$
0.10	5.77 × 10${}^{-6}$	2.05 × 10${}^{-4}$	1.89 × 10${}^{-6}$	6.76 × 10${}^{-5}$	6.71 × 10${}^{-7}$	2.64 × 10${}^{-5}$	2.62 × 10${}^{-7}$	2.47 × 10${}^{-5}$	1.10 × 10${}^{-7}$	1.47 × 10${}^{-5}$	1.69 × 10${}^{-8}$	1.05 × 10${}^{-5}$

**Table 8:** Statistical analysis of performance indicators for scenario-I or problem 1.

		Fit			MAE			TIC			RMSE			ENSE	
	Min	Mean	Std	Min	Mean	Std	Min	Mean	Std	Min	Mean	Std	Min	Mean	Std
$\varepsilon_{2}=0.0$	8.82 × 10${}^{-14}$	2.50 × 10${}^{-7}$	1.19 × 10${}^{-6}$	8.14 × 10${}^{-12}$	2.66 × 10${}^{-5}$	1.01 × 10${}^{-4}$	1.39 × 10${}^{-12}$	4.80 × 10${}^{-6}$	1.81 × 10${}^{-5}$	6.83 × 10${}^{-12}$	8.95 × 10${}^{-5}$	1.55 × 10${}^{-7}$	0	2.37 × 10${}^{-5}$	7.45 × 10${}^{-7}$
$\varepsilon_{2}=0.1$	6.69 × 10${}^{-9}$	9.79 × 10${}^{-7}$	2.43 × 10${}^{-6}$	1.65 × 10${}^{-10}$	9.10 × 10${}^{-5}$	1.53 × 10${}^{-4}$	2.91 × 10${}^{-11}$	1.69 × 10${}^{-5}$	2.89 × 10${}^{-5}$	1.41 × 10${}^{-10}$	8.18 × 10${}^{-5}$	1.40 × 10${}^{-4}$	0	4.72 × 10${}^{-7}$	1.28 × 10${}^{-6}$
$\varepsilon_{2}=0.2$	6.70 × 10${}^{-8}$	1.90 × 10${}^{-6}$	4.54 × 10${}^{-6}$	1.56 × 10${}^{-10}$	1.29 × 10${}^{-4}$	2.09 × 10${}^{-4}$	2.95 × 10${}^{-11}$	2.40 × 10${}^{-5}$	3.90 × 10${}^{-5}$	1.40 × 10${}^{-10}$	1.14 × 10${}^{-4}$	1.86 × 10${}^{-4}$	0	9.24 × 10${}^{-7}$	2.65 × 10${}^{-6}$
$\varepsilon_{2}=0.3$	3.03 × 10${}^{-7}$	8.50 × 10${}^{-6}$	2.07 × 10${}^{-5}$	4.64 × 10${}^{-10}$	2.80 × 10${}^{-4}$	5.00 × 10${}^{-4}$	9.61 × 10${}^{-11}$	5.25 × 10${}^{-5}$	9.42 × 10${}^{-5}$	4.50 × 10${}^{-10}$	2.46 × 10${}^{-4}$	4.41 × 10${}^{-4}$	0	5.15 × 10${}^{-6}$	1.57 × 10${}^{-5}$
$\varepsilon_{2}=0.4$	9.48 × 10${}^{-7}$	2.46 × 10${}^{-5}$	1.23 × 10${}^{-4}$	2.28 × 10${}^{-10}$	6.05 × 10${}^{-4}$	1.35 × 10${}^{-3}$	4.48 × 10${}^{-11}$	1.13 × 10${}^{-4}$	2.52 × 10${}^{-4}$	2.07 × 10${}^{-10}$	5.21 × 10${}^{-4}$	1.15 × 10${}^{-3}$	0	3.50 × 10${}^{-5}$	2.60 × 10${}^{-4}$
$\varepsilon_{2}=0.5$	2.37 × 10${}^{-6}$	3.19 × 10${}^{-5}$	7.25 × 10${}^{-5}$	7.66 × 10${}^{-10}$	6.54 × 10${}^{-4}$	1.11 × 10${}^{-3}$	1.31 × 10${}^{-10}$	1.25 × 10${}^{-4}$	2.13 × 10${}^{-4}$	5.97 × 10${}^{-10}$	5.68 × 10${}^{-4}$	9.67 × 10${}^{-4}$	0	2.73 × 10${}^{-5}$	9.42 × 10${}^{-5}$
$\varepsilon_{2}=0.6$	5.08 × 10${}^{-6}$	4.63 × 10${}^{-5}$	9.41 × 10${}^{-5}$	1.11 × 10${}^{-9}$	7.76 × 10${}^{-4}$	1.35 × 10${}^{-3}$	1.91 × 10${}^{-10}$	1.51 × 10${}^{-4}$	2.64 × 10${}^{-4}$	8.58 × 10${}^{-10}$	6.74 × 10${}^{-4}$	1.18 × 10${}^{-3}$	0	4.10 × 10${}^{-5}$	1.29 × 10${}^{-4}$
$\varepsilon_{2}=0.7$	9.78 × 10${}^{-6}$	1.34 × 10${}^{-4}$	4.26 × 10${}^{-4}$	2.47 × 10${}^{-9}$	1.62 × 10${}^{-3}$	3.70 × 10${}^{-3}$	4.86 × 10${}^{-10}$	3.19 × 10${}^{-4}$	7.17 × 10${}^{-4}$	2.15 × 10${}^{-9}$	1.40 × 10${}^{-3}$	3.11 × 10${}^{-3}$	1.11 × 10${}^{-16}$	2.81 × 10${}^{-4}$	1.82 × 10${}^{-3}$
$\varepsilon_{2}=0.8$	1.73 × 10${}^{-5}$	1.64 × 10${}^{-4}$	3.34 × 10${}^{-4}$	3.89 × 10${}^{-9}$	1.82 × 10${}^{-3}$	3.41 × 10${}^{-3}$	9.33 × 10${}^{-10}$	3.65 × 10${}^{-4}$	6.80 × 10${}^{-4}$	4.08 × 10${}^{-9}$	1.59 × 10${}^{-3}$	2.95 × 10${}^{-3}$	2.22 × 10${}^{-16}$	2.63 × 10${}^{-4}$	8.45 × 10${}^{-4}$
$\varepsilon_{2}=0.9$	2.87 × 10${}^{-5}$	1.14 × 10${}^{-4}$	1.24 × 10${}^{-4}$	3.74 × 10${}^{-8}$	1.23 × 10${}^{-3}$	1.69 × 10${}^{-3}$	7.04 × 10${}^{-9}$	2.49 × 10${}^{-4}$	3.44 × 10${}^{-4}$	3.04 × 10${}^{-8}$	1.08 × 10${}^{-3}$	1.48 × 10${}^{-3}$	2.54 × 10${}^{-14}$	7.83 × 10${}^{-5}$	1.35 × 10${}^{-4}$
$\varepsilon_{2}=1.0$	4.51 × 10${}^{-5}$	4.61 × 10${}^{-4}$	6.32 × 10${}^{-4}$	6.91 × 10${}^{-9}$	4.25 × 10${}^{-3}$	5.56 × 10${}^{-3}$	1.47 × 10${}^{-9}$	8.83 × 10${}^{-4}$	1.15 × 10${}^{-3}$	6.27 × 10${}^{-9}$	3.74 × 10${}^{-3}$	4.85 × 10${}^{-3}$	8.88 × 10${}^{-16}$	8.96 × 10${}^{-4}$	1.87 × 10${}^{-3}$

**Table 9:** Statistical analysis of performance indicators for scenario-II or problem 1.

		Fit			MAE			TIC			RMSE			ENSE	
	Min	Mean	Std	Min	Mean	Std	Min	Mean	Std	Min	Mean	Std	Min	Mean	Std
$\varepsilon_{1}=0.0$	4.51 × 10${}^{-5}$	4.61 × 10${}^{-4}$	6.32 × 10${}^{-4}$	6.91 × 10${}^{-9}$	4.25 × 10${}^{-3}$	5.56 × 10${}^{-3}$	1.47 × 10${}^{-9}$	8.83 × 10${}^{-4}$	1.15 × 10${}^{-3}$	6.27 × 10${}^{-9}$	3.74 × 10${}^{-3}$	4.85 × 10${}^{-3}$	8.88 × 10${}^{-16}$	8.96 × 10${}^{-4}$	1.87 × 10${}^{-3}$
$\varepsilon_{1}=0.1$	2.37 × 10${}^{-5}$	1.83 × 10${}^{-4}$	6.61 × 10${}^{-4}$	2.58 × 10${}^{-8}$	1.79 × 10${}^{-3}$	4.85 × 10${}^{-3}$	4.83 × 10${}^{-9}$	3.55 × 10${}^{-4}$	9.50 × 10${}^{-4}$	2.12 × 10${}^{-8}$	1.54 × 10${}^{-3}$	4.05 × 10${}^{-3}$	1.18 × 10${}^{-14}$	4.66 × 10${}^{-4}$	3.72 × 10${}^{-3}$
$\varepsilon_{1}=0.2$	1.29 × 10${}^{-5}$	1.42 × 10${}^{-4}$	2.69 × 10${}^{-4}$	5.21 × 10${}^{-10}$	1.72 × 10${}^{-3}$	2.90 × 10${}^{-3}$	9.86 × 10${}^{-11}$	3.31 × 10${}^{-4}$	5.55 × 10${}^{-4}$	4.45 × 10${}^{-10}$	1.49 × 10${}^{-3}$	2.48 × 10${}^{-3}$	0	1.91 × 10${}^{-4}$	6.11 × 10${}^{-4}$
$\varepsilon_{1}=0.3$	7.25 × 10${}^{-6}$	1.31 × 10${}^{-4}$	4.47 × 10${}^{-4}$	8.47 × 10${}^{-9}$	1.55 × 10${}^{-3}$	4.04 × 10${}^{-3}$	1.73 × 10${}^{-9}$	2.87 × 10${}^{-4}$	7.38 × 10${}^{-4}$	7.99 × 10${}^{-9}$	1.32 × 10${}^{-3}$	3.35 × 10${}^{-3}$	1.22 × 10${}^{-15}$	3.03 × 10${}^{-4}$	2.00 × 10${}^{-3}$
$\varepsilon_{1}=0.4$	4.20 × 10${}^{-6}$	4.68 × 10${}^{-5}$	9.92 × 10${}^{-5}$	6.67 × 10${}^{-10}$	7.37 × 10${}^{-4}$	1.35 × 10${}^{-3}$	1.14 × 10${}^{-10}$	1.34 × 10${}^{-4}$	2.46 × 10${}^{-4}$	5.38 × 10${}^{-10}$	6.34 × 10${}^{-4}$	1.16 × 10${}^{-3}$	0	3.69 × 10${}^{-5}$	1.25 × 10${}^{-4}$
$\varepsilon_{1}=0.5$	2.51 × 10${}^{-6}$	9.63 × 10${}^{-5}$	3.88 × 10${}^{-4}$	1.02 × 10${}^{-9}$	1.21 × 10${}^{-3}$	3.87 × 10${}^{-3}$	1.88 × 10${}^{-10}$	2.10 × 10${}^{-4}$	6.62 × 10${}^{-4}$	9.08 × 10${}^{-10}$	1.01 × 10${}^{-3}$	3.15 × 10${}^{-3}$	0	2.48 × 10${}^{-4}$	1.46 × 10${}^{-3}$
$\varepsilon_{1}=0.6$	1.53 × 10${}^{-6}$	4.00 × 10${}^{-5}$	9.23 × 10${}^{-5}$	1.49 × 10${}^{-9}$	6.84 × 10${}^{-4}$	1.32 × 10${}^{-3}$	2.97 × 10${}^{-10}$	1.19 × 10${}^{-4}$	2.26 × 10${}^{-4}$	1.46 × 10${}^{-9}$	5.84 × 10${}^{-4}$	1.11 × 10${}^{-3}$	0	3.24 × 10${}^{-5}$	1.11 × 10${}^{-4}$
$\varepsilon_{1}=0.7$	9.58 × 10${}^{-7}$	2.56 × 10${}^{-5}$	6.27 × 10${}^{-5}$	1.74 × 10${}^{-10}$	4.75 × 10${}^{-4}$	9.85 × 10${}^{-4}$	2.94 × 10${}^{-11}$	8.10 × 10${}^{-5}$	1.67 × 10${}^{-4}$	1.47 × 10${}^{-10}$	4.05 × 10${}^{-4}$	8.33 × 10${}^{-4}$	0	1.70 × 10${}^{-5}$	5.87 × 10${}^{-5}$
$\varepsilon_{1}=0.8$	6.12 × 10${}^{-7}$	2.15 × 10${}^{-5}$	5.43 × 10${}^{-5}$	2.58 × 10${}^{-10}$	4.33 × 10${}^{-4}$	8.71 × 10${}^{-4}$	4.08 × 10${}^{-11}$	7.24 × 10${}^{-5}$	1.43 × 10${}^{-4}$	2.08 × 10${}^{-10}$	3.68 × 10${}^{-4}$	7.28 × 10${}^{-4}$	0	1.31 × 10${}^{-5}$	4.90 × 10${}^{-5}$
$\varepsilon_{1}=0.9$	3.98 × 10${}^{-7}$	2.40 × 10${}^{-5}$	1.26 × 10${}^{-4}$	3.21 × 10${}^{-9}$	4.22 × 10${}^{-4}$	1.56 × 10${}^{-3}$	5.35 × 10${}^{-10}$	6.82 × 10${}^{-5}$	2.45 × 10${}^{-4}$	2.77 × 10${}^{-9}$	3.51 × 10${}^{-4}$	1.26 × 10${}^{-3}$	1.11 × 10${}^{-16}$	3.54 × 10${}^{-5}$	2.93 × 10${}^{-4}$
$\varepsilon_{1}=1.0$	2.64 × 10${}^{-7}$	1.28 × 10${}^{-5}$	2.59 × 10${}^{-5}$	1.45 × 10${}^{-9}$	3.16 × 10${}^{-4}$	5.01 × 10${}^{-4}$	2.07 × 10${}^{-10}$	5.16 × 10${}^{-5}$	8.15 × 10${}^{-5}$	1.09 × 10${}^{-9}$	2.71 × 10${}^{-4}$	4.28 × 10${}^{-4}$	0	4.64 × 10${}^{-6}$	1.15 × 10${}^{-5}$

**Table 10:** Unknown parameters in the ENN structure obtained for the optimization of fitness function corresponding to different scenarios of problem 1.

**Cases**		$\varepsilon_{2}=0.1$			$\varepsilon_{2}=0.3$			$\varepsilon_{2}=0.5$			$\varepsilon_{2}=0.7$			$\varepsilon_{2}=0.9$	
	$\alpha_{i}$	$\xi_{i}$	$\beta_{i}$	$\alpha_{i}$	$\xi_{i}$	$\beta_{i}$	$\alpha_{i}$	$\xi_{i}$	$\beta_{i}$	$\alpha_{i}$	$\xi_{i}$	$\beta_{i}$	$\alpha_{i}$	$\xi_{i}$	$\beta_{i}$
	5.03812			−29.26589			−19.99825			0.64562			22.56125		
	0.20313	−11.76389	5.84474	2.06891	23.92809	−18.36832	−8.09909	−5.48027	18.35398	−0.28764	−5.78693	8.75575	−22.00461	−4.99967	15.73748
	−0.13975	−2.51889	−3.91198	−0.51166	−8.96369	19.72460	7.81304	2.64892	14.87901	6.31582	2.15027	−2.57848	4.09324	9.02104	3.10481
Scenario I	0.05405	−2.05590	3.11470	29.83113	1.30071	−1.41738	1.09489	−1.59918	2.78812	−2.59084	−0.36333	4.29886	−0.03001	13.89997	21.83689
	1.15102	−1.06393	−0.15656	10.54092	−0.85454	3.17928	−9.41886	−0.41683	−3.21001	−19.98630	0.79878	2.21293	2.51064	−3.00268	−3.55055
	−19.75252	0.32612	1.41369	14.89994	−0.56987	0.12981	0.11127	0.85978	4.75097	0.06743	1.56355	5.38662	0.24364	−2.82351	−6.92593
	0.07603	0.91816	19.99014	0.51614	−0.87266	3.51774	−2.84× 10${}^{-9}$	19.99999	−16.76034	1.21522	−0.96519	−17.11512	3.99038	0.85387	0.69695
**Cases**		$\varepsilon_{1}=0.1$			$\varepsilon_{1}=0.3$			$\varepsilon_{1}=0.5$			$\varepsilon_{1}=0.7$			$\varepsilon_{1}=0.9$	
	20.61351			4.93273			2.26096			−2.03594			−0.19179		
	−1.10056	24.83025	24.05446	13.50719	−7.63775	−22.68861	0.48364	1.67689	−9.30659	0.31724	1.47179	23.15367	0.04003	24.87931	3.48124
	7.58588	6.01936	3.88696	5.38287	1.99249	11.24699	−5.35302	−3.54694	1.91702	−1.69009	3.31291	−1.91379	0.31027	−4.16164	−0.54261
Scenario II	5.56439	−3.50289	−2.63373	−20.21616	−0.10543	−3.69838	4.21289	2.22282	0.19709	−5.44338	0.58099	−0.46122	9.95183	−1.10317	−1.31553
	−1.36402	1.98193	−0.09751	−0.65021	0.25469	7.75734	−2.62507	−1.72823	0.98849	15.77756	0.48452	0.90529	−1.02714	−2.19302	−1.36089
	1.23025	−1.81427	−0.62571	24.81554	−0.48103	1.23781	−0.35268	−1.64339	0.81777	15.61507	−0.30881	1.77609	1.83599	−1.16699	0.57169
	2.58449	0.89955	18.06540	0.97909	−0.95956	−17.13953	2.20489	−0.76685	−1.72732	1.01387	−0.80439	5.98129	2.49767	0.64188	2.61856

**Table 11:** Approximate solutions for different cases of problem 2.

*x*	ε=0.0	ε=0.1	ε=0.2	ε=0.3	ε=0.4	ε=0.5	ε=0.6	ε=0.7	ε=0.8	ε=0.9	ε=1.0
0.0	1	1	1	1	1	1	1	1	1	1	1
0.1	0.904837	0.912765	0.919519	0.925334	0.930386	0.934814	0.938723	0.942198	0.9453069	0.948102	0.950630
0.2	0.818730	0.832555	0.844579	0.855104	0.864374	0.872586	0.879904	0.886458	0.8923587	0.897694	0.902541
0.3	0.740818	0.758896	0.774927	0.7891843	0.801906	0.813303	0.823550	0.832800	0.8411828	0.848805	0.855762
0.4	0.670320	0.691333	0.710304	0.727435	0.742919	0.756940	0.769665	0.781242	0.7918031	0.801462	0.810322
0.5	0.606530	0.629428	0.650450	0.669708	0.687334	0.703467	0.718243	0.731794	0.7442398	0.755691	0.766248
0.6	0.548811	0.572766	0.595103	0.615848	0.635065	0.652841	0.669272	0.684461	0.6985090	0.711512	0.723564
0.7	0.496585	0.520953	0.544002	0.565690	0.586016	0.605012	0.622731	0.639243	0.6546218	0.668946	0.682294
0.8	0.449328	0.473614	0.496893	0.519068	0.540082	0.559920	0.578593	0.596132	0.6125848	0.628006	0.642456
0.9	0.406569	0.430400	0.453524	0.475807	0.497151	0.517498	0.536820	0.555114	0.5723989	0.588703	0.604068
1.0	0.367879	0.390980	0.413651	0.435735	0.457105	0.477670	0.497369	0.516170	0.5340596	0.551044	0.567143

**Table 12:** Absolute errors in solutions obtained by the proposed technique for variations in specific heat for cooling of a lumped system.

*x*	ε=0.0	ε=0.1	ε=0.2	ε=0.3	ε=0.4	ε=0.5	ε=0.6	ε=0.7	ε=0.8	ε=0.9	ε=1.0
0.0	2.22 × 10${}^{-14}$	9.54 × 10${}^{-15}$	5.74 × 10${}^{-15}$	1.21 × 10${}^{-14}$	3.74 × 10${}^{-15}$	3.07 × 10${}^{-17}$	8.12 × 10${}^{-16}$	1.65 × 10${}^{-15}$	1.52 × 10${}^{-15}$	9.59 × 10${}^{-16}$	4.60 × 10${}^{-16}$
0.1	2.16 × 10${}^{-13}$	9.41 × 10${}^{-14}$	6.15 × 10${}^{-14}$	1.28 × 10${}^{-13}$	3.88 × 10${}^{-14}$	2.23 × 10${}^{-16}$	9.24 × 10${}^{-15}$	1.84 × 10${}^{-14}$	1.67 × 10${}^{-14}$	1.05 × 10${}^{-14}$	4.98 × 10${}^{-15}$
0.2	7.06 × 10${}^{-14}$	3.01 × 10${}^{-14}$	2.69 × 10${}^{-14}$	4.95 × 10${}^{-14}$	1.35 × 10${}^{-14}$	2.30 × 10${}^{-19}$	4.67 × 10${}^{-15}$	8.28 × 10${}^{-15}$	7.20 × 10${}^{-15}$	4.36 × 10${}^{-15}$	2.00 × 10${}^{-15}$
0.3	1.20 × 10${}^{-13}$	5.64 × 10${}^{-14}$	2.96 × 10${}^{-14}$	7.09 × 10${}^{-14}$	2.40 × 10${}^{-14}$	4.25 × 10${}^{-16}$	4.20 × 10${}^{-15}$	9.48 × 10${}^{-15}$	9.09 × 10${}^{-15}$	5.91 × 10${}^{-15}$	2.91 × 10${}^{-15}$
0.4	9.78 × 10${}^{-15}$	3.94 × 10${}^{-15}$	5.98 × 10${}^{-15}$	9.25 × 10${}^{-15}$	2.10 × 10${}^{-15}$	3.74 × 10${}^{-17}$	1.28 × 10${}^{-15}$	1.97 × 10${}^{-15}$	1.62 × 10${}^{-15}$	9.34 × 10${}^{-16}$	4.07 × 10${}^{-16}$
0.5	1.38 × 10${}^{-13}$	6.66 × 10${}^{-14}$	3.69 × 10${}^{-14}$	8.92 × 10${}^{-14}$	3.05 × 10${}^{-14}$	5.00 × 10${}^{-16}$	5.65 × 10${}^{-15}$	1.27 × 10${}^{-14}$	1.22 × 10${}^{-14}$	7.93 × 10${}^{-15}$	3.91 × 10${}^{-15}$
0.6	1.52 × 10${}^{-14}$	8.41 × 10${}^{-15}$	1.50 × 10${}^{-15}$	6.98 × 10${}^{-15}$	3.47 × 10${}^{-15}$	3.76 × 10${}^{-16}$	8.93 × 10${}^{-17}$	5.45 × 10${}^{-16}$	6.83 × 10${}^{-16}$	5.25 × 10${}^{-16}$	3.01 × 10${}^{-16}$
0.7	1.04 × 10${}^{-13}$	5.14 × 10${}^{-14}$	2.98 × 10${}^{-14}$	7.27 × 10${}^{-14}$	2.50 × 10${}^{-14}$	3.83 × 10${}^{-16}$	4.93 × 10${}^{-15}$	1.10 × 10${}^{-14}$	1.06 × 10${}^{-14}$	6.93 × 10${}^{-15}$	3.43 × 10${}^{-15}$
0.8	7.39 × 10${}^{-14}$	3.95 × 10${}^{-14}$	1.35 × 10${}^{-14}$	4.41 × 10${}^{-14}$	1.84 × 10${}^{-14}$	9.76 × 10${}^{-16}$	1.67 × 10${}^{-15}$	5.23 × 10${}^{-15}$	5.64 × 10${}^{-15}$	3.96 × 10${}^{-15}$	2.10 × 10${}^{-15}$
0.9	1.84 × 10${}^{-13}$	9.56 × 10${}^{-14}$	4.48 × 10${}^{-14}$	1.25 × 10${}^{-13}$	4.74 × 10${}^{-14}$	1.42 × 10${}^{-15}$	6.94 × 10${}^{-15}$	1.78 × 10${}^{-14}$	1.81 × 10${}^{-14}$	1.22 × 10${}^{-14}$	6.26 × 10${}^{-15}$
1.0	1.71 × 10${}^{-14}$	8.92 × 10${}^{-15}$	4.50 × 10${}^{-15}$	1.22 × 10${}^{-14}$	4.57 × 10${}^{-15}$	1.17 × 10${}^{-16}$	7.45 × 10${}^{-16}$	1.85 × 10${}^{-15}$	1.86 × 10${}^{-15}$	1.25 × 10${}^{-15}$	6.37 × 10${}^{-16}$

**Table 13:** Performance analysis on absolute errors in terms of minimum and mean values obtained by the proposed algorithm during 100 independent executions for different cases of problem 2.

	$\varepsilon_{1}=0.0$		$\varepsilon_{1}=0.2$		$\varepsilon_{1}=0.4$		$\varepsilon_{1}=0.6$		$\varepsilon_{1}=0.8$		$\varepsilon_{1}=1.0$	
	Min	Mean	Min	Mean	Min	Mean	Min	Mean	Min	Mean	Min	Mean
0.00	6.47 × 110${}^{-15}$	5.90 × 110${}^{-9}$	5.74 × 110${}^{-15}$	1.44 × 110${}^{-10}$	2.27 × 110${}^{-15}$	7.09 × 110${}^{-11}$	6.78 × 110${}^{-17}$	2.14 × 110${}^{-12}$	1.60 × 110${}^{-16}$	2.55 × 110${}^{-11}$	5.15 × 110${}^{-17}$	2.07 × 110${}^{-11}$
0.05	4.96 × 110${}^{-14}$	8.77 × 110${}^{-10}$	2.92 × 110${}^{-14}$	6.22 × 110${}^{-11}$	5.49 × 110${}^{-14}$	1.80 × 110${}^{-11}$	4.83 × 110${}^{-15}$	3.30 × 110${}^{-12}$	4.92 × 110${}^{-17}$	5.22 × 110${}^{-12}$	3.13 × 110${}^{-18}$	3.94 × 110${}^{-12}$
0.10	4.55 × 110${}^{-16}$	5.52 × 110${}^{-9}$	6.15 × 110${}^{-14}$	1.97 × 110${}^{-10}$	3.24 × 110${}^{-14}$	8.33 × 110${}^{-11}$	6.04 × 110${}^{-16}$	6.15 × 110${}^{-12}$	1.21 × 110${}^{-15}$	3.09 × 110${}^{-11}$	1.35 × 110${}^{-18}$	2.46 × 110${}^{-11}$
0.15	5.05 × 110${}^{-15}$	7.32 × 110${}^{-9}$	2.66 × 110${}^{-16}$	2.09 × 110${}^{-10}$	3.24 × 110${}^{-16}$	1.00 × 110${}^{-10}$	4.16 × 110${}^{-18}$	3.65 × 110${}^{-12}$	8.90 × 110${}^{-17}$	3.72 × 110${}^{-11}$	4.05 × 110${}^{-17}$	3.08 × 110${}^{-11}$
0.20	1.34 × 110${}^{-14}$	5.51 × 110${}^{-9}$	2.69 × 110${}^{-14}$	1.28 × 110${}^{-10}$	3.28 × 110${}^{-16}$	7.00 × 110${}^{-11}$	1.60 × 110${}^{-15}$	1.18 × 110${}^{-12}$	7.29 × 110${}^{-16}$	2.40 × 110${}^{-11}$	1.11 × 110${}^{-20}$	2.09 × 110${}^{-11}$
0.25	6.70 × 110${}^{-14}$	2.63 × 110${}^{-9}$	3.46 × 110${}^{-15}$	5.65 × 110${}^{-11}$	3.09 × 110${}^{-14}$	3.16 × 110${}^{-11}$	2.49 × 110${}^{-16}$	1.28 × 110${}^{-12}$	1.75 × 110${}^{-18}$	7.66 × 110${}^{-12}$	5.14 × 110${}^{-19}$	7.45 × 110${}^{-12}$
0.30	7.65 × 110${}^{-16}$	7.92 × 110${}^{-10}$	2.96 × 110${}^{-14}$	4.76 × 110${}^{-11}$	2.09 × 110${}^{-14}$	1.12 × 110${}^{-11}$	9.12 × 110${}^{-16}$	2.69 × 110${}^{-12}$	3.68 × 110${}^{-16}$	5.08 × 110${}^{-13}$	1.54 × 110${}^{-17}$	6.84 × 110${}^{-13}$
0.35	2.11 × 110${}^{-14}$	7.35 × 110${}^{-10}$	2.76 × 110${}^{-15}$	9.45 × 110${}^{-11}$	2.90 × 110${}^{-15}$	1.56 × 110${}^{-11}$	2.36 × 110${}^{-16}$	3.34 × 110${}^{-12}$	8.84 × 110${}^{-16}$	4.68 × 110${}^{-12}$	1.97 × 110${}^{-18}$	3.38 × 110${}^{-12}$
0.40	9.78 × 110${}^{-15}$	2.02 × 110${}^{-9}$	5.98 × 110${}^{-15}$	1.56 × 110${}^{-10}$	2.10 × 110${}^{-15}$	3.66 × 110${}^{-11}$	1.28 × 110${}^{-15}$	2.59 × 110${}^{-12}$	1.44 × 110${}^{-17}$	1.49 × 110${}^{-11}$	4.07 × 110${}^{-16}$	1.20 × 110${}^{-11}$
0.45	8.60 × 110${}^{-14}$	3.64 × 110${}^{-9}$	2.84 × 110${}^{-14}$	1.88 × 110${}^{-10}$	9.73 × 110${}^{-18}$	5.98 × 110${}^{-11}$	4.84 × 110${}^{-15}$	1.38 × 110${}^{-12}$	6.72 × 110${}^{-18}$	2.34 × 110${}^{-11}$	1.05 × 110${}^{-17}$	2.01 × 110${}^{-11}$
0.50	1.69 × 110${}^{-14}$	4.60 × 110${}^{-9}$	1.77 × 110${}^{-14}$	1.73 × 110${}^{-10}$	1.95 × 110${}^{-14}$	7.17 × 110${}^{-11}$	1.46 × 110${}^{-18}$	1.05 × 110${}^{-12}$	4.92 × 110${}^{-16}$	2.48 × 110${}^{-11}$	2.42 × 110${}^{-15}$	2.26 × 110${}^{-11}$
0.55	1.53 × 110${}^{-14}$	4.36 × 110${}^{-9}$	2.03 × 110${}^{-14}$	1.18 × 110${}^{-10}$	1.89 × 110${}^{-14}$	6.54 × 110${}^{-11}$	4.47 × 110${}^{-16}$	2.04 × 110${}^{-12}$	1.04 × 110${}^{-16}$	1.85 × 110${}^{-11}$	6.02 × 110${}^{-17}$	1.79 × 110${}^{-11}$
0.60	1.52 × 110${}^{-14}$	3.02 × 110${}^{-9}$	1.50 × 110${}^{-15}$	5.93 × 110${}^{-11}$	2.16 × 110${}^{-15}$	4.35 × 110${}^{-11}$	8.93 × 110${}^{-17}$	3.42 × 110${}^{-12}$	5.36 × 110${}^{-16}$	8.51 × 110${}^{-12}$	2.87 × 110${}^{-19}$	9.02 × 110${}^{-12}$
0.65	1.37 × 110${}^{-14}$	1.35 × 110${}^{-9}$	6.68 × 110${}^{-15}$	3.67 × 110${}^{-11}$	3.27 × 110${}^{-15}$	1.86 × 110${}^{-11}$	1.40 × 110${}^{-15}$	3.75 × 110${}^{-12}$	6.08 × 110${}^{-18}$	1.31 × 110${}^{-12}$	5.79 × 110${}^{-16}$	1.66 × 110${}^{-12}$
0.70	1.04 × 110${}^{-13}$	5.02 × 110${}^{-10}$	2.98 × 110${}^{-14}$	7.84 × 110${}^{-11}$	1.56 × 110${}^{-15}$	8.80 × 110${}^{-12}$	4.93 × 110${}^{-15}$	2.45 × 110${}^{-12}$	5.96 × 110${}^{-20}$	2.30 × 110${}^{-12}$	1.70 × 110${}^{-16}$	1.47 × 110${}^{-12}$
0.75	5.99 × 110${}^{-15}$	1.41 × 110${}^{-9}$	3.68 × 110${}^{-14}$	1.79 × 110${}^{-10}$	7.64 × 110${}^{-15}$	2.81 × 110${}^{-11}$	5.43 × 110${}^{-15}$	8.43 × 110${}^{-13}$	1.22 × 110${}^{-17}$	1.22 × 110${}^{-11}$	3.13 × 110${}^{-18}$	1.03 × 110${}^{-11}$
0.80	3.48 × 110${}^{-14}$	3.99 × 110${}^{-9}$	3.83 × 110${}^{-15}$	2.89 × 110${}^{-10}$	3.49 × 110${}^{-15}$	7.28 × 110${}^{-11}$	3.54 × 110${}^{-18}$	1.37 × 110${}^{-12}$	3.13 × 110${}^{-17}$	2.54 × 110${}^{-11}$	5.81 × 110${}^{-18}$	2.35 × 110${}^{-11}$
0.85	2.11 × 110${}^{-15}$	6.49 × 110${}^{-9}$	1.67 × 110${}^{-15}$	3.21 × 110${}^{-10}$	5.44 × 110${}^{-16}$	1.12 × 110${}^{-10}$	4.28 × 110${}^{-16}$	4.87 × 110${}^{-12}$	5.17 × 110${}^{-16}$	3.12 × 110${}^{-11}$	1.31 × 110${}^{-16}$	3.06 × 110${}^{-11}$
0.90	1.84 × 110${}^{-13}$	5.90 × 110${}^{-9}$	4.48 × 110${}^{-14}$	2.05 × 110${}^{-10}$	5.37 × 110${}^{-16}$	9.68 × 110${}^{-11}$	6.94 × 110${}^{-15}$	7.63 × 110${}^{-12}$	9.87 × 110${}^{-18}$	2.08 × 110${}^{-11}$	1.06 × 110${}^{-15}$	2.15 × 110${}^{-11}$
0.95	2.97 × 110${}^{-13}$	1.39 × 110${}^{-9}$	6.44 × 110${}^{-14}$	2.73 × 110${}^{-11}$	3.00 × 110${}^{-15}$	2.02 × 110${}^{-11}$	8.93 × 110${}^{-15}$	3.60 × 110${}^{-12}$	1.23 × 110${}^{-16}$	1.97 × 110${}^{-12}$	4.64 × 110${}^{-16}$	2.27 × 110${}^{-12}$
1.00	1.71 × 110${}^{-14}$	4.21 × 110${}^{-9}$	4.50 × 110${}^{-15}$	3.37 × 110${}^{-10}$	1.76 × 110${}^{-16}$	7.82 × 110${}^{-11}$	7.44 × 110${}^{-16}$	2.97 × 110${}^{-12}$	3.28 × 110${}^{-17}$	2.42 × 110${}^{-11}$	9.92 × 110${}^{-17}$	2.33 × 110${}^{-11}$

**Table 14:** Statistical analysis in terms of minimum, mean, and standard deviation of performance indicators for problem 2.

		Fit			MAE			TIC			RMSE			ENSE	
	Min	Mean	Std	Min	Mean	Std	Min	Mean	Std	Min	Mean	Std	Min	Mean	Std
ε=0.0	8.82 × 110${}^{-14}$	3.82 × 110${}^{-9}$	1.42 × 110${}^{-8}$	1.40 × 110${}^{-11}$	4.21 × 110${}^{-6}$	9.94 × 110${}^{-6}$	2.41 × 110${}^{-12}$	7.55 × 110${}^{-7}$	1.79 × 110${}^{-6}$	1.19 × 110${}^{-11}$	3.73 × 110${}^{-6}$	8.85 × 110${}^{-6}$	0	1.67 × 110${}^{-9}$	6.46 × 110${}^{-9}$
ε=0.1	4.22 × 110${}^{-14}$	5.44 × 110${}^{-8}$	1.99 × 110${}^{-7}$	5.76 × 110${}^{-13}$	1.30 × 110${}^{-5}$	4.39 × 110${}^{-5}$	9.53 × 110${}^{-14}$	2.26 × 110${}^{-6}$	7.64 × 110${}^{-6}$	4.84 × 110${}^{-13}$	1.15 × 110${}^{-5}$	3.88 × 110${}^{-5}$	0	2.88 × 110${}^{-8}$	1.05 × 110${}^{-7}$
ε=0.2	2.37 × 110${}^{-14}$	1.65 × 110${}^{-10}$	8.26 × 110${}^{-10}$	2.60 × 110${}^{-12}$	7.18 × 110${}^{-7}$	1.88 × 110${}^{-6}$	4.20 × 110${}^{-13}$	1.21 × 110${}^{-7}$	3.12 × 110${}^{-7}$	2.19 × 110${}^{-12}$	6.31 × 110${}^{-7}$	1.62 × 110${}^{-6}$	0	5.34 × 110${}^{-11}$	2.75 × 110${}^{-10}$
ε=0.3	5.64 × 110${}^{-14}$	1.14 × 110${}^{-12}$	3.20 × 110${}^{-12}$	6.65 × 110${}^{-13}$	9.00 × 110${}^{-8}$	9.96 × 110${}^{-8}$	1.06 × 110${}^{-13}$	1.50 × 110${}^{-8}$	1.68 × 110${}^{-8}$	5.67 × 110${}^{-13}$	8.01 × 110${}^{-8}$	8.95 × 110${}^{-8}$	0	2.30 × 110${}^{-13}$	7.14 × 110${}^{-13}$
ε=0.4	1.92 × 110${}^{-14}$	5.85 × 110${}^{-11}$	2.06 × 110${}^{-10}$	3.39 × 110${}^{-13}$	3.78 × 110${}^{-7}$	9.85 × 110${}^{-7}$	5.42 × 110${}^{-14}$	6.13 × 110${}^{-8}$	1.58 × 110${}^{-7}$	2.95 × 110${}^{-13}$	3.34 × 110${}^{-7}$	8.63 × 110${}^{-7}$	0	1.37 × 110${}^{-11}$	4.93 × 110${}^{-11}$
ε=0.5	4.08 × 110${}^{-16}$	8.09 × 110${}^{-12}$	2.66 × 110${}^{-11}$	2.04 × 110${}^{-13}$	1.87 × 110${}^{-7}$	2.97 × 110${}^{-7}$	3.42 × 110${}^{-14}$	2.96 × 110${}^{-8}$	4.65 × 110${}^{-8}$	1.90 × 110${}^{-13}$	1.65 × 110${}^{-7}$	2.58 × 110${}^{-7}$	0	1.47 × 110${}^{-12}$	5.13 × 110${}^{-12}$
ε=0.6	3.66 × 110${}^{-15}$	3.40 × 110${}^{-12}$	8.92 × 110${}^{-12}$	1.63 × 110${}^{-13}$	1.31 × 110${}^{-7}$	1.51 × 110${}^{-7}$	2.39 × 110${}^{-14}$	2.05 × 110${}^{-8}$	2.39 × 110${}^{-8}$	1.35 × 110${}^{-13}$	1.16 × 110${}^{-7}$	1.35 × 110${}^{-7}$	0	4.66 × 110${}^{-13}$	1.32 × 110${}^{-12}$
ε=0.7	8.09 × 110${}^{-15}$	5.04 × 110${}^{-12}$	3.56 × 110${}^{-11}$	9.31 × 110${}^{-12}$	9.19 × 110${}^{-8}$	2.56 × 110${}^{-7}$	1.26 × 110${}^{-12}$	1.43 × 110${}^{-8}$	3.96 × 110${}^{-8}$	7.20 × 110${}^{-12}$	8.23 × 110${}^{-8}$	2.27 × 110${}^{-7}$	0	8.36 × 110${}^{-13}$	6.44 × 110${}^{-12}$
ε=0.8	7.75 × 110${}^{-15}$	1.83 × 110${}^{-11}$	6.68 × 110${}^{-11}$	1.69 × 110${}^{-13}$	1.81 × 110${}^{-7}$	4.77 × 110${}^{-7}$	2.79 × 110${}^{-14}$	2.75 × 110${}^{-8}$	7.23 × 110${}^{-8}$	1.63 × 110${}^{-13}$	1.60 × 110${}^{-7}$	4.21 × 110${}^{-7}$	0	2.88 × 110${}^{-12}$	1.05 × 110${}^{-11}$
ε=0.9	5.04 × 110${}^{-15}$	9.75 × 110${}^{-12}$	4.50 × 110${}^{-11}$	3.06 × 110${}^{-13}$	1.05 × 110${}^{-7}$	3.31 × 110${}^{-7}$	4.77 × 110${}^{-14}$	1.57 × 110${}^{-8}$	4.95 × 110${}^{-8}$	2.82 × 110${}^{-13}$	9.27 × 110${}^{-8}$	2.92 × 110${}^{-7}$	0	1.31 × 110${}^{-12}$	6.01 × 110${}^{-12}$
ε=1.0	2.49 × 110${}^{-15}$	1.64 × 110${}^{-11}$	5.98 × 110${}^{-11}$	7.35 × 110${}^{-13}$	1.66 × 110${}^{-7}$	4.15 × 110${}^{-7}$	9.41 × 110${}^{-14}$	2.42 × 110${}^{-8}$	6.03 × 110${}^{-8}$	5.62 × 110${}^{-13}$	1.44 × 110${}^{-7}$	3.60 × 110${}^{-7}$	0	2.12 × 110${}^{-12}$	7.35 × 110${}^{-12}$

**Table 15:** Unknown parameters in ENN structure obtained for the optimization of fitness function corresponding to different values of specific heat in problem 2.

Cases		ε=0.1			ε=0.3			ε=0.5			ε=0.7			ε=0.9	
	$\alpha_{i}$	$\xi_{i}$	$\beta_{i}$	$\alpha_{i}$	$\xi_{i}$	$\beta_{i}$	$\alpha_{i}$	$\xi_{i}$	$\beta_{i}$	$\alpha_{i}$	$\xi_{i}$	$\beta_{i}$	$\alpha_{i}$	$\xi_{i}$	$\beta_{i}$
1	−3.29313			−1.33367			0.08412			0.92053			0.41421		
2	1.63245	−1.90667	3.37402	−3.08059	0.38598	−0.51162	−0.04137	1.96789	−4.08357	−1.32074	−0.06144	2.25837	0.75222	0.46659	−2.18731
3	−4.99414	0.11981	1.54312	−3.34379	−0.02204	0.84629	0.21703	0.33095	−1.88138	−4.81096	−0.10272	3.54709	0.51945	0.07941	−1.94952
4	0.90267	0.40497	2.22288	1.25799	0.19256	1.11115	−0.24400	0.20762	0.46318	−0.60085	0.10777	−3.81037	−1.52521	0.18720	−0.17280
5	−1.26927	−0.24044	−0.08419	3.41251	0.06195	1.05561	−1.79043	0.22548	0.47965	1.87630	0.00307	−0.03366	2.38286	0.19904	1.03043
6	−1.34392	−0.37639	0.12319	1.42534	0.19443	−0.06168	1.09474	0.29704	0.41810	−1.75045	−0.22331	0.66318	0.10556	−0.28989	−0.64407
7	−0.41808	−0.32367	4.95432	0.98631	0.19321	−4.87757	1.07912	−0.23851	−2.27322	1.05785	−0.21146	−2.73285	0.02419	0.28327	−3.36610

**Table 16:** Approximate solutions obtained by the proposed algorithm for different scenarios of problem 3.

		Scenario I				Scenario II				Scenario III				Scenario IV		
X	β=0.0	β=0.3	β=0.6	β=1.2	$S_{h}=0.25$	$S_{h}=0.50$	$S_{h}=0.75$	$S_{h}=1.00$	γ=0.0	γ=0.1	γ=0.2	γ=0.3	Q=0.0	Q=0.1	Q=0.2	Q=0.3
0.0	0.852030	0.872695	0.888497	0.910890	0.921172	0.858262	0.806319	0.762359	0.867161	0.875369	0.883666	0.892054	0.772484	0.800653	0.828567	0.856235
0.1	0.853321	0.873852	0.889536	0.911743	0.921947	0.859634	0.808163	0.764588	0.868389	0.876522	0.884743	0.893053	0.774638	0.802529	0.830171	0.857572
0.2	0.857213	0.877338	0.892662	0.914306	0.924274	0.863753	0.813707	0.771290	0.872087	0.879992	0.887982	0.896059	0.781112	0.808170	0.834995	0.861597
0.3	0.863772	0.883191	0.897902	0.918590	0.928158	0.870637	0.822983	0.782516	0.878288	0.885811	0.893416	0.901102	0.791950	0.817618	0.843080	0.868344
0.4	0.873104	0.891481	0.905301	0.924628	0.933607	0.880312	0.836044	0.798353	0.887053	0.894036	0.901094	0.908228	0.807222	0.830941	0.854489	0.877872
0.5	0.885366	0.902304	0.914924	0.932442	0.940631	0.892818	0.852969	0.818925	0.898465	0.904745	0.911091	0.917506	0.827030	0.848238	0.869315	0.890265
0.6	0.900764	0.915786	0.926852	0.942077	0.949244	0.908205	0.873858	0.844392	0.912632	0.918038	0.923501	0.929022	0.851506	0.869638	0.887680	0.905633
0.7	0.919559	0.932085	0.941189	0.953584	0.959465	0.926534	0.898838	0.874959	0.929691	0.934044	0.938442	0.942886	0.880818	0.895301	0.909732	0.924112
0.8	0.942081	0.951394	0.958059	0.967025	0.971315	0.947882	0.928062	0.910875	0.949805	0.952914	0.956056	0.959230	0.915169	0.925422	0.935654	0.945865
0.9	0.968731	0.973942	0.977606	0.982470	0.984817	0.972336	0.961711	0.952437	0.973167	0.974830	0.976510	0.978207	0.954800	0.960231	0.965659	0.971085
1.0	0.999999	1	1	1	1	1	1	1	1	1	1	1	1	1	1	1

**Table 17:** Absolute errors in our solutions for different scenarios of convective fin.

		Scenario I				Scenario II				Scenario III				Scenario IV		
X	β=0.0	β=0.3	β=0.6	β=1.2	$S_{h}=0.25$	$S_{h}=0.50$	$S_{h}=0.75$	$S_{h}=1.00$	γ=0.0	γ=0.1	γ=0.2	γ=0.3	Q=0.0	Q=0.1	Q=0.2	Q=0.3
0.0	1.19 × 10${}^{-9}$	2.98 × 10${}^{-11}$	8.24 × 10${}^{-13}$	1.42 × 10${}^{-14}$	1.95 × 10${}^{-16}$	7.12 × 10${}^{-14}$	1.96 × 10${}^{-12}$	1.89 × 10${}^{-11}$	3.54 × 10${}^{-12}$	3.12 × 10${}^{-12}$	2.71 × 10${}^{-12}$	2.32 × 10${}^{-12}$	1.62 × 10${}^{-12}$	5.03 × 10${}^{-12}$	5.69 × 10${}^{-12}$	4.38 × 10${}^{-12}$
0.1	4.29 × 10${}^{-9}$	1.09 × 10${}^{-10}$	3.03 × 10${}^{-12}$	5.11 × 10${}^{-14}$	7.25 × 10${}^{-16}$	2.62 × 10${}^{-13}$	7.18 × 10${}^{-12}$	6.89 × 10${}^{-11}$	1.30 × 10${}^{-11}$	1.15 × 10${}^{-11}$	9.97 × 10${}^{-12}$	8.54 × 10${}^{-12}$	5.77 × 10${}^{-12}$	1.84 × 10${}^{-11}$	2.09 × 10${}^{-11}$	1.61 × 10${}^{-11}$
0.2	2.91 × 10${}^{-10}$	6.69 × 10${}^{-12}$	1.83 × 10${}^{-13}$	3.76 × 10${}^{-15}$	4.21 × 10${}^{-17}$	1.60 × 10${}^{-14}$	4.56 × 10${}^{-13}$	4.53 × 10${}^{-12}$	7.87 × 10${}^{-13}$	6.90 × 10${}^{-13}$	5.97 × 10${}^{-13}$	5.09 × 10${}^{-13}$	4.43 × 10${}^{-13}$	1.16 × 10${}^{-12}$	1.27 × 10${}^{-12}$	9.71 × 10${}^{-13}$
0.3	1.80 × 10${}^{-9}$	4.60 × 10${}^{-11}$	1.27 × 10${}^{-12}$	2.09 × 10${}^{-14}$	3.09 × 10${}^{-16}$	1.10 × 10${}^{-13}$	2.99 × 10${}^{-12}$	2.84 × 10${}^{-11}$	5.46 × 10${}^{-12}$	4.82 × 10${}^{-12}$	4.20 × 10${}^{-12}$	3.60 × 10${}^{-12}$	2.32 × 10${}^{-12}$	7.63 × 10${}^{-12}$	8.73 × 10${}^{-12}$	6.75 × 10${}^{-12}$
0.4	2.55 × 10${}^{-9}$	6.21 × 10${}^{-11}$	1.71 × 10${}^{-12}$	3.13 × 10${}^{-14}$	4.10 × 10${}^{-16}$	1.50 × 10${}^{-13}$	4.13 × 10${}^{-12}$	4.00 × 10${}^{-11}$	7.35 × 10${}^{-12}$	6.47 × 10${}^{-12}$	5.62 × 10${}^{-12}$	4.81 × 10${}^{-12}$	3.55 × 10${}^{-12}$	1.05 × 10${}^{-11}$	1.18 × 10${}^{-11}$	9.08 × 10${}^{-12}$
0.5	4.84 × 10${}^{-11}$	9.50 × 10${}^{-13}$	2.64 × 10${}^{-14}$	9.00 × 10${}^{-16}$	5.59 × 10${}^{-18}$	2.50 × 10${}^{-15}$	8.07 × 10${}^{-14}$	8.87 × 10${}^{-13}$	1.15 × 10${}^{-13}$	9.83 × 10${}^{-14}$	8.29 × 10${}^{-14}$	6.90 × 10${}^{-14}$	1.21 × 10${}^{-13}$	2.05 × 10${}^{-13}$	1.99 × 10${}^{-13}$	1.43 × 10${}^{-13}$
0.6	2.14 × 10${}^{-9}$	5.23 × 10${}^{-11}$	1.43 × 10${}^{-12}$	2.44 × 10${}^{-14}$	3.49 × 10${}^{-16}$	1.24 × 10${}^{-13}$	3.35 × 10${}^{-12}$	3.17 × 10${}^{-11}$	6.12 × 10${}^{-12}$	5.41 × 10${}^{-12}$	4.72 × 10${}^{-12}$	4.05 × 10${}^{-12}$	2.67 × 10${}^{-12}$	8.46 × 10${}^{-12}$	9.69 × 10${}^{-12}$	7.53 × 10${}^{-12}$
0.7	3.01 × 10${}^{-9}$	7.07 × 10${}^{-11}$	1.94 × 10${}^{-12}$	3.76 × 10${}^{-14}$	4.65 × 10${}^{-16}$	1.71 × 10${}^{-13}$	4.73 × 10${}^{-12}$	4.58 × 10${}^{-11}$	8.29 × 10${}^{-12}$	7.30 × 10${}^{-12}$	6.34 × 10${}^{-12}$	5.42 × 10${}^{-12}$	4.26 × 10${}^{-12}$	1.19 × 10${}^{-11}$	1.33 × 10${}^{-11}$	1.02 × 10${}^{-11}$
0.8	4.67 × 10${}^{-11}$	1.24 × 10${}^{-12}$	3.17 × 10${}^{-14}$	2.39 × 10${}^{-16}$	8.76 × 10${}^{-18}$	2.52 × 10${}^{-15}$	5.59 × 10${}^{-14}$	4.37 × 10${}^{-13}$	1.34 × 10${}^{-13}$	1.23 × 10${}^{-13}$	1.10 × 10${}^{-13}$	9.78 × 10${}^{-14}$	1.57 × 10${}^{-14}$	1.37 × 10${}^{-13}$	1.89 × 10${}^{-13}$	1.62 × 10${}^{-13}$
0.9	6.77 × 10${}^{-9}$	1.54 × 10${}^{-10}$	4.17 × 10${}^{-12}$	8.07 × 10${}^{-14}$	1.01 × 10${}^{-15}$	3.67 × 10${}^{-13}$	1.01 × 10${}^{-11}$	9.67 × 10${}^{-11}$	1.78 × 10${}^{-11}$	1.57 × 10${}^{-11}$	1.37 × 10${}^{-11}$	1.17 × 10${}^{-11}$	8.96 × 10${}^{-12}$	2.52 × 10${}^{-11}$	2.83 × 10${}^{-11}$	2.19 × 10${}^{-11}$
1.0	1.59 × 10${}^{-9}$	3.55 × 10${}^{-11}$	9.47 × 10${}^{-13}$	1.76 × 10${}^{-14}$	2.33 × 10${}^{-16}$	8.30 × 10${}^{-14}$	2.24 × 10${}^{-12}$	2.12 × 10${}^{-11}$	4.04 × 10${}^{-12}$	3.58 × 10${}^{-12}$	3.12 × 10${}^{-12}$	2.69 × 10${}^{-12}$	1.91 × 10${}^{-12}$	5.55 × 10${}^{-12}$	6.33 × 10${}^{-12}$	4.95 × 10${}^{-12}$

**Table 18:** Analysis on fitness evaluation and performance measures by the ENN-GNDO-IPA algorithm during 100 independent executions for different scenarios of problem 3.

**Cases**			β=0.0			β=0.3			β=0.6			β=1.2	
		**Min**	**Mean**	**Std**	**Min**	**Mean**	**Std**	**Min**	**Mean**	**Std**	**Min**	**Mean**	**Std**
	Fit	2.16 × 10${}^{-9}$	2.31 × 10${}^{-5}$	1.31 × 10${}^{-4}$	5.17 × 10${}^{-11}$	1.11 × 10${}^{-5}$	6.19 × 10${}^{-5}$	1.42 × 10${}^{-12}$	6.07 × 10${}^{-6}$	3.46 × 10${}^{-5}$	2.57 × 10${}^{-14}$	1.72 × 10${}^{-6}$	1.18 × 10${}^{-5}$
	MAE	1.50 × 10${}^{-10}$	1.40 × 10${}^{-5}$	2.96 × 10${}^{-5}$	3.63 × 10${}^{-11}$	1.39 × 10${}^{-5}$	5.47 × 10${}^{-5}$	1.66 × 10${}^{-12}$	2.27 × 10${}^{-6}$	5.77 × 10${}^{-6}$	2.26 × 10${}^{-12}$	2.14 × 10${}^{-6}$	1.21 × 10${}^{-5}$
Scenario I	TIC	1.68 × 10${}^{-11}$	1.70 × 10${}^{-6}$	3.36 × 10${}^{-6}$	3.77 × 10${}^{-12}$	1.65 × 10${}^{-6}$	6.36 × 10${}^{-6}$	1.90 × 10${}^{-13}$	2.82 × 10${}^{-7}$	6.95 × 10${}^{-7}$	2.59 × 10${}^{-13}$	2.51 × 10${}^{-7}$	1.40 × 10${}^{-6}$
	RMSE	1.17 × 10${}^{-10}$	1.18 × 10${}^{-5}$	2.34 × 10${}^{-5}$	2.66 × 10${}^{-11}$	1.17 × 10${}^{-5}$	4.48 × 10${}^{-5}$	1.36 × 10${}^{-12}$	2.01 × 10${}^{-6}$	4.96 × 10${}^{-6}$	1.88 × 10${}^{-12}$	1.82 × 10${}^{-6}$	1.02 × 10${}^{-5}$
	ENSE	0	9.32 × 10${}^{-9}$	4.66 × 10${}^{-8}$	0	2.67 × 10${}^{-8}$	1.50 × 10${}^{-7}$	0	3.14 × 10${}^{-10}$	1.77 × 10${}^{-9}$	0	1.20 × 10${}^{-9}$	1.12 × 10${}^{-8}$
**Cases**			$S_{h}=0.25$			$S_{h}=0.50$			$S_{h}=0.75$			$S_{h}=1.00$	
		**Min**	**Mean**	**Std**	**Min**	**Mean**	**Std**	**Min**	**Mean**	**Std**	**Min**	**Mean**	**Std**
	Fit	3.41 × 10${}^{-16}$	4.36 × 10${}^{-8}$	4.21 × 10${}^{-7}$	1.24 × 10${}^{-13}$	2.90 × 10${}^{-6}$	1.25 × 10${}^{-5}$	3.38 × 10${}^{-12}$	7.94 × 10${}^{-6}$	4.49 × 10${}^{-5}$	3.25 × 10${}^{-11}$	7.38 × 10${}^{-6}$	6.87 × 10${}^{-5}$
	MAE	1.36 × 10${}^{-12}$	3.16 × 10${}^{-6}$	2.59 × 10${}^{-5}$	5.46 × 10${}^{-12}$	3.95 × 10${}^{-5}$	2.00 × 10${}^{-4}$	9.00 × 10${}^{-11}$	3.78 × 10${}^{-5}$	3.15 × 10${}^{-4}$	7.00 × 10${}^{-11}$	1.67 × 10${}^{-5}$	5.57 × 10${}^{-5}$
Scenario II	TIC	1.53 × 10${}^{-13}$	3.45 × 10${}^{-7}$	2.75 × 10${}^{-6}$	5.88 × 10${}^{-13}$	4.33 × 10${}^{-6}$	2.14 × 10${}^{-5}$	9.90 × 10${}^{-12}$	4.26 × 10${}^{-6}$	3.45 × 10${}^{-5}$	8.96 × 10${}^{-12}$	2.21 × 10${}^{-6}$	7.19 × 10${}^{-6}$
	RMSE	1.12 × 10${}^{-12}$	2.52 × 10${}^{-6}$	2.01 × 10${}^{-5}$	4.11 × 10${}^{-12}$	3.03 × 10${}^{-5}$	1.50 × 10${}^{-4}$	6.66 × 10${}^{-11}$	2.86 × 10${}^{-5}$	2.32 × 10${}^{-4}$	5.82 × 10${}^{-11}$	1.43 × 10${}^{-5}$	4.67 × 10${}^{-5}$
	ENSE	0	5.29 × 10${}^{-9}$	5.20 × 10${}^{-8}$	0	3.55 × 10${}^{-7}$	2.01 × 10${}^{-6}$	0	9.40 × 10${}^{-7}$	9.33 × 10${}^{-6}$	0	3.39 × 10${}^{-8}$	1.95 × 10${}^{-7}$
**Cases**			γ=0.0			γ=0.1			γ=0.2			γ=0.3	
		**Min**	**Mean**	**Std**	**Min**	**Mean**	**Std**	**Min**	**Mean**	**Std**	**Min**	**Mean**	**Std**
	Fit	6.06 × 10${}^{-12}$	6.49 × 10${}^{-6}$	4.46 × 10${}^{-5}$	5.34 × 10${}^{-12}$	6.01 × 10${}^{-6}$	4.09 × 10${}^{-5}$	4.65 × 10${}^{-12}$	2.43 × 10${}^{-6}$	2.41 × 10${}^{-5}$	3.99 × 10${}^{-12}$	6.22 × 10${}^{-6}$	3.55 × 10${}^{-5}$
	MAE	8.68 × 10${}^{-11}$	1.01 × 10${}^{-4}$	6.52 × 10${}^{-4}$	1.25 × 10${}^{-10}$	8.55 × 10${}^{-6}$	3.84 × 10${}^{-5}$	1.69 × 10${}^{-11}$	2.26 × 10${}^{-6}$	4.83 × 10${}^{-6}$	4.86 × 10${}^{-12}$	2.13 × 10${}^{-6}$	5.25 × 10${}^{-6}$
Scenario III	TIC	9.25 × 10${}^{-12}$	1.06 × 10${}^{-5}$	6.76 × 10${}^{-5}$	1.33 × 10${}^{-11}$	1.02 × 10${}^{-6}$	4.48 × 10${}^{-6}$	2.08 × 10${}^{-12}$	2.80 × 10${}^{-7}$	5.68 × 10${}^{-7}$	5.60 × 10${}^{-13}$	2.64 × 10${}^{-7}$	6.37 × 10${}^{-7}$
	RMSE	6.50 × 10${}^{-11}$	7.42 × 10${}^{-5}$	4.74 × 10${}^{-4}$	9.38 × 10${}^{-11}$	7.19 × 10${}^{-6}$	3.17 × 10${}^{-5}$	1.48 × 10${}^{-11}$	1.99 × 10${}^{-6}$	4.04 × 10${}^{-6}$	4.01 × 10${}^{-12}$	1.89 × 10${}^{-6}$	4.56 × 10${}^{-6}$
	ENSE	0	3.68 × 10${}^{-6}$	2.58 × 10${}^{-5}$	0	1.30 × 10${}^{-8}$	8.56 × 10${}^{-8}$	0	2.35 × 10${}^{-10}$	1.77 × 10${}^{-9}$	0	2.61 × 10${}^{-10}$	1.68 × 10${}^{-9}$
	**Cases**		***Q* = 0.0**			***Q* = 0.3**			***Q* = 0.6**			***Q* = 1.2**	
		**Min**	**Mean**	**Std**	**Min**	**Mean**	**Std**	**Min**	**Mean**	**Std**	**Min**	**Mean**	**Std**
	Fit	2.88 × 10${}^{-12}$	1.03 × 10${}^{-5}$	7.12 × 10${}^{-5}$	8.56 × 10${}^{-12}$	9.44 × 10${}^{-6}$	6.39 × 10${}^{-5}$	9.68 × 10${}^{-12}$	2.75 × 10${}^{-5}$	1.00 × 10${}^{-4}$	7.47 × 10${}^{-12}$	1.03 × 10${}^{-5}$	5.80 × 10${}^{-5}$
	MAE	5.76 × 10${}^{-11}$	6.26 × 10${}^{-6}$	1.79 × 10${}^{-5}$	8.97 × 10${}^{-12}$	1.24 × 10${}^{-5}$	4.54 × 10${}^{-5}$	7.82 × 10${}^{-12}$	7.44 × 10${}^{-6}$	3.12 × 10${}^{-5}$	6.76 × 10${}^{-11}$	8.43 × 10${}^{-6}$	3.03 × 10${}^{-5}$
Scenario IV	TIC	6.42 × 10${}^{-12}$	8.27 × 10${}^{-7}$	2.29 × 10${}^{-6}$	1.10 × 10${}^{-12}$	1.59 × 10${}^{-6}$	5.71 × 10${}^{-6}$	9.11 × 10${}^{-13}$	9.48 × 10${}^{-7}$	3.84 × 10${}^{-6}$	7.17 × 10${}^{-12}$	1.04 × 10${}^{-6}$	3.63 × 10${}^{-6}$
	RMSE	4.20 × 10${}^{-11}$	5.42 × 10${}^{-6}$	1.50 × 10${}^{-5}$	7.34 × 10${}^{-12}$	1.06 × 10${}^{-5}$	3.82 × 10${}^{-5}$	6.22 × 10${}^{-12}$	6.48 × 10${}^{-6}$	2.62 × 10${}^{-5}$	5.00 × 10${}^{-11}$	7.27 × 10${}^{-6}$	2.54 × 10${}^{-5}$
	ENSE	0	3.54 × 10${}^{-9}$	1.88 × 10${}^{-8}$	0	2.08 × 10${}^{-8}$	1.27 × 10${}^{-7}$	0	9.27 × 10${}^{-9}$	8.18 × 10${}^{-8}$	0	8.53 × 10${}^{-9}$	6.85 × 10${}^{-8}$

**Table 19:** Comparison of percentage convergent runs of the ENN-GNDO-IPA algorithm achieving different levels of fitness value, MAE, TIC, RMSE, and ENSE.

		Fit			MAE			TIC			RMSE			ENSE	
	≤$10^{-7}$	≤$10^{-8}$	$\leq 10^{-9}$	≤$10^{-6}$	≤$10^{-7}$	≤$10^{-8}$	≤$10^{-6}$	≤$10^{-7}$	≤$10^{-8}$	$\leq 10^{-6}$	≤$10^{-7}$	$\leq 10^{-8}$	≤$10^{-8}$	≤$10^{-9}$	≤$10^{-10}$
β=0.0	95	69	62	62	61	61	98	62	61	62	61	61	98	95	62
β=0.3	94	94	50	94	50	50	97	82	50	94	50	50	97	96	94
β=0.6	97	94	93	97	50	46	100	97	50	97	50	46	100	98	97
β=1.2	97	95	89	97	87	48	99	97	87	97	97	45	99	99	97
$S_{h}=0.25$	99	98	98	98	98	44	99	98	94	98	94	44	99	99	99
$S_{h}=0.50$	93	89	84	87	47	47	95	87	48	87	47	47	97	95	95
$S_{h}=0.75$	96	93	62	95	54	54	98	93	54	95	54	54	99	98	95
$S_{h}=1.00$	92	91	58	62	52	52	93	61	53	62	52	52	96	93	62
γ=0.0	95	91	59	91	50	50	97	91	51	91	50	50	97	91	91
γ=0.1	94	92	83	94	56	56	98	94	56	94	56	56	98	96	94
γ=0.2	99	98	93	99	51	52	100	99	51	99	51	52	100	100	99
γ=0.3	97	95	94	98	55	52	100	98	73	98	55	52	100	99	99
Q=0.0	95	95	63	95	53	53	97	68	54	95	53	53	97	97	96
Q=0.1	91	90	63	92	58	58	97	88	59	92	58	58	98	92	92
Q=0.2	90	88	52	95	50	50	99	95	49	95	50	50	99	95	95
Q=0.3	95	88	55	91	49	49	99	91	52	91	49	49	99	92	91

**Table 20:** Unknown parameters obtained by the proposed algorithm for optimization of different cases of scenario I of problem 3.

		β=0.0			β=0.3			β=0.6			β=1.2	
	$\alpha_{i}$	$\xi_{i}$	$\beta_{i}$	$\alpha_{i}$	$\xi_{i}$	$\beta_{i}$	$\alpha_{i}$	$\xi_{i}$	$\beta_{i}$	$\alpha_{i}$	$\xi_{i}$	$\beta_{i}$
1	1.75506255			−0.2280457			1.73509127			−0.6635142		
2	−0.0397538	−0.8540435	−4.6498181	−0.9511576	−1.5710715	1.38596977	−0.2742883	4.99966028	−2.36443034	0.009853106	−1.56853219	1.662808179
3	1.1725275	1.27301519	−1.4969907	−0.0947595	−1.9225336	1.59694342	−4.3793514	0.26168972	2.677038378	1.005722437	0.193732802	−0.71156842
4	1.03654752	−0.3632161	4.23843219	0.46085221	0.97699876	−0.9337255	0.60918163	−0.8727691	−1.90790441	−1.2003581	−0.38710432	0.602348099
5	4.99020759	−0.3560224	0.70022227	−0.7282585	0.19518206	−0.9641863	0.02364475	−0.8740199	−3.942231	−4.05298776	−0.1292758	1.494375212
6	0.0624978	0.27027645	−3.5432088	0.99019111	−0.256195	2.20590469	−0.7924081	−0.1535354	−1.61428544	2.532561638	−0.15524836	1.596505589
7	0.43974599	0.42790519	3.31159218	0.56289436	0.327869	−4.4042753	0.47812532	0.27973968	3.040318662	0.69742436	0.193625812	−4.92925119

**Table 21:** Unknown parameters obtained by the proposed algorithm for optimization of different cases of scenario II of problem 3.

		β=0.0			β=0.3			β=0.6			β=1.2	
	$\alpha_{i}$	$\xi_{i}$	$\beta_{i}$	$\alpha_{i}$	$\xi_{i}$	$\beta_{i}$	$\alpha_{i}$	$\xi_{i}$	$\beta_{i}$	$\alpha_{i}$	$\xi_{i}$	$\beta_{i}$
1	−1.0680896			0.70101785			−1.343865			2.763393294		
2	0.40354062	−1.4090674	−0.9321023	0.86698071	−0.675388	−0.5212876	0.13127215	4.23827851	−4.40871712	3.156345477	4.700776808	−2.08561482
3	−0.862177	−0.5357362	0.16038354	0.56728693	0.05022776	1.4536595	−0.73727	−1.1895848	3.039628159	−0.09117495	4.9999867	−3.43408732
4	−0.7122253	−0.0286262	−0.522483	−0.4077234	−0.555023	−0.0118765	−0.5529075	−1.0479314	2.310600138	0.090009341	2.195807641	−4.7506135
5	−0.4429424	−0.5243682	0.00017912	−0.5020269	0.21836618	−0.9132638	2.08866814	−0.1015598	−0.52072196	−1.20616021	0.156240585	−2.49993549
6	−0.6093197	0.28496279	−0.1850434	−0.6005535	−0.3823589	1.24380756	−0.6560448	−0.4923354	2.021261453	−1.81123359	0.211749304	−1.95915534
7	1.80716535	0.15184791	4.38926823	1.6425367	−0.2128676	0.26031718	2.19171938	−0.2435099	−2.43351116	0.532855338	0.349904423	4.542668621

**Table 22:** Unknown parameters obtained by the proposed algorithm for optimization of different cases of scenario III of problem 3.

		γ=0.0			γ=0.1			γ=0.2			γ=0.3	
	$\alpha_{i}$	$\xi_{i}$	$\beta_{i}$	$\alpha_{i}$	$\xi_{i}$	$\beta_{i}$	$\alpha_{i}$	$\xi_{i}$	$\beta_{i}$	$\alpha_{i}$	$\xi_{i}$	$\beta_{i}$
1	−2.8348221			1.50375512			3.52637262			0.209596131		
2	0.49488715	−0.9329544	2.23901769	−0.9377929	−1.8733799	−1.4606246	3.75374516	1.66359944	−2.02557049	−1.29575683	−0.99399768	3.45170921
3	0.98998192	−2.1537236	3.71326101	3.26458001	0.46500557	−4.8051945	1.02342208	−0.1523187	2.141850069	0.282634136	0.206500932	−2.50334716
4	0.44167549	−1.0311619	1.01252798	−0.4662263	−0.9696201	2.99669645	−0.0358419	−2.345193	4.999480973	0.006592178	0.618362414	−2.33595055
5	−0.2262942	1.00304712	−2.5329297	−0.4203961	0.25347969	−3.8747122	−0.9175584	−0.197368	−1.01467736	1.176986878	0.36495718	0.525501182
6	−4.9857823	−0.3162414	0.55726994	−0.2350521	0.28676082	−2.64615	2.91760809	−0.1786006	2.140963484	0.800908761	−0.07966475	2.105286189
7	2.15973111	−0.2363378	3.80973811	0.44620088	−0.3038427	−4.8659554	0.56685993	0.28833385	4.224463971	0.488909695	0.291552938	−0.68743993

**Table 23:** Unknown parameters obtained by the proposed algorithm for optimization of different cases of scenario IV of problem 3.

		Q=0.0			Q=0.1			Q=0.2			Q=0.3	
	$\alpha_{i}$	$\xi_{i}$	$\beta_{i}$	$\alpha_{i}$	$\xi_{i}$	$\beta_{i}$	$\alpha_{i}$	$\xi_{i}$	$\beta_{i}$	$\alpha_{i}$	$\xi_{i}$	$\beta_{i}$
1	2.12610112	0	0	−0.8351889	0	0	−0.2435167	0	0	−4.01040396	0	0
2	1.91015485	−4.9577883	0.41606348	−0.3764984	−0.074764	−0.4518613	−4.9995641	3.16431335	−2.23768782	−3.71041538	−2.4824238	1.334293947
3	1.27282106	1.34476072	0.84496967	1.96958577	−0.1932274	0.81872283	0.81658139	−1.3703791	−2.61415334	−1.43426164	−0.131799	−4.63109851
4	2.03701973	−0.5211284	3.76126915	−0.9736603	−0.3673783	−0.1681137	−0.3062593	−0.5235023	2.858011068	3.907586578	−0.17368062	3.088951839
5	−1.7568706	−0.3783811	3.1343742	0.02363823	1.13161649	−0.6509107	−0.998816	0.15542115	−1.71782147	−4.99852588	0.101776099	−1.09616623
6	−4.0002204	0.22848117	−0.7749328	0.38691585	0.52978769	−0.7559981	1.44224716	−0.2885232	1.011495284	0.106588537	−0.42136974	1.117170635
7	0.68959286	0.31502173	4.63672578	1.75593225	−0.2617437	4.54924612	0.78443172	0.29050015	3.66642011	0.000770587	−0.89383904	−4.11527632

**Table 24:** Comparison of approximate solutions obtained by proposed algorithm with the Adomian decomposition method and the Runge–Kutta method.

		β=−0.3			β=0.0			β=0.3	
x	ADM	RK-4	ENN-GNDO-SQP	ADM	RK-4	ENN-GNDO-SQP	ADM	RK-4	ENN-GNDO-SQP
0	1	1	1	1	1	1	1	1	1
0.1	0.9147	0.9157	0.9157	0.9355	0.9355	0.9355	0.9512	0.9477	0.9477
0.2	0.8469	0.8483	0.8483	0.882	0.882	0.882	0.9102	0.9036	0.9036
0.3	0.7931	0.7942	0.7942	0.8379	0.8379	0.8379	0.8761	0.8668	0.8668
0.4	0.751	0.7511	0.7511	0.8018	0.8018	0.8018	0.8481	0.8365	0.8365
0.5	0.7186	0.7172	0.7172	0.773	0.773	0.773	0.8256	0.8119	0.8119
0.6	0.6945	0.6911	0.6911	0.7504	0.7504	0.7504	0.808	0.7926	0.7926
0.7	0.6776	0.6718	0.6718	0.7336	0.7336	0.7336	0.795	0.7782	0.7782
0.8	0.667	0.6587	0.6587	0.7221	0.7221	0.7221	0.7862	0.7682	0.7682
0.9	0.6612	0.6511	0.6511	0.7154	0.7154	0.7154	0.7811	0.7624	0.7624
1	0.6601	0.6486	0.6486	0.7132	0.7132	0.7132	0.7795	0.7605	0.7605

## Data Availability

The data that support the findings of this study are available from the corresponding author upon reasonable request.

## Nomenclature

ENN	Euler neural network
ρ	Specific heat density
GNDO	Generalized normal distribution optimization
$T_{i}$	Initial temperature
MAD	Mean absolute deviation
*C*	Specific heat
TIC	Theil’s inequality coefficient
$T_{s}$	Sink temperature
NSE	Nash–Sutcliffe efficiency
*t*	Thickness of porous fin
ENSE	Error in Nash–Sutcliffe efficiency
v(x)	Velocity of buoyancy flow
IPA	Interior point algorithm
*L*	Length of porous fin
RMSE	Root mean square error
$\bar{\zeta }$	Constant
DTM	Differential Transform Method
$B_{k}$	Bernoulli’s number
ADM	Adomian decomposition method
$a_{i}^{t}$	Trail Vector
*h*	Coefficient of convective heat transfer
η	Penalty factor
HPM	Homotopy perturbation Method
$d_{i}$	Standard Variance
